# Electrochemical Generation of Aryl Radicals from Organoboron Reagents Enabled by Pulsed Electrosynthesis

**DOI:** 10.1002/anie.202406203

**Published:** 2024-06-25

**Authors:** Maxime Boudjelel, Jessica Zhong, Lorenzo Ballerini, Ian Vanswearingen, Rossul Al-Dhufari, Christian A. Malapit

**Affiliations:** Department of Chemistry, Northwestern University, 2145 N Sheridan Road, Evanston, IL 60208 USA

**Keywords:** electrosynthesis, organoboron reagents, aryl radicals, pulsed electrochemistry

## Abstract

Aryl radicals play a pivotal role as reactive intermediates in chemical synthesis, commonly arising from aryl halides and aryl diazo compounds. Expanding the repertoire of sources for aryl radical generation to include abundant and stable organoboron reagents would significantly advance radical chemistry and broaden their reactivity profile. While traditional approaches utilize stoichiometric oxidants or photocatalysis to generate aryl radicals from these reagents, electrochemical conditions have been largely underexplored. Through rigorous mechanistic investigations, we identified fundamental challenges hindering aryl radical generation. In addition to the high oxidation potentials of aromatic organoboron compounds, electrode passivation through radical grafting, homocoupling of aryl radicals, and decomposition issues were identified. We demonstrate that pulsed electrosynthesis enables selective and efficient aryl radical generation by mitigating the fundamental challenges. Our discoveries facilitated the development of the first electrochemical conversion of aryl potassium trifluoroborate salts into aryl C–P bonds. This sustainable and straightforward oxidative electrochemical approach exhibited a broad substrate scope, accommodating various heterocycles and aryl chlorides, typical substrates in transition-metal catalyzed cross-coupling reactions. Furthermore, we extended this methodology to form aryl C–Se, C–Te, and C–S bonds, showcasing its versatility and potential in bond formation processes.

## Graphical Abstract

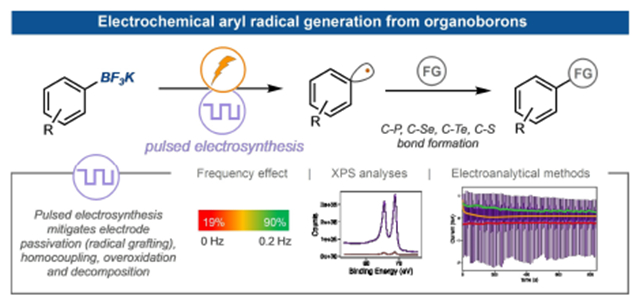

Pulsed electrosynthesis efficiently converts aromatic organoboron reagents to aryl radicals. Mechanistic studies reveal that pulsed electrosynthesis overcomes challenges like radical grafting/passivation, homocoupling, overoxidation, and decomposition. This electro-oxidative method enables straightforward functionalization of aromatic organoboron reagents to form aryl C–P, C–Se, C–Te, and C–S bonds.

***O***rganoboron reagents have become an important class of compounds that have found widespread application as substrates in chemical synthesis.^[1]^ As such, their commercial availability has significantly increased and synthetic methods to construct them have been highlights in many research pursuits.^[2–6]^ Despite the extensive research conducted over the past few decades into the chemistry of organoboron compounds,^[2–8]^ their general reactivity has been mostly dominated by transmetallation with various transition metals such as Pd, Ni, and Cu to yield productive functionalization of organoboron reagents to various carbon-carbon and carbon-heteroatom bonds (Figure 1). One interesting reactivity available for aromatic organoboron reagents is their one-electron oxidation to form aryl radicals that can engage in bond-forming reactions unique to those typically observed via transmetallation.^[9–12]^ Due to the high oxidation potentials of aryl boron reagents,^[13]^ methods that explores their reactivity to generate aryl radicals were confined to a very narrow set of chemical and photochemical oxidation processes. In most cases, the aryl radicals generated in these conditions were limited to Minisci-type and radical conjugate addition reactions to form carbon-carbon bonds.^[9,12]^

With the resurgence of electrochemistry as an innate way of performing redox reactions of various organic compounds to generate reactive intermediates,^[14–19]^ we sought to develop an electrochemical process that efficiently generates aryl radicals from organoboron reagents and promote various carbon-heteroatom bond formation. Here we report a systematic study that provides understanding of the fundamental challenges associated with the underexplored electrochemical aryl radical generation from organoboron reagents. More importantly, we discovered that the use of pulsed electrosynthesis enables the selective electrochemical functionalization of aromatic organoboron compounds to form various carbon-heteroatom bonds. Mechanistic investigations were performed to elucidate the striking significant effect of pulsed electrosynthesis over conventional electrolysis on the productive aryl radical generation for a straightforward electro-conversion of aryl trifluoroborate salts to C–P, C–Te, C–Se, and C–S bonds.

While there are sporadic examples of direct electrochemical transformations of aromatic organoboron reagents, their mechanism remains unclear.^[16,20–24]^ Recently, Stahl reported an electrochemical process using a redox mediator, however, this was limited to benzylic radical generation.^[25]^ As such, our initial studies have focused on uncovering the fundamental challenges (see below) associated with electrochemical one-electron oxidation of organoboron reagents to aryl radicals and their subsequent functionalization reactions.

Aryl boronic acids are known to have high oxidation potentials (Figure 2A), typically above 1.5 V (vs Fc/Fc^+^), and this has limited their utility in one-electron oxidation processes.^[13]^ However, the quaternization of the boron center to a boronate complex significantly lowers their oxidation potential and had found successful application in photoredox reactions.^[26]^ Our initial cyclic voltammetry studies (Figure 2B) using *p*-anisyl potassium trifluoroborate **1** on glassy carbon showed an accessible oxidation onset potential below 1.0 V (vs Fc/Fc^+^). Unfortunately, a drastic loss of current response was observed after successive CV scans using the same electrode. This suggests electrode passivation and will hinder further redox events on the electrode.

Nonetheless, we went on and initiated several electrochemical oxidation reactions using aryl-BF_3_K **1** as the substrate under constant current electrolysis (CCE). Triethylphosphite, P(OEt)_3_, was selected as the trapping reagent, being known for this purpose and allows a rapid assessment of the yield via ^31^P NMR.^[27–29]^ Initial conditions used dichloromethane as a solvent which would also act as a sacrificial reagent via cathodic reduction, graphite as working and counter electrodes, and TBAPF_6_ as the electrolyte (Figure 2C). These conditions had been shown to be successful for the electrochemical oxidation of alkyl organoboron reagents, although via a two-electron process.^[30]^ We were delighted to observe the formation of product **2**, albeit in trace amount (<5% yield). All attempts at trying to improve the yield such as increasing the P(OEt)_3_ concentration, the charge applied, the applied current, and solvent screen, did not show significant increase on the yield of **2**. Interestingly, in these reactions we observed very poor mass balance with only 10 to 50% of starting material detected after the reaction. In addition, 4,4-dimethoxybiphenyl was also observed as a byproduct in some cases, denoting the homocoupling of the aryl radical generated via one-electron oxidation (see Supporting Information for details). Upon screening various electrode materials to mitigate passivation, it was discovered that employing Pt as both working and counter electrodes led to the formation of product **2** in 20% yield. All other efforts aimed at enhancing the yield through constant current or potential electrolysis yielded no significant improvements. Nonetheless, these initial studies show that in addition to the high oxidation potential of aromatic boron reagents, electrode passivation due to aryl radical grafting,^[31–33]^ aryl radical homocoupling, and decomposition (presumably due to overoxidation and oligomerization)^[13,25,34,35]^ are fundamental challenges that limit the development of electrochemical one-electron oxidation and functionalization of aromatic organoboron reagents (Figure 2D).

We propose that the use of pulsed electrosynthesis, by applying an alternating current or potential across the electrodes, will mitigate the fundamentals challenges associated in the one-electron anodic oxidation of aryl trifluoroborate salts (Figure 2E). Changing the polarity of the electrode via pulsed electrolysis would refresh the electrode surface and therefore expulse the radical formed to the reaction media. This would theoretically avoid grafting, overoxidation/decomposition and homocoupling. The adoption of alternating polarity has seen significant traction in recent years, driving notable advancements in electrosynthesis. These advancements have led to improved selectivity in chemical processes^[36–38]^ and/or the preservation of electrode surfaces to prevent passivation.^[39,40]^

Using Pt plates as electrodes, we performed pulsed electrosynthesis by investigating the effect of frequency (Hz) on the reaction (Figure 2F). We found that 0.2 Hz was the ideal frequency for the model substrate with up to 2.5-fold increase in yield. To further improve the yield, we set up various reactions by changing several electro- and chemical parameters such as solvent, supporting electrolytes, applied current or potential, and others (Figure 2F, entries 1–16). We found that the reaction works well in the absence of an external supporting electrolyte as the Ar-BF_3_K substrate is charged, and the yield significantly increased when the molar concentration of Ar-BF_3_K is increased. Acetone is shown to be the ideal solvent, as it can also act as the sacrificial reagent that undergoes reduction (E_p/2_ of acetone is −2.0 V vs Fc/Fc^+^) and thus acting as an electron sink for the reaction while producing benign byproducts.^[41]^ Relatively high currents (10 mA) are tolerated and gave improved yields allowing short reaction times. The reaction reached its optimal yield at 4 F/mol resulting to 90% of **2** (entry 13). Notably, this is the first example of converting an aromatic organoboron to aryl phosphine via a one-electron process. The conversion of aromatic boron reagent to aryl C–P bond has been reported to be accomplished only via transition metal catalysis.^[42,43]^

The very significant impact of pulsed electrosynthesis (or alternating polarity, AP) over constant current/potential electrolysis in enabling aryl radical generation from Ar-BF_3_K led us to perform mechanistic investigations in detail (Figure 3). Under AP conditions, the reaction media during and after the reaction remained clear while a dark colored solution was observed under CCE. Together with low mass balance in CCE, we associate this coloration with substrate electrodeposition and decomposition being significant reaction pathways under CCE. Importantly, the Pt electrode surface under both conditions were significantly different after the reaction wherein many deposited materials are visible under CCE while a polished electrode was observed under AP conditions. Further analysis of the Pt electrode surface after electrolysis via X-ray photoelectron spectroscopy (XPS) gave two distinct differences (Figure 3B). The relative intensity of the platinum peaks shows a 24-fold higher Pt coverage on the electrode surface under AP over CCE. Second, fluorine and potassium were found to be the major elements on Pt electrode used under CCE denoting that initial electro-grafting of substrate is followed by decomposition on the electrode.

Next, we looked at electroanalytical methods to further evaluate the enabling effects of pulsed electrosynthesis. Chronoamperometric analyses were performed for a reaction done under a bulk CPE vs a potential-pulsed electrolysis at 0.2 Hz and the current response was recorded with time (Figure 3C). Under CPE, a significant drop in current was observed after 100 seconds and stayed very low throughout the electrolysis. This indicates significant electrode passivation. Interestingly, under pulsed electrolysis, the average current remained constant throughout the electrolysis. Moreover, a scan-rate dependent cyclic voltammetry experiment was also performed using Pt working electrode (Figure 3D). At fast scan rate (2000 mV/s), no current loss was observed after 35 cycles, while at lower scan rate (200 mV/s), a current loss was observed during the first 35 cycles, denoting some passivation under lower frequencies. This explains the optimal frequency necessary for this system where faster frequencies at 0.2–1.0 Hz gave better yields over lower frequencies (0.02–0.1 Hz). It is interesting to note that the current loss in CV cycles with Pt electrode at 200 mV/s is significantly less than those observed with glassy carbon electrode (Figure 2B). If the reactions are conducted under very fast frequencies (2–10 Hz), we observed a significant decrease in yield (<5%) as well as very low conversions. We attribute this to the inefficient electrolysis as polarity switches too fast before the desired cell potentials are reached. The role of AP is clearly highlighted in these spectroscopic and electroanalytical studies where passivation of the electrode was avoided due to replenishment of the electrode surface during the reduction step on the same electrode.

We also looked at other well-known aryl radical traps such as 1,1-diphenylethylene to further our claim on radical generation (Figure 3E). Electrolysis of **1** in the presence of 1,1-diphenylethylene gave the C–C bond adduct as observed by GCMS.

Overall, we propose a mechanism, together with the effects of pulsed electrosynthesis, delineated in Figure 3F. Initially, the Ar-BF_3_K salt undergoes anodic oxidation to produce aryl radicals while acetone is concurrently reduced at the counter electrode. During CCE, the aryl radical can either graft to the electrode and decompose, causing electrode passivation, or undergo homocoupling. Pulsed electrolysis, however, alternates the electrode polarities, resulting in the formation of a new electric double layer. This phenomenon effectively mitigates undesired processes and could promote chemical step involving aryl radical functionalization. Various radical traps were used to generate different C–X bonds. Triethylphosphite, P(OEt)_3_, a well-known aryl radical trap, engages with the aryl radical to form an aryl phosphoranyl radical that can either undergo fragmentation to form the product and an ethyl radical or oxidation followed by ethyl elimination. Moreover, acetone plays a dual role as solvent and sacrificial reagent that undergoes reduction. The reduction of acetone to ketyl radical leads to homocoupling that eventually forms a pinacol as observed by GCMS.

The use of pulsed electrosynthesis as an enabling approach to generate aryl radical from organoboron reagent was further investigated to examine the scope this reactivity (Figure 4). Various substituted aryl-BF_3_K salts with varying electronic effects were found to be effective under the conditions optimized for 1 by using constant current/potential electrolysis. Ortho-substituted substrates were also observed to provide C–P bonds. Aryl chlorides (**9**) are well tolerated, a notable advantage of this oxidative functionalization process as aryl chlorides are common substrates in many cross-coupling reactions. Several heteroaryl substrates including benzothiophene, benzofuran, quinolines, and pyridines exhibited high compatibility under these electrochemical conditions. Moreover, various radical trap reagents were also utilized such as diaryl diselenides and ditellurrides, and dialkyl disulfides to convert (hetero)aryl-BF_3_K salts to (hetero)aryl C–Se, C–Te, and C–S bonds in moderate to good yields without substantial modification on the reaction conditions.

In summary, we have established a highly effective protocol for the electrochemical modification of aromatic boron reagents via one-electron oxidation. This reactivity was made possible by employing pulsed electrosynthesis, effectively addressing the intrinsic challenges associated with efficient anodic one-electron oxidation to aryl radicals as elucidated in this investigation. These challenges encompass electrode passivation, aryl radical dimerization, and decomposition through excessive oxidation. Through systematic exploration of pulse frequency and electrochemical parameters, we have refined this process into a robust and environmentally sustainable strategy for functionalizing aryl-BF_3_K salts, yielding aryl C–P bonds. Moreover, in-depth mechanistic inquiries have provided valuable insights into the advantages of pulsed electrosynthesis compared to conventional electrochemical methods, guiding the broadening of substrate scope to encompass diverse (hetero)aryl-BF_3_K salts, as well as facilitating the formation of C–Se, C–Te, and C–S bonds. The development of other transformations utilizing this efficient aryl radical generation via pulsed electrochemistry is currently under investigation in our laboratory.

The authors have cited additional references within the Supporting Information.^[44–57]^

## Supplementary Material

Supplementary material

## Figures and Tables

**Figure 1.**
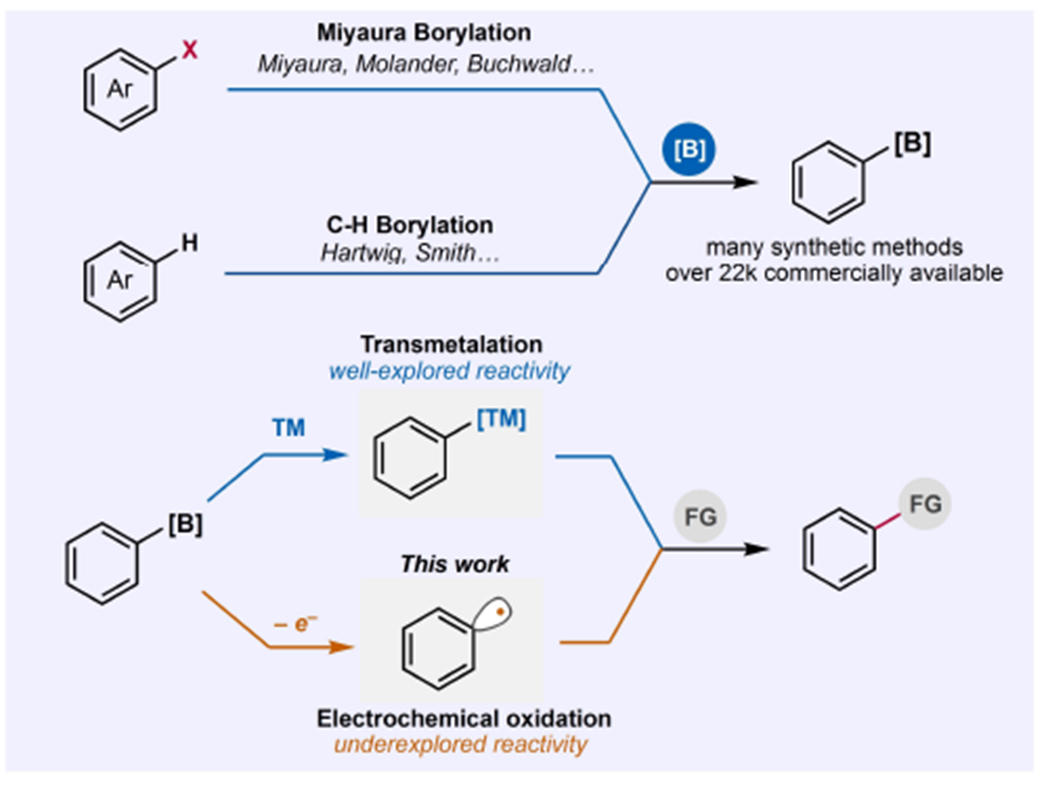
Synthesis and reactivity of aryl boron reagents via the well explored transmetallation and underexplored one-electron oxidation to aryl radicals using anodic oxidation (this work).

**Figure 2.**
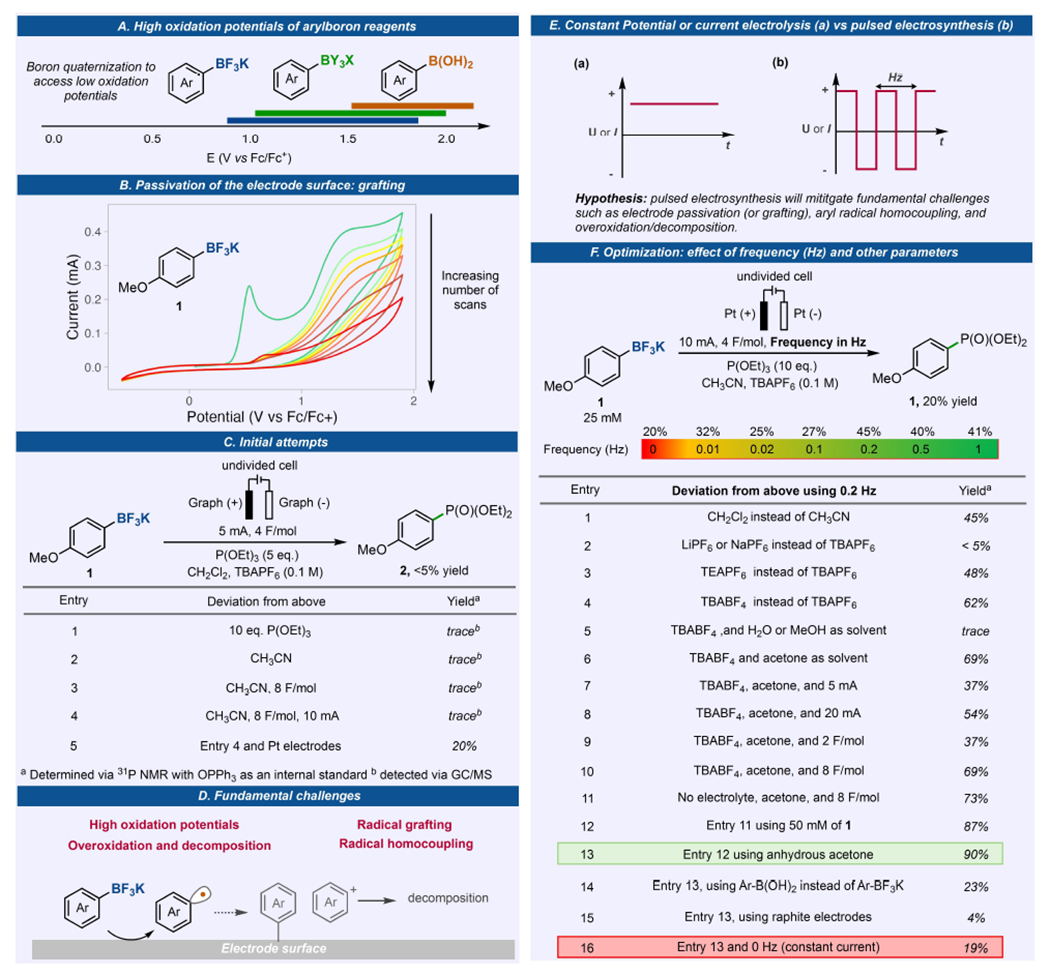
Enabling electrochemical generation of aryl radicals from organoboron reagents via understanding fundamental challenges and utility of pulsed electrosynthesis. (A) Oxidation potential of aryl organoboron reagents. (B) CV conditions: 1.0 mM 1, in 0.1 M TBABF_4_ in acetone, 200 mV/s, glassy carbon as WE. (C) Initial attempts for electrosynthetic generation of aryl radicals from 1. (D) Fundamental challenges encountered. (E) Alternating polarity waveform. (F) Effect of frequency and optimization of the reaction conditions, see Supporting Information, Figure S4 and Table S2, for details.

**Figure 3.**
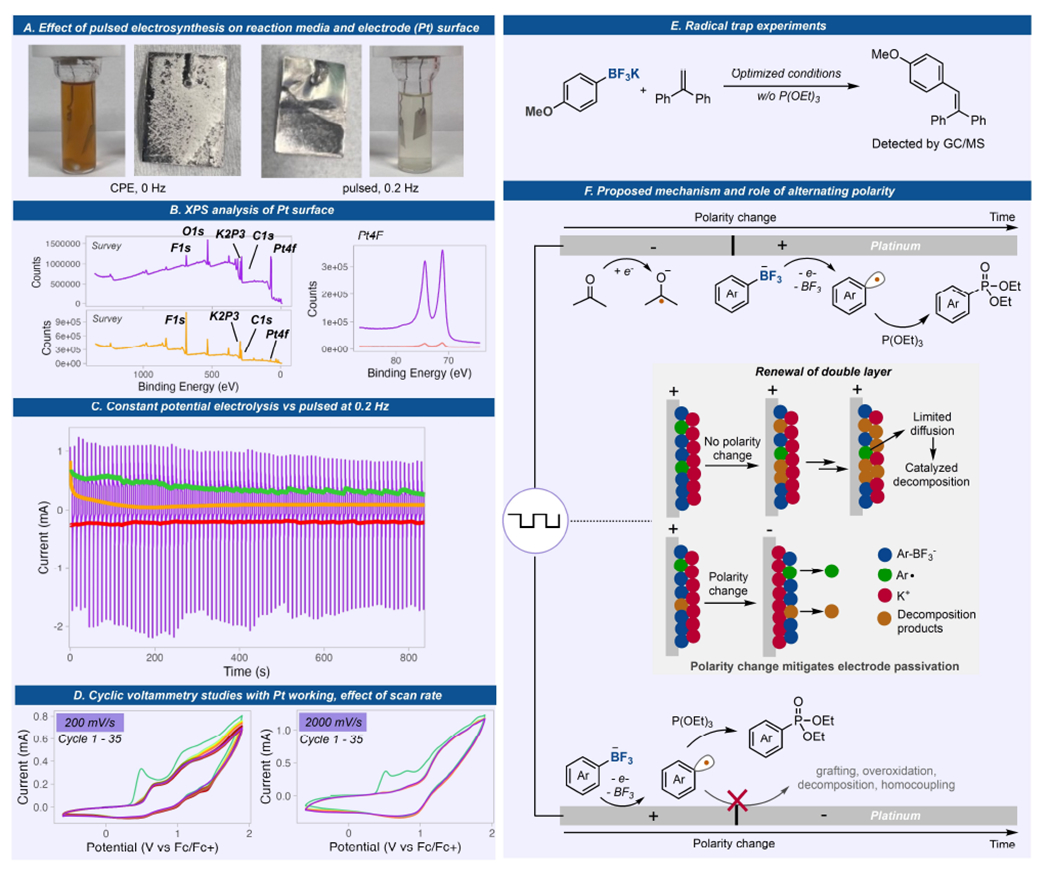
Mechanistic studies to understand the significant effect of pulsed electrosynthesis over CPE. (A) Pictures of reaction media and electrodes after reaction, without AP and with. (B) XPS analyses of electrodes without and with AP. (C) Chronoamperometry at 1.6 V (vs Fc/Fc+) under constant potential (orange) or alternating potential (purple) and the plots of the moving average of the oxidative (green) and reductive (red) currents. (D) Effect of scan rate on cyclic voltammograms of **1** with a Pt working electrode. (E) Radical trap experiment with TEMPO and 1,1-diphenylethylene. (F) Proposed mechanism and effect of pulsed electrosynthesis on aryl radical generation.

**Figure 4.**
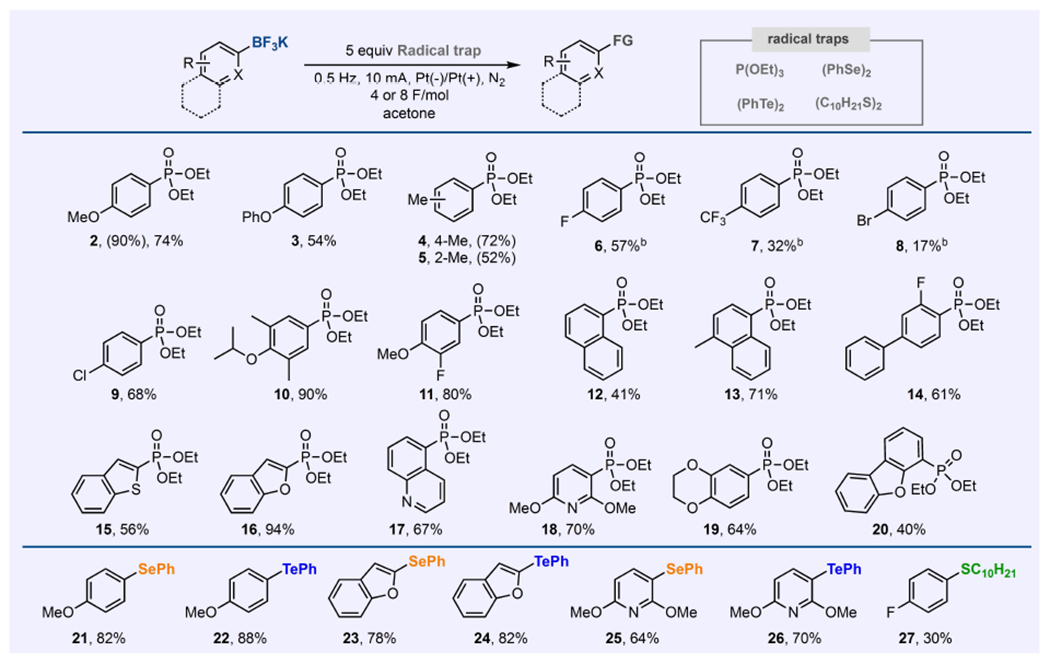
Scope of the electrochemical oxidative functionalization of aryl-BF_3_K salts to form C–P, C–Se, and C–Te bonds. ^a^ General reaction conditions, unless noted: 0.2 mmol of aryl-BF_3_K salt in acetone (4 mL), 5–10 equiv. of radical trap, electrolysis at alternating current (10 mA at 0.2 Hz). Yields are isolated yields. Yields in parentheses were obtained based on ^1^H or ^31^P NMR analysis of the crude mixture. See the Supporting Information, pages S11–S22, for details.

## Data Availability

The data that support the findings of this study are available in the supplementary material of this article.
