# Supplementary material for: Electrochemical Generation of Aryl Radicals from Organoboron Reagents Enabled by Pulsed Electrosynthesis
Source: Angew Chem Int Ed Engl. Author manuscript; Available in PMC 2024 Aug 14. (PMC11323302; doi:10.1002/anie.202406203)
Supplement: Supplementary material [file NIHMS2008056-supplement-Supplementary_material.pdf]

Angewandte Chemie
Eine Zeitschrift der Gesellschaft Deutscher Chemiker

Supporting Information

Electrochemical Generation of Aryl Radicals from Organoboron Reagents Enabled by Pulsed Electrosynthesis

*M. Boudjelel, J. Zhong, L. Ballerini, I. Vanswearingen, R. Al-Dhufari, C. A. Malapit**

Supporting Information for

Electrochemical generation of aryl radicals from organoboron reagents enabled by pulsed electrosynthesis

Maxime Boudjelel, Jessica Zhong, Lorenzo Ballerini, Ian Vanswearingen, Rossul Al-Dhufari and Christian A. Malapit*

Department of Chemistry, Northwestern University, Evanston, Illinois 60208, United States

Email: christian.malapit@northwestern.edu

Contents

1. Materials and methods **S1**

2. Electrochemical studies **S3**

2.1 Cyclic voltammetry **S3**

2.2 Constant potential electrolysis and alternating polarity constant potential electrolysis (CPE and AP-CPE) **S3**

2.3 Nyquist plot **S4**

3. Electrosynthesis: initial studies and optimization **S5**

3.1 Initial studies **S5**

3.2 Use of alternating polarity **S6**

3.3 Optimization of reaction conditions at 0.2 Hz **S6**

4. X-Ray photoelectron spectroscopy (XPS) analysis **S8**

5. Radical trap experiments **S10**

6. Substrate scope **S11**

7. Copies of ^{1}H, ^{13}C, ^{31}P and other heteronuclear NMR spectra **S24**

8. References **S50**

1. Materials and Methods

Unless otherwise noted, all manipulations were carried out using standard Schlenk or glovebox techniques under a N_2 atmosphere. Tetrahydrofuran, diethyl ether, dichloromethane, and toluene were dried and deoxygenated by argon purge followed by passage through activated alumina in a solvent purification system followed by storage over 4 Å molecular sieves. Organic trifluoroborate salts were purchased from Ambeed or Sigma-Aldrich and used as received or prepared according to published procedure. Triethylphosphite was purchased from Sigma-Aldrich, diphenyldiselenium and diphenylditelluride were purchased from Ambeed and were used a received. Deuterated solvents were purchased from Cambridge Isotope Laboratories Inc. Cyclic voltammetry were recorded using Biologic SP–50e, glassy carbon electrodes purchased from BASi inc. Electrosynthesis were performed using IKA ElectraSyn 2.0 using platinum plate electrodes purchased from Surepure Chemetals. CGMS analysis were recorded on TRACE 1610 (GC)–ISQ 7610 (Single Quadrupole MS) by ThermoFisher Scientific. NMR spectra were recorded on Bruker Avance III HD 500 MHz spectrometer. ^{1}H and ^{13}C chemical shifts are reported in ppm relative to tetramethylsilane using residual solvent as an internal standard. ^{19}F and ^{31}P chemical shifts are reported in ppm relative to respectively fluorobenzene and triphenyl phosphine oxide as external standards.

2. Electrochemical studies

2.1 Cyclic voltammetry

Cyclic voltammetry of potassium 4-methoxyphenyltrifluoroborate (1 mM) was recorded in acetonitrile with tetrabutylammonium tetra-fluoroborate (0.1M) as a supporting electrolyte. A glassy carbon working electrode (3 mm disc), a platinum wire counter electrode and a frit body (filled with the electrolyte solution) containing a silver wire (coated with silver) as reference electrode were used. A scan rate of 200 mV/s was used. Ferrocene was used as an internal standard to reference the potentials.

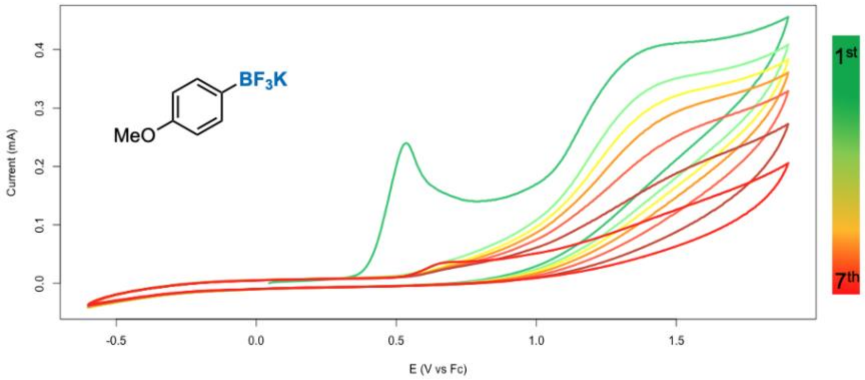

Figure S1. Cyclic voltammogram of potassium 4-methoxyphenyltrifluoroborate **1**, seven consecutive scans are shown.

2.2 Constant Potential Electrolysis and Alternating Polarity Constant Potential Electrolysis (CPE and AP-CPE)

Constant potential electrolysis of potassium 4-methoxyphenyltrifluoroborate (1 mM) was recorded in acetonitrile with tetrabutylammonium tetra-fluoroborate (0.1M) as a supporting electrolyte. A platinum working electrode (3 mm disc), a platinum wire counter electrode and a frit body (filled with the electrolyte solution) containing a silver wire (coated with silver) as reference electrode were used. A voltage of 1.4 V/Fc was used. For the alternating polarity electrolysis, potential was alternated between 1.4 and –1.4 V/Fc with a period of 0.2 Hz. The moving average (k = 55, using “rollmean” command in R from the “zoo” library) of the oxidative current was plotted for clarity.

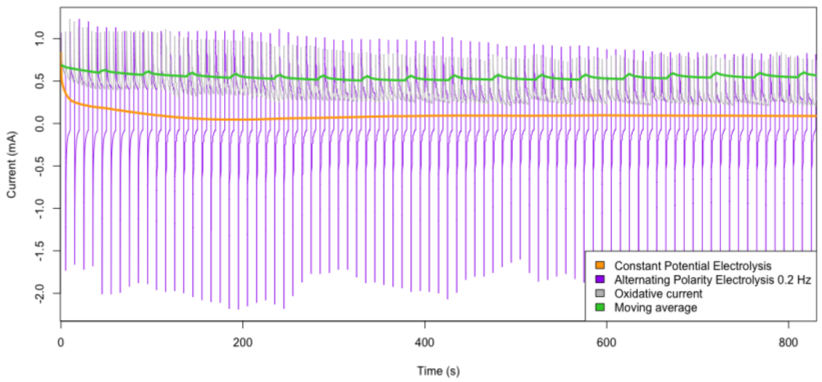

Figure S2. CPE vs AP electrolysis plot of current over time.

2.3 Nyquist Plot

Electrochemical Impedance Spectroscopy was used to plot Nyquist plot of the working platinum electrode after being used in constant potential electrolysis and alternating polarity electrolysis (10 kHz – 20 mHz, amplitude 10 mV rms). It was unfortunately not possible to fit a circuit to the plots, but the overall impedance of the media was dramatically increased after using constant potential electrolysis. Interestingly, the media presents a lower impedance after alternating polarity electrolysis than an unused electrode in the same media.

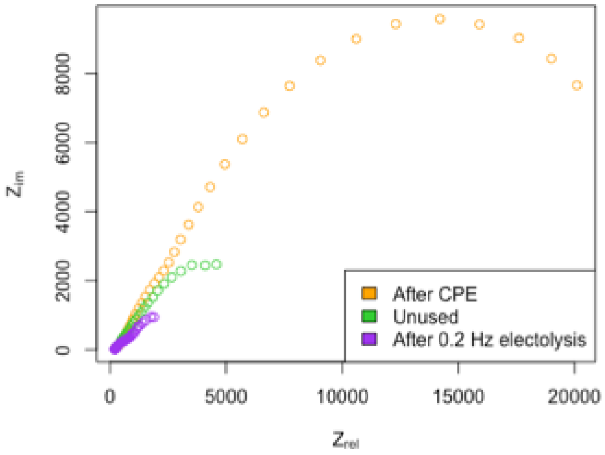

Figure S3. Nyquist plot of Pt working electrode.

3. Electrosynthesis: initial studies and optimization

3.1 Initial studies

Potassium 4-methoxyphenyltrifluoroborate (**1**) was used as a model substrate for optimization. In an IKA Electrasyn vial (5 mL), 1 (0.1 mmol) was weighed and DCM (4 mL) was added. To this solution, triethylphosphite was added (5-10 eq, 0.5-1.0 mmol). The vial was capped using an ElectraSyn vial cap that is fitted with a septa and two electrodes (working and counter electrodes) and the solution was bubbled with nitrogen. A balloon with nitrogen was then placed on the septa. The electrochemical set-up was attached to the ElectraSyn 2.0 and electrolysis was conducted at a current of 10 mA for 4 F/mol with stirring.

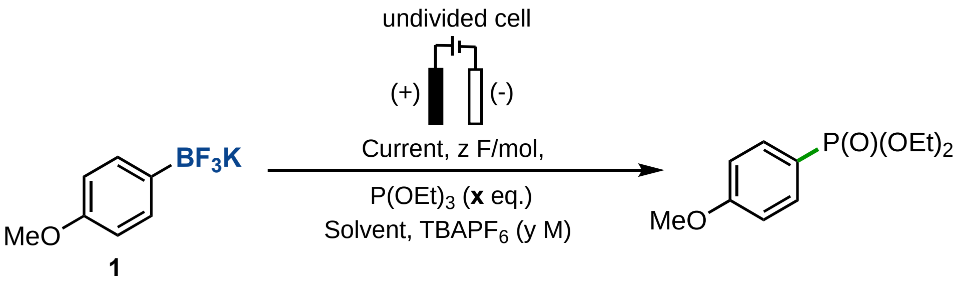

Solvent	x	y	Electrodes	Current (mA)	z	Yield (NMR vs Ph_3PO)
CH_2Cl_2	5	0.1	Graphite	5	4	trace
CH_2Cl_2	10	0.1	Graphite	5	4	trace
CH_3CN	5	0.1	Graphite	5	4	trace
CH_2Cl_2	5	0.1	Graphite	5	8	trace
CH_2Cl_2	5	0.1	Graphite	10	4	trace
CH_2Cl_2	5	0.1	Platinum	5	4	19%
CH_3CN	10	0.1	Graphite	10	4	4%
CH_3CN	10	0.1	RVC	10	4	trace
CH_3CN	10	0.1	Ni foam	10	4	trace
CH_3CN	10	0.1	Platinum	10	4	20%
Acetone	0	0	Platinum	4	2	0%[a], homocoupling observed

Table S1. Initial studies and optimization of the reaction using constant current electrolysis. [a]The BF_3K salt (0.1 mmol) was dissolved in acetone (0.7 mL) in an HTe-chem vial (1 mL). No triethyl phosphite or electrolyte was added. Platinum rod electrodes were used. Electrolysis was conducted at 4 mA for 2 F/mol. Homocoupling byproduct, 4,4-dimethoxybiphenyl was detected by GC/MS.

3.2 Use of Alternating Polarity

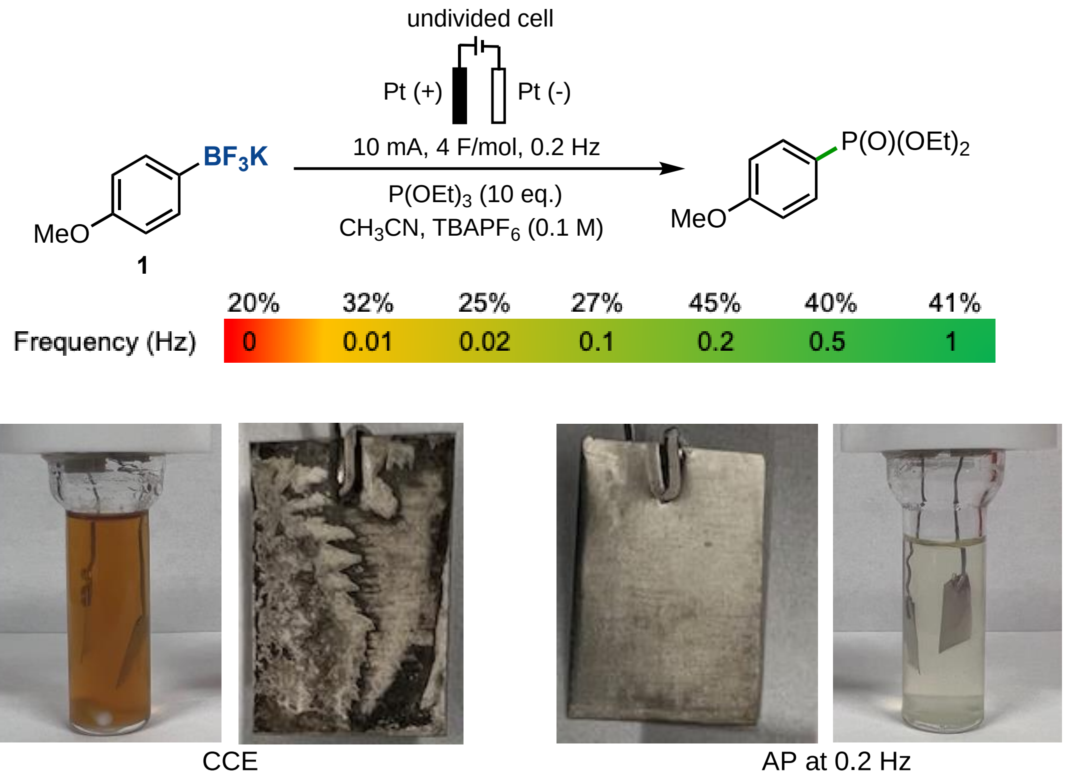

Figure S4a. Initial optimization using pulsed electrosynthesis. Reactions were run using different frequencies. The effect on the reaction media and electrodes after electrolysis were compared using 0 H and reactions ran under pulsed (alternating polarity, AP) at 0.2 Hz.

The left side depicts the reaction media at the end of the reaction without alternating polarity, and a zoom on the respective electrodes. The right side depicts the reaction using alternating polarity. The coloration seems to be brought by desorption of decomposition products forming on the Platinum working electrode. Unfortunately, in both cases, a large loss of mass balance was observed. No by-products could be characterized, and the conversion was tedious to track. But alternating polarity minimizes passivation on the electrode, and probable formation of hard to track oligomers. Conditions were then optimized using alternating polarity.

3.3 Optimization at 0.2 Hz

Potassium 4-methoxyphenyltrifluoroborate (**1**) was used as a model substrate for optimization. In an IKA Electrasyn vial (5 mL), 1 (0.1 mmol) was weighed and DCM (4 mL) was added. To this solution, triethylphosphite was added (5-10 eq, 0.5-1.0 mmol). The vial was capped using an ElectraSyn vial cap that is fitted with a septa and two electrodes (working and counter electrodes) and the solution was bubbled with nitrogen. A balloon with nitrogen was then placed on the septa. The electrochemical set-up was attached to the ElectraSyn 2.0 and electrolysis was conducted at a current of 10 mA for 4 F/mol with stirring.

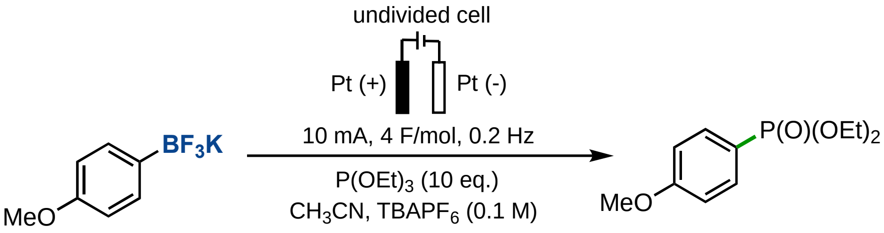

Variations	Yield (^{31}P NMR vs Ph_3PO as internal standard)
none	45%
$LiPF_6$	trace
$NaPF_6$	trace
$TEAPF_6$	48%
TBAI	2%
TBAOAc	24%
$TBAClO_4$	43%
$TBANO_3$	48%
$TBAHSO_4$	48%
$TBABF_4$	62%
$TBABF_4$, H_2O	trace
$TBABF_4$, MeOH	trace
$TBABF_4$, acetone	69%
$TBABF_4$, acetone, 5 mA	37%
$TBABF_4$, acetone, 20 mA	54%
$TBABF_4$, acetone, 2 F/mol	37%
$TBABF_4$, acetone, 8 F/mol	69%
No electrolyte, acetone	73%
No electrolyte, acetone, [C]x2	87%
No electrolyte, dry acetone, [C]x2	90%
$B(OH)_2$ instead of BF_3K	23%
Potassium 1,1,1-tris(hydroxymethyl)ethane borate instead of BF_3K	14%

Table S2. Optimization of the reaction using pulsed electrolysis (alternating polarity at 0.2 Hz).

Ultimately, it was found that removing the electrolyte was beneficial to the reaction. Doubling the concentration of reactants, and use of acetone as a solvent was found to be the best compromise to obtain appreciable yields.

4. X-Ray Photoelectron Spectroscopy (XPS) analysis of the electrodes

XPS measurements were recorded on a Nexsa G2 instrument from Thermo Fisher Scientific. The electrodes were stored under vacuum overnight before analysis, then mounted on the sample holder with copper tape. Analyses were recorded under ultra-high vacuum at 3.0E-09 mBar. The X-ray source used is a monochromated, micro-focused, low-power Al K-Alpha source. Due to high thickness of the passivation layer on the electrode without alternating polarity, analysis was recorded with a flood gun (0.1 eV).

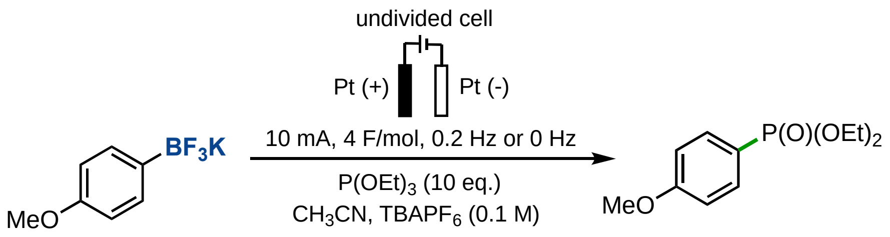

1

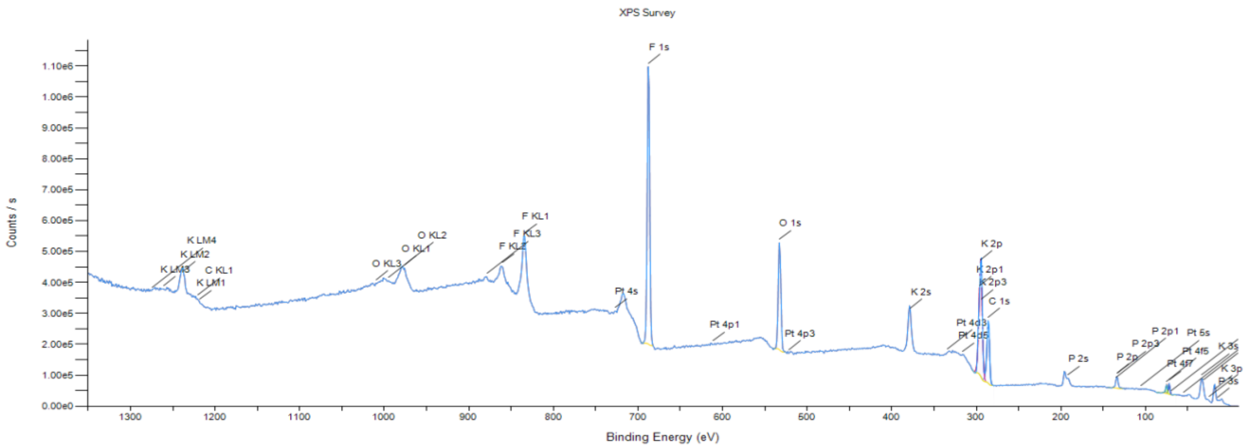

Figure S4b. XPS survey of a platinum electrode used in CCE

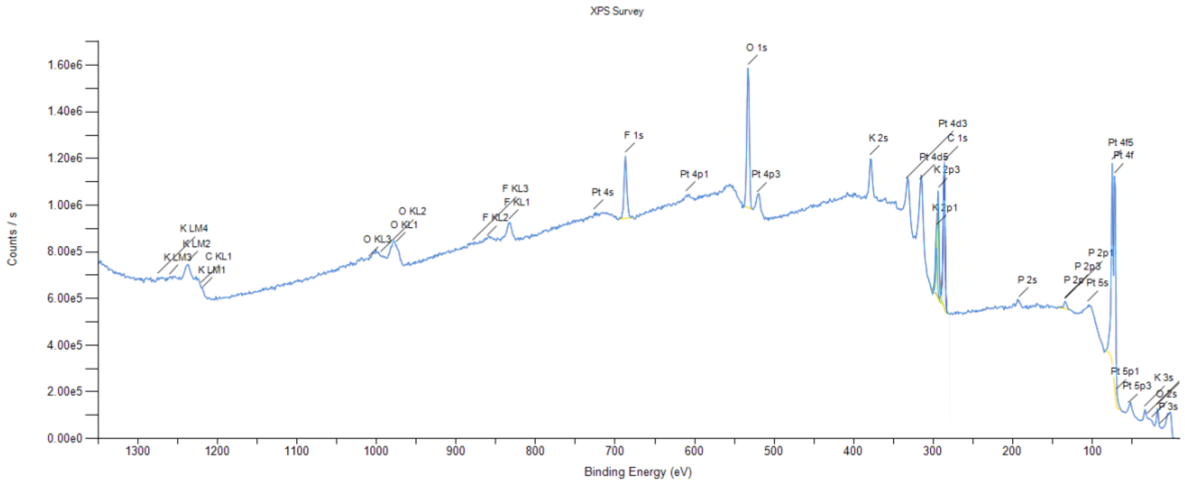

Figure S4c. XPS survey of a platinum electrode used in alternating polarity electrolysis.

The amount of exposed platinum atoms was quantified for four different electrodes: an electrode used in constant current electrolysis, an electrode used in alternating polarity electrolysis, an electrode dipped in the reaction media but without electrolysis, and a never used electrode. They were vigorously washed with acetone and air-dried before analysis. In addition, the surface composition of the electrodes with and without alternating polarity can be compared. The amount of fluorine is way higher in the case where no alternating polarity was used.

Electrode	Atomic Pt%
Blank	4.3
Dipped	4.68
CCE	0.26
AP	6.44

Table S3. The amount of exposed platinum atoms for a brand new electrode, an electrode dipped in the reaction media but without electrolysis, an electrode used in constant current electrolysis, an electrode used in alternating polarity electrolysis.

Name	No AP Atomic %	AP Atomic %
Pt 4f7	0.26	6.44
P 2p	3.21	2.47
C 1s	28.68	48.83
K 2p	13.55	10.61
O 1s	18.54	22.68
F 1s	35.75	8.97

Table S4. Comparison of surface compositions of the electrodes with and without alternating polarity.

5. Radical trap experiments

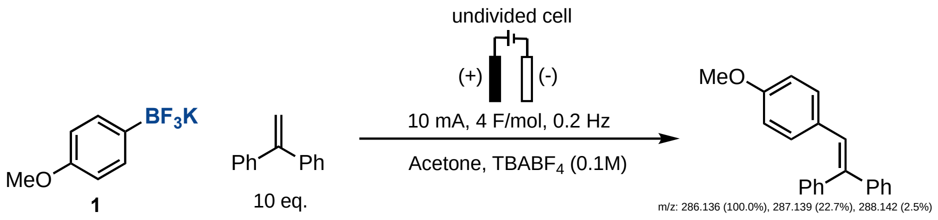

In an IKA Electrasyn vial equipped with two platinum plates (10 x 10 x 0.1 mm, wired with a platinum wire), potassium 4-methoxyphenyltrifluoroborate (43 mg, 0.2 mmol, 1 eq.) was dissolved in acetone (4 mL, $TBABF_4$ 0.1M). 1,1-diphenylethylene (360 mg, 2 mmol, 10 eq.) was added. The reaction mixture was sparged with nitrogen for 5 minutes, then a balloon of nitrogen was added to keep the vial under a positive pressure of nitrogen. The reaction was electrolyzed at 10 mA for 4 F/mol, an alternative polarity frequency of 0.2 Hz was applied. The reaction crude mixture was analyzed by GC/MS (Figure S5).

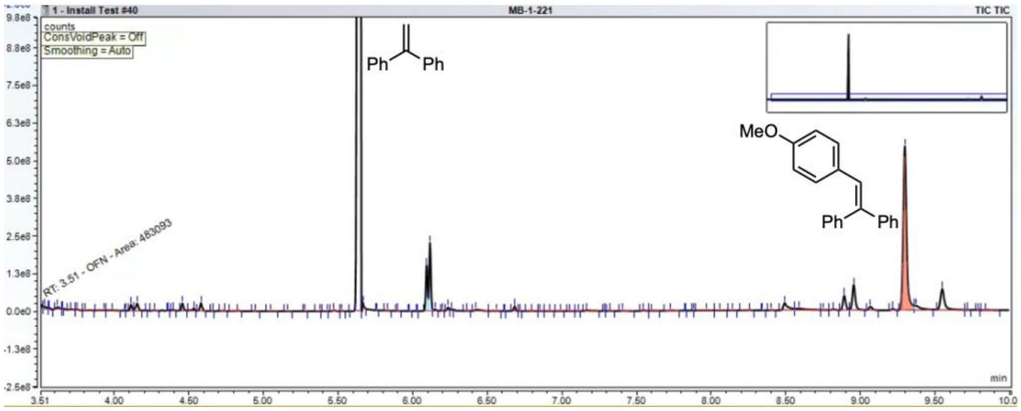

Figure S5. GC/MS Chromatogram of crude mixture.

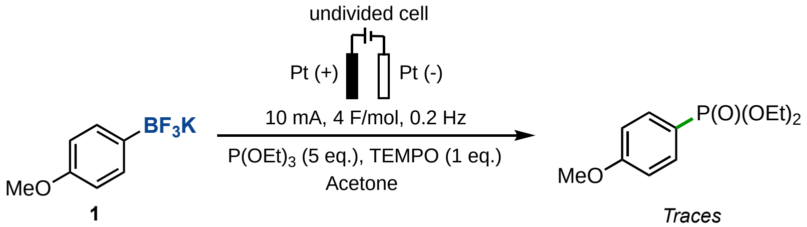

In an IKA Electrasyn vial equipped with two platinum plates (10 x 10 x 0.1 mm, wired with a platinum wire), potassium 4-methoxyphenyltrifluoroborate (43 mg, 0.2 mmol, 1 eq.) was dissolved in acetone (4 mL). $P(OEt)_3$ (166 mg, 1 mmol, 10 eq.) and TEMPO (31 mg, 0.2 mmol, 1eq.) were added. The reaction mixture was sparged with nitrogen for 5 minutes, then a balloon of nitrogen was added to keep the vial under a positive pressure of nitrogen. The reaction was electrolyzed at 10 mA for 4 F/mol, an alternative polarity frequency of 0.2 Hz was applied. Crude analysis of the mixture via GC/MS only showed traces of expected product and TEMPO adduct.

6. Substrate scope

General note : Isolation appeared to be relatively tedious, products often co-eluting with decomposition by-products of $P(OEt)_3$. In some case, the conditions were slightly improved for some substrates (current increased if low conversion or passing more electrons). The frequency could vary from 0.1 to 0.5 Hz. GC/MS confirmed the presence of each product in the crude mixture, as the sole molecule containing an arene.

Diethyl *P*-(4-methoxyphenyl)phosphonate (**2**)

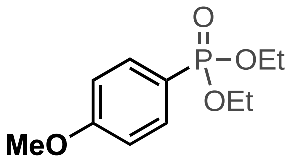

In an IKA Electrasyn vial equipped with two platinum plates (10 x 10 x 0.1 mm, wired with a platinum wire), potassium 4-methoxyphenyltrifluoroborate (43 mg, 0.2 mmol, 1 eq.) was dissolved in acetone (4 mL). $P(OEt)_3$ (166 mg, 1 mmol, 5 eq.) was added. The reaction mixture was sparged with nitrogen for 5 minutes, then a balloon of nitrogen was added to keep the vial under a positive pressure of nitrogen. The reaction was electrolyzed at 10 mA for 4 F/mol, an alternative polarity frequency of 0.2 Hz was applied. At the end of the reaction the mixture was concentrated, dissolved with dichloromethane (10 mL) and washed with water. The organic phase was then concentrated under vacuum and purified via column chromatography (ethyl acetate/hexane gradient, 10–50%) and compound **2** was obtained as a colorless oil (36 mg, 74% yield). The NMR data matches literature.[1]

^{1}H NMR (499.8 MHz, $CDCl_3$) δ 7.71 (dd, *J* = 12.8, 8.9 Hz, 2H), 6.93 (dd, *J* = 8.8, 3.4 Hz, 2H), 4.14 – 3.96 (m, 4H), 3.82 (s, 3H), 1.28 (t, *J* = 7.1 Hz, 6H).

^{31}P NMR (202.3 MHz, $CDCl_3$) δ 19.72 (m).

Diethyl *P*-(4-phenoxyphenyl)phosphonate (**3**)

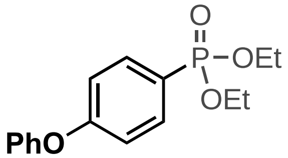

In an IKA Electrasyn vial equipped with two platinum plates (10 x 10 x 0.1 mm, wired with a platinum wire), potassium 4-methoxyphenyltrifluoroborate (43 mg, 0.2 mmol, 1 eq.) was dissolved in acetone (4 mL). $P(OEt)_3$ (166 mg, 1 mmol, 5 eq.) was added. The reaction mixture was sparged with nitrogen for 5 minutes, then a balloon of nitrogen was added to keep the vial under a positive pressure of nitrogen. The reaction was electrolyzed at 10 mA for 4 F/mol, an alternative polarity frequency of 0.5 Hz was applied. At the end of the reaction the mixture was concentrated, dissolved with dichloromethane (10 mL) and washed with water. The organic phase was then concentrated under vacuum and purified via column chromatography (ethyl acetate/hexane gradient, 10–50%) and compound **3** was obtained as a colorless oil (33 mg, 54% yield). The NMR data matches literature.[2]

^{1}H NMR (499.8 MHz, $CDCl_3$) δ 7.75 (dd, *J* = 12.8, 8.7 Hz, 2H), 7.38 (d, *J* = 8.6 Hz, 2H), 7.17 (t, *J* = 7.5 Hz, 1H), 7.05 (d, *J* = 7.5 Hz, 2H), 7.01 (dd, *J* = 8.7, 3.5 Hz, 2H), 4.19 – 3.99 (m, 4H), 1.31 (t, *J* = 7.1 Hz, 6H).

Diethyl *P*-(*p*-tolyl)phosphonate (**4**)

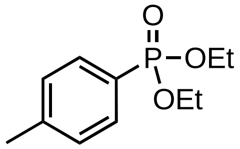

In an IKA Electrasyn vial equipped with two platinum plates (10 x 10 x 0.1 mm, wired with a platinum wire), potassium 4-(*p*-tolyl)trifluoroborate (40 mg, 0.2 mmol, 1 eq.) was dissolved in acetone (4 mL). $P(OEt)_3$ (166 mg, 1 mmol, 5 eq.) was added. The reaction mixture was sparged with nitrogen for 5 minutes, then a balloon of nitrogen was added to keep the vial under a positive pressure of nitrogen. The reaction was electrolyzed at 20 mA for 4 F/mol, an alternative polarity frequency of 0.5 Hz was applied. At the end of the reaction the mixture was concentrated, dissolved with dichloromethane (10 mL) and washed with water. The organic phase was then concentrated under vacuum and the yield was determined vs triphenylphosphine oxide as an internal standard (72%). The NMR data matches literature.[1]

^{31}P NMR (202.3 MHz, $CDCl_3$) δ 19.60 (m)

Diethyl *P*-(*o*-tolyl)phosphonate (**5**)

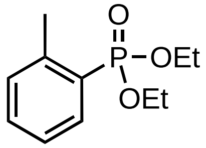

In an IKA Electrasyn vial equipped with two platinum plates (10 x 10 x 0.1 mm, wired with a platinum wire), potassium 4-(*p*-tolyl)trifluoroborate (40 mg, 0.2 mmol, 1 eq.) was dissolved in acetone (4 mL). $P(OEt)_3$ (166 mg, 1 mmol, 5 eq.) was added. The reaction mixture was sparged with nitrogen for 5 minutes, then a balloon of nitrogen was added to keep the vial under a positive pressure of nitrogen. The reaction was electrolyzed at 20 mA for 4 F/mol, an alternative polarity frequency of 0.5 Hz was applied. At the end of the reaction the mixture was concentrated, dissolved with dichloromethane (10 mL) and washed with water. The organic phase was then concentrated under vacuum and the yield was determined vs triphenylphosphine oxide as an internal standard (52%). The NMR data matches literature.[1]

^{31}P NMR (202.3 MHz, $CDCl_3$) δ 19.44 (m)

Diethyl *P*-(4-fluorophenyl)phosphonate (**6**)

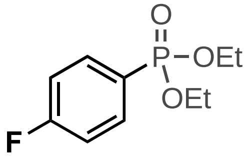

In an IKA Electrasyn vial equipped with two platinum plates (10 x 10 x 0.1 mm, wired with a platinum wire), potassium 4-fluorophenyltrifluoroborate (43 mg, 0.2 mmol, 1 eq.) was dissolved in acetone (4 mL). $P(OEt)_3$

(166 mg, 1 mmol, 5 eq.) was added. The reaction mixture was sparged with nitrogen for 5 minutes, then a balloon of nitrogen was added to keep the vial under a positive pressure of nitrogen. The reaction was electrolyzed at 20 mA for 4 F/mol, an alternative polarity frequency of 0.5 Hz was applied. At the end of the reaction the mixture was concentrated, dissolved with dichloromethane (10 mL) and washed with water. The organic phase was then concentrated under vacuum and the yield was determined vs triphenylphosphine oxide as an internal standard (57%). The compound appeared the be relatively volatile and impossible to separate from phosphorus containing impurities. The NMR data matches literature.[3]

^{31}P NMR (202.3 MHz, $CDCl_3$) δ 17.35 (m).

^{19}F NMR (470.3 MHz, $CDCl_3$) δ -107.64 (m).

Diethyl *P*-(4-(trifluoromethyl)phenyl)phosphonate (**7**)

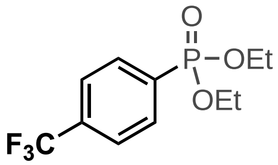

In an IKA Electrasyn vial equipped with two platinum plates (10 x 10 x 0.1 mm, wired with a platinum wire), potassium 4-trlfluoromethylphenyltrifluoroborate (43 mg, 0.2 mmol, 1 eq.) was dissolved in acetone (4 mL). $P(OEt)_3$ (166 mg, 1 mmol, 5 eq.) was added. The reaction mixture was sparged with nitrogen for 5 minutes, then a balloon of nitrogen was added to keep the vial under a positive pressure of nitrogen. The reaction was electrolyzed at 20 mA for 4 F/mol, an alternative polarity frequency of 0.5 Hz was applied. At the end of the reaction the mixture was concentrated, dissolved with dichloromethane (10 mL) and washed with water. The organic phase was then concentrated under vacuum and the yield was determined vs triphenylphosphine oxide as an internal standard (32%). Purification was attempted via column chromatograph (ethyl acetate/hexane gradient, 10–50%) and obtained as a colorless oil, the product co-elutes with 20% of an unknown impurity containing a phosphorus and a trifluoromethyl. GC/MS did not show any other product). The NMR data matches literature.[4]

^{1}H NMR (499.8 MHz, $CDCl_3$) δ 7.88-7.94 (m, 2H), 7.70 (m 2H), 4.22 – 4.04 (m, 4H), 1.31 (t, *J* = 7.1 Hz, 6H).

^{31}P NMR (202.3 MHz, $CDCl_3$) δ 16.22 (m).

^{19}F NMR (470.3 MHz, $CDCl_3$) δ -63.32 (m).

Diethyl *P*-(4-bromophenyl)phosphonate (**8**)

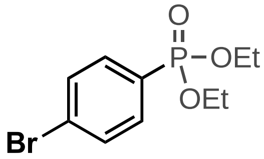

In an IKA Electrasyn vial equipped with two platinum plates (10 x 10 x 0.1 mm, wired with a platinum wire), Potassium 4-bromophenyl trifluoroborate (53 mg, 0.2 mmol, 1 eq.) was dissolved in acetone (4 mL). $P(OEt)_3$

(166 mg, 1 mmol, 5 eq.) and $TBABF_4$ (0.1 M) were added. The reaction mixture was sparged with nitrogen for 5 minutes, then a balloon of nitrogen was added to keep the vial under a positive pressure of nitrogen. The reaction was electrolyzed at 20 mA for 4 F/mol, an alternative polarity frequency of 0.2 Hz was applied. At the end of the reaction the mixture was concentrated, dissolved with dichloromethane (10 mL) and washed with water. The organic phase was then concentrated under vacuum and the yield was determined vs 1,3,5-trimethoxybenzene as an internal standard (17%). The NMR data matches literature.[3]

^{1}H NMR (499.8 MHz, $CDCl_3$) δ 7.71 (dd, *J* = 13.0, 8.6 Hz, 2H), 7.41 (dd, *J* = 8.6, 3.4 Hz, 2H), 4.17 – 3.91 (m, 4H), 1.28 (t, *J* = 7.0 Hz, 6H).

^{31}P NMR (202.3 MHz, $CDCl_3$) δ 17.56 (m).

Diethyl *P*-(4-chlorophenyl)phosphonate (**9**)

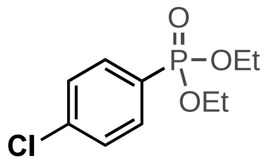

In an IKA Electrasyn vial equipped with two platinum plates (10 x 10 x 0.1 mm, wired with a platinum wire), Potassium 4-chlorophenyl trifluoroborate (44 mg, 0.2 mmol, 1 eq.) was dissolved in acetone (4 mL). $P(OEt)_3$ (166 mg, 1 mmol, 5 eq.) and $TBABF_4$ (0.1 M) were added. The reaction mixture was sparged with nitrogen for 5 minutes, then a balloon of nitrogen was added to keep the vial under a positive pressure of nitrogen. The reaction was electrolyzed at 20 mA for 4 F/mol, an alternative polarity frequency of 0.2 Hz was applied. At the end of the reaction, the mixture was washed with water and concentrated. Compound 9 was purified via column chromatography (ethyl acetate/hexane gradient, 0–30%) and obtained as a colorless oil (34 mg, 68% yield). The NMR data matches literature.[3]

^{1}H NMR (499.8 MHz, $CDCl_3$) δ 7.71 (dd, *J* = 13.0, 8.6 Hz, 2H), 7.41 (dd, *J* = 8.6, 3.4 Hz, 2H), 4.17 – 3.91 (m, 4H), 1.28 (t, *J* = 7.0 Hz, 6H).

^{31}P NMR (202.3 MHz, $CDCl_3$) δ 17.56 (m).

Diethyl *P*-(4-isopropyl-3,5-methylphenyl)phosphonate (**10**)

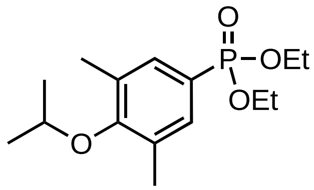

In an IKA Electrasyn vial equipped with two platinum plates (10 x 10 x 0.1 mm, wired with a platinum wire), Potassium 4-chlorophenyl trifluoroborate (54 mg, 0.2 mmol, 1 eq.) was dissolved in acetone (4 mL). $P(OEt)_3$ (166 mg, 1 mmol, 5 eq.) and $TBABF_4$ (0.1 M) were added. The reaction mixture was sparged with nitrogen for 5 minutes, then a balloon of nitrogen was added to keep the vial under a positive pressure of nitrogen. The reaction was electrolyzed at 10 mA for 4 F/mol, an alternative polarity frequency of 0.2 Hz was applied.

At the end of the reaction, the mixture was washed with water and concentrated. Compound **10** was purified via column chromatography (ethyl acetate/hexane gradient, 10–90%) and obtained as a colorless oil (52 mg, 90% yield).

^{1}H NMR (499.8 MHz, $CDCl_3$) δ 7.39 (s, 1H), 7.37 (s, 1H), 4.17 (p, *J* = 6.1 Hz, 1H), 4.12 – 3.92 (m, 4H), 2.21 (s, 6H), 1.27 – 1.21 (m, 12H).

^{31}P NMR (202.3 MHz, $CDCl_3$) δ 17.85 (m).

^{13}C NMR (125.7 MHz, $CDCl_3$) δ 157.58 (d, *J* = 3.9 Hz), 131.57, 131.48, 130.88, 130.75, 121.16 (d, *J* = 190.5 Hz), 73.87, 60.93, 60.88, 21.55 (2C), 16.12 (2C), 15.37, 15.32.

Diethyl *P*-(4-methoxy-3-fluorophenyl)phosphonate (**11**)

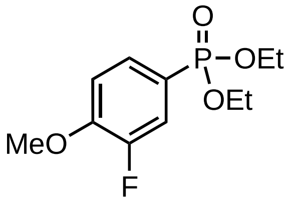

In an IKA Electrasyn vial equipped with two platinum plates (10 x 10 x 0.1 mm, wired with a platinum wire), Potassium 4-chlorophenyl trifluoroborate (46 mg, 0.2 mmol, 1 eq.) was dissolved in acetone (4 mL). $P(OEt)_3$ (166 mg, 1 mmol, 5 eq.) and $TBABF_4$ (0.1 M) were added. The reaction mixture was sparged with nitrogen for 5 minutes, then a balloon of nitrogen was added to keep the vial under a positive pressure of nitrogen. The reaction was electrolyzed at 20 mA for 4 F/mol, an alternative polarity frequency of 0.2 Hz was applied. At the end of the reaction, the mixture was washed with water and concentrated. Compound **11** was purified via column chromatography (ethyl acetate/hexane gradient, 10–80%) and obtained as a colorless oil (40 mg, 80% yield). The NMR data matches literature.[5]

^{1}H NMR (499.8 MHz, $CDCl_3$) δ 7.57 (ddt, *J* = 13.2, 8.3, 1.5 Hz, 1H), 7.51 – 7.43 (m, 1H), 7.02 (td, *J* = 8.1, 4.3 Hz, 1H), 4.17 – 4.00 (m, 4H), 3.92 (s, 3H), 1.31 (t, *J* = 7.1 Hz, 6H).

^{31}P NMR (202.3 MHz, $CDCl_3$) δ 17.64 (m).

Diethyl *P*-(1-naphthalenyl)phosphonate (**12**)

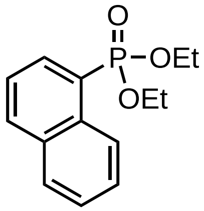

In an IKA Electrasyn vial equipped with two platinum plates (10 x 10 x 0.1 mm, wired with a platinum wire), Potassium 1-naphthalenyl trifluoroborate (47 mg, 0.2 mmol, 1 eq.) was dissolved in acetone (4 mL). $P(OEt)_3$ (166 mg, 1 mmol, 5 eq.) and $TBABF_4$ (0.1 M) were added. The reaction mixture was sparged with nitrogen for 5 minutes, then a balloon of nitrogen was added to keep the vial under a positive pressure of nitrogen.

The reaction was electrolyzed at 20 mA for 4 F/mol, an alternative polarity frequency of 0.2 Hz was applied. At the end of the reaction, the mixture was washed with water and concentrated. Compound **12** was purified via column chromatography (ethyl acetate/hexane gradient, 0–30%) and obtained as a colorless oil (20 mg, 41% yield). The NMR data matches literature.[6]

^{1}H NMR (499.8 MHz, CDCl3) δ 8.51 (d, *J* = 8.4 Hz, 1H), 8.24 (dd, *J* = 15.6, 7.8 Hz, 1H), 8.03 (d, *J* = 8.2 Hz, 1H), 7.89 (d, *J* = 8.2 Hz, 1H), 7.65 – 7.57 (m, 1H), 7.57 – 7.44 (m, 2H), 4.29 – 4.16 (m, 2H), 4.10 – 4.02 (m, 2H), 1.30 (t, *J* = 7.1 Hz, 6H).

^{31}P NMR (202.3 MHz, CDCl3) δ 19.17 (m)

Diethyl *P*-(1-(4-methyl)naphthalenyl)phosphonate (**13**)

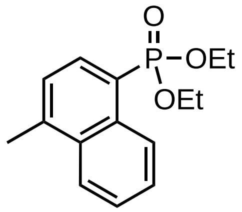

In an IKA Electrasyn vial equipped with two platinum plates (10 x 10 x 0.1 mm, wired with a platinum wire), Potassium 4-methyl-1-naphtenyl-trifluoroborate (50 mg, 0.2 mmol, 1 eq.) was dissolved in acetone (4 mL). $P(OEt)_3$ (166 mg, 1 mmol, 5 eq.) was added. The reaction mixture was sparged with nitrogen for 5 minutes, then a balloon of nitrogen was added to keep the vial under a positive pressure of nitrogen. The reaction was electrolyzed at 20 mA for 4 F/mol, an alternative polarity frequency of 0.5 Hz was applied. At the end of the reaction, the mixture was concentrated and directly purified via column chromatography (ethyl acetate/hexane gradient, 0–30%) and compound **13** was obtained as a colorless oil (40 mg, 71% yield). The NMR data matches literature.[7]

^{1}H NMR (499.8 MHz, $CDCl_3$) δ 8.59 – 8.49 (m, 1H), 8.15 (dd, *J* = 16.4, 7.2 Hz, 1H), 8.07 (dt, *J* = 7.5, 2.4 Hz, 1H), 7.60 (pd, *J* = 6.8, 1.7 Hz, 2H), 7.38 (dd, *J* = 7.2, 3.7 Hz, 1H), 4.24 – 4.01 (m, 4H), 2.74 (s, 3H), 1.30 (t, *J* = 7.0 Hz, 6H).

^{31}P NMR (202.3 MHz, $CDCl_3$) δ 19.81 (m).

Diethyl *P*-(3-Fluoro[1,1′-biphenyl]-4-yl)phosphonate (**14**)

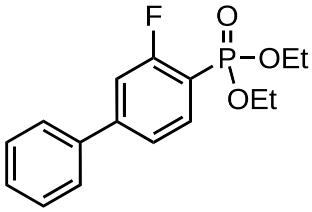

In an IKA Electrasyn vial equipped with two platinum plates (10 x 10 x 0.1 mm, wired with a platinum wire), Potassium 3-fluoro[1,1'-biphenyl]-4-yl-trifluoroborate (56 mg, 0.2 mmol, 1 eq.) was dissolved in acetone (4 mL). $P(OEt)_3$ (166 mg, 1 mmol, 5 eq.) was added. The reaction mixture was sparged with nitrogen for 5 minutes, then a balloon of nitrogen was added to keep the vial under a positive pressure of nitrogen. The

reaction was electrolyzed at 20 mA for 4 F/mol, an alternative polarity frequency of 0.5 Hz was applied. At the end of the reaction, the mixture was concentrated and directly purified via column chromatography (ethyl acetate/hexane gradient, 0–30%) and compound **14** was obtained as a colorless oil (38 mg, 61% yield).

^{1}H NMR (499.8 MHz, $CDCl_3$) δ 7.68-7.39 (m, 8H), 4.23 – 4.08 (m, 4H), 2.74 (s, 3H), 1.36 (t, *J* = 7.0 Hz, 6H).

^{31}P NMR (202.3 MHz, $CDCl_3$) δ 16.66 (m).

^{13}C NMR (125.7 MHz, $CDCl_3$) δ 159.51 (dd, *J* = 251.2, 21.5 Hz), 133.37 (dd, *J* = 13.5, 3.2 Hz), 131.27 (dd, *J* = 17.3, 3.4 Hz), 130.39 (d, *J* = 6.6 Hz), 129.15 (d, *J* = 2.9 Hz), 128.87 (d, *J* = 6.6 Hz), 128.74, 128.58, 127.85 (dd, *J* = 9.1, 3.9 Hz), 62.57 (d, *J* = 5.5 Hz), 16.49 (d, *J* = 6.5 Hz).

HRMS (ES-MS$^+$): exact mass (monoisotopic) calcd. For [M+H$^+$] ($C_{16}H_{19}FO_3P$): 309.1050; found, 309.1068.

Diethyl *P*-(2-benzo[*b*]thiophene)phosphonate (**15**)

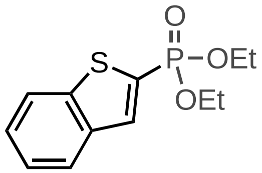

In an IKA Electrasyn vial equipped with two platinum plates (10 x 10 x 0.1 mm, wired with a platinum wire), Potassium benzofuran-2-trifluoroborate (48 mg, 0.2 mmol, 1 eq.) was dissolved in acetone (4 mL). $P(OEt)_3$ (166 mg, 1 mmol, 5 eq.) was added. The reaction mixture was sparged with nitrogen for 5 minutes, then a balloon of nitrogen was added to keep the vial under a positive pressure of nitrogen. The reaction was electrolyzed at 5 mA for 4 F/mol, an alternative polarity frequency of 0.2 Hz was applied. At the end of the reaction, the mixture was concentrated. Compound **15** was purified via column chromatography (ethyl acetate/hexane gradient, 10–50%) and obtained as a colorless oil (30 mg, 56% yield). The NMR data matches literaterature.[8]

^{1}H NMR (499.8 MHz, $CDCl_3$) δ 7.93 (d, *J* = 9.3Hz, 1H), 7.89 (d, *J* = 7.9 Hz, 2H), 7.42 (qd, *J* = 7.6 Hz, *J* = 1.7 Hz, 2H), 4.25-4.10 (m, 4H), 1.35 (t, J = 7.1 Hz, 6H).

^{31}P NMR (202.3 MHz, $CDCl_3$) δ 11.70 (m).

Diethyl *P*-(benzofuran-2-yl)phosphonate (**16**)

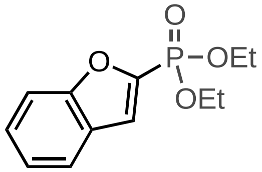

In an IKA Electrasyn vial equipped with two platinum plates (10 x 10 x 0.1 mm, wired with a platinum wire), potassium benzofuran-2-yltrifluoroborate (45 mg, 0.2 mmol, 1 eq.) was dissolved in acetone (4 mL). $P(OEt)_3$ (166 mg, 1 mmol, 5 eq.) and $TBABF_4$ (0.1 M) were added. The reaction mixture was sparged with nitrogen for 5 minutes, then a balloon of nitrogen was added to keep the vial under a positive pressure of nitrogen.

The reaction was electrolyzed at 10 mA for 4 F/mol, an alternative polarity frequency of 0.1 Hz was applied. At the end of the reaction, the mixture was washed with water and concentrated. Compound **16** was purified via column chromatography (ethyl acetate/hexane gradient, 10–50%) and obtained as a colorless oil (47 mg, 94% yield). The NMR data matches literature.[6]

^{1}H NMR (499.8 MHz, $CDCl_3$) δ 7.67 (d, *J* = 7.8 Hz, 1H), 7.56 (d, *J* = 8.4 Hz, 1H), 7.49 (dd, *J* = 2.7 Hz, *J* = 0.8 Hz, 1H), 7.41 (t, *J* = 7.8 Hz, 1H), 7.29 (td *app*, *J* = 7.8 Hz, *J* = 0.8 Hz, 1H), 4.29-4.14 (m, 4H), 1.36 (t, J = 7.0 Hz, 6H).

^{31}P NMR (202.3 MHz, $CDCl_3$) δ 5.13 (m).

Diethyl *P*-(5-quinolinyl)phosphonate (**17**)

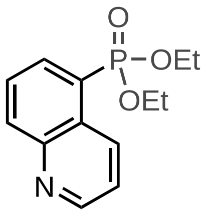

In an IKA Electrasyn vial equipped with two platinum plates (10 x 10 x 0.1 mm, wired with a platinum wire), potassium 5-quinoline trifluoroborate (47 mg, 0.2 mmol, 1 eq.) was dissolved in 1,1,1,3,3,3-hexafluoro-2-propanol (4 mL). $P(OEt)_3$ (166 mg, 1 mmol, 5 eq.) was added. The reaction mixture was sparged with nitrogen for 5 minutes, then a balloon of nitrogen was added to keep the vial under a positive pressure of nitrogen. The reaction was electrolyzed at 5 mA for 8 F/mol, an alternative polarity frequency of 0.1 Hz was applied. At the end of the reaction, the mixture was concentrated. Compound **17** was purified via column chromatography (ethyl acetate/hexane gradient, 20–100%) and obtained as a colorless oil (35 mg, 67% yield).

^{1}H NMR (499.8 MHz, $CDCl_3$) δ 8.98 (dd, *J* = 4.2 Hz, 1.7 Hz, 1H), 8.90 (d, *J* = 8.7 Hz, 1H), 8.30 (d, *J* = 8.7 Hz, 1H), 8.26 (ddd, *J* = 15.9 Hz, 7.1 Hz, 1.4 Hz, 1H), 7.77 (ddd, *J* = 8.7 Hz, *J* = 7.1 Hz, *J* = 4.2 Hz, 1H), 7.52 (dd, J = 8.7, 4.2 Hz, 1H), 4.19-4.06 (m, 4H), 1.32 (t, J = 7.1 Hz, 6H).

^{13}C NMR (125.7 MHz, $CDCl_3$) δ 150.91, 148.37 (d, *J* = 12.7 Hz), 135.14 (d, *J* = 7.8 Hz), 135.11 (d, *J* = 7.2 Hz), 134.77 (d, *J* = 8.9 Hz), 128.39 (d, *J* = 10.4 Hz), 128.33 (d, *J* = 17.7 Hz), 125.71 (d, *J* = 183.2 Hz), 122.25, 16.47.

^{31}P NMR (202.3 MHz, $CDCl_3$) δ 16.80 (m).

HRMS (ES-MS$^+$): exact mass (monoisotopic) calcd. For [M+H$^+$] ($C_{16}H_{19}FO_3P$): 266.0944; found, 266.0944

Diethyl *P*-(2,6-Dimethoxy-3-pyridinyl)phosphonate (**18**)

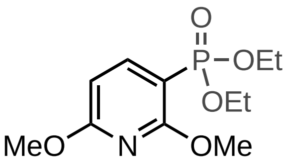

In an IKA Electrasyn vial equipped with two platinum plates (10 x 10 x 0.1 mm, wired with a platinum wire), potassium 2,6-dimethoxypyridine-3-trifluoroborate (49 mg, 0.2 mmol, 1 eq.) was dissolved in acetone (4 mL). $P(OEt)_3$ (166 mg, 1 mmol, 5 eq.) was added. The reaction mixture was sparged with nitrogen for 5 minutes, then a balloon of nitrogen was added to keep the vial under a positive pressure of nitrogen. The reaction was electrolyzed at 10 mA for 4 F/mol, an alternative polarity frequency of 0.1 Hz was applied. At the end of the reaction, the mixture was concentrated. The compound was purified via column chromatography (ethyl acetate/hexane gradient, 10–60%) and obtained as a colorless oil (38 mg, 70% yield). The NMR data matches literature.[9]

^{1}H NMR (499.8 MHz, $CDCl_3$) δ 7.98 (dd, *J* = 13.2 Hz, *J* = 8.5 Hz, 1H), 6.34 (dd, *J* = 8.5 Hz, *J* = 1.7 Hz, 1H), 4.19-4.06 (m, 4H), 4.01 (s, 3H), 3.95 (s, 3H), 1.32 (t, J = 7.1 Hz, 6H).

^{31}P NMR (202.3 MHz, $CDCl_3$) δ 16.80 (m).

Diethyl *P*-(2,3-dihydro-1,4-benzodioxin-6-yl)phosphonate (**19**)

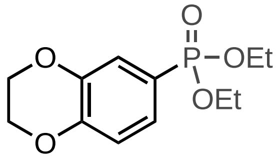

In an IKA Electrasyn vial equipped with two platinum plates (10 x 10 x 0.1 mm, wired with a platinum wire), Potassium 1,4-benzodioxane-6-trifluoroborate (48 mg, 0.2 mmol, 1 eq.) was dissolved in acetone (4 mL). $P(OEt)_3$ (166 mg, 1 mmol, 5 eq.) and $TBABF_4$ (0.1 M) were added. The reaction mixture was sparged with nitrogen for 5 minutes, then a balloon of nitrogen was added to keep the vial under a positive pressure of nitrogen. The reaction was electrolyzed at 10 mA for 4 F/mol, an alternative polarity frequency of 0.1 Hz was applied. At the end of the reaction, the mixture was washed with water and concentrated. Compound was purified via column chromatography (ethyl acetate/hexane gradient, 0–50%) and product was obtained as a colorless oil (34 mg, 64% yield). The NMR data matches literature.[10]

^{1}H NMR (499.8 MHz, $CDCl_3$) δ 7.30-7.24 (m, 2H), 6.91 (dd, *J* = 8.2, 4.5 Hz, 1H), 4.26 (m, 4H), 4.06 (m, 4H), 1.29 (t, *J* = 7.1 Hz, 6H).

^{31}P NMR (202.3 MHz, $CDCl_3$) δ 19.10 (m).

Diethyl *P*-(dibenzofuran-4-yl)phosphonate (**20**)

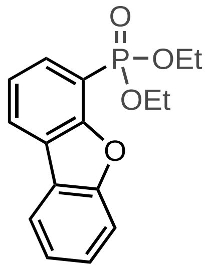

In an IKA Electrasyn vial equipped with two platinum plates (10 x 10 x 0.1 mm, wired with a platinum wire), potassium 4-dibenzofuranyltrifluoroborate (43 mg, 0.2 mmol, 1 eq.) was dissolved in acetone (4 mL). $P(OEt)_3$ (166 mg, 1 mmol, 5 eq.) was added. The reaction mixture was sparged with nitrogen for 5 minutes, then a balloon of nitrogen was added to keep the vial under a positive pressure of nitrogen. The reaction was electrolyzed at 10 mA for 4 F/mol, an alternative polarity frequency of 0.5 Hz was applied. At the end of the reaction the mixture was concentrated, dissolved with dichloromethane (10 mL) and washed with water. The organic phase was then concentrated under vacuum and purified via column chromatography (ethyl acetate/hexane gradient, 10–50%) and product was obtained as a colorless oil (24 mg, 40% yield). The NMR data matches literature.[11]

^{1}H NMR (499.8 MHz, $CDCl_3$) δ 8.14 (d, *J* = 7.7 Hz, 1H), 7.96 (d, *J* = 7.5 Hz, 1H), 7.93 (dd, *J* = 7.5, 1.4 Hz, 1H), 7.68 (d, *J* = 8.3 Hz, 1H), 7.50 (t, *J* = 7.2 Hz, 1H), 7.43 (td, *J* = 7.6, 2.9 Hz, 1H), 7.38 (t, *J* = 8.0 Hz, 1H), 4.31 – 4.16 (m, 4H), 1.36 (t, *J* = 7.1 Hz, 6H).

^{31}P NMR (202.3 MHz, $CDCl_3$) δ 14.55 (m).

1-methoxy-4-(phenylseleno)benzene (**21**)

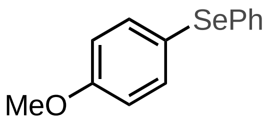

In an IKA Electrasyn vial equipped with two platinum plates (10 x 10 x 0.1 mm, wired with a platinum wire), Potassium 4-methoxybenzene-trifluoroborate (43 mg, 0.2 mmol, 1 eq.) was dissolved in acetone (4 mL). Diphenyldiselenide (312 mg, 1 mmol, 5 eq.) was added. The reaction mixture was sparged with nitrogen for 5 minutes, then a balloon of nitrogen was added to keep the vial under a positive pressure of nitrogen. The reaction was electrolyzed at 10 mA for 4 F/mol, an alternative polarity frequency of 0.5 Hz was applied. At the end of the reaction, the mixture was concentrated and directly purified via column chromatography (ethyl acetate/hexane gradient, 0–10%). Compound **21** was obtained as a colorless oil (43 mg, 82% yield). The NMR data matches literature.[12]

^{1}H NMR (499.8 MHz, $CDCl_3$) δ 7.50 (d, *J* = 8.8 Hz, 2H), 7.35 – 7.30 (m, 2H), 7.26 – 7.14 (m, 3H), 6.85 (d, *J* = 8.9 Hz, 2H), 3.79 (s, 3H).

^{77}Se NMR (189.5 MHz, $CDCl_3$) δ 400.63 (s).

1-methoxy-4-(phenyltelluro)benzene (**22**)

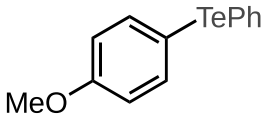

In an IKA Electrasyn vial equipped with two platinum plates (10 x 10 x 0.1 mm, wired with a platinum wire), Potassium 4-methoxybenzene-trifluoroborate (43 mg, 0.2 mmol, 1 eq.) was dissolved in acetone (4 mL). Diphenylditelluride (409 mg, 1 mmol, 5 eq.) was added. The reaction mixture was sparged with nitrogen for

5 minutes, then a balloon of nitrogen was added to keep the vial under a positive pressure of nitrogen. The reaction was electrolyzed at 10 mA for 4 F/mol, an alternative polarity frequency of 0.5 Hz was applied. At the end of the reaction, the mixture was concentrated and directly purified via column chromatography (ethyl acetate/hexane gradient, 0–10%). Compound **22** was obtained as a colorless solid (55 mg, 88% yield). The NMR data matches literature.[13]

^{1}H NMR (499.8 MHz, $CDCl_3$) δ 7.72 (d, *J* = 8.8 Hz, 2H), 7.56 (dd, *J* = 8.1, 1.3 Hz, 2H), 7.26 – 7.12 (m, 3H), 6.79 (d, *J* = 8.9 Hz, 2H), 3.78 (s, 3H).

^{125}Te NMR (189.5 MHz, $CDCl_3$) δ 664.60 (s).

2-(Phenylseleno)benzofuran (**23**)

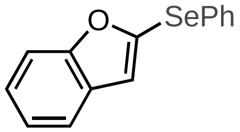

In an IKA Electrasyn vial equipped with two platinum plates (10 x 10 x 0.1 mm, wired with a platinum wire), Potassium benzofuran-2-trifluoroborate (45 mg, 0.2 mmol, 1 eq.) was dissolved in acetone (4 mL). Diphenyldiselenide (312 mg, 1 mmol, 5 eq.) was added. The reaction mixture was sparged with nitrogen for 5 minutes, then a balloon of nitrogen was added to keep the vial under a positive pressure of nitrogen. The reaction was electrolyzed at 10 mA for 4 F/mol, an alternative polarity frequency of 0.5 Hz was applied. At the end of the reaction, the mixture was concentrated and directly purified via column chromatography (ethyl acetate/hexane gradient, 0–10%). Compound **23** was obtained as a colorless oil (43 mg, 78% yield). The NMR data matches literature.[14]

^{1}H NMR (499.8 MHz, $CDCl_3$) δ 7.50 (d, *J* = 8.8 Hz, 2H), 7.35 – 7.30 (m, 2H), 7.26 – 7.14 (m, 3H), 6.85 (d, *J* = 8.9 Hz, 2H), 3.79 (s, 3H).

^{77}Se NMR (189.5 MHz, $CDCl_3$) δ 333.79 (s).

2-(Phenyltelluro)benzofuran (**24**)

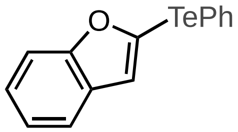

In an IKA Electrasyn vial equipped with two platinum plates (10 x 10 x 0.1 mm, wired with a platinum wire), Potassium benzofuran-2-trifluoroborate (45 mg, 0.2 mmol, 1 eq.) was dissolved in acetone (4 mL). Diphenylditelluride (409 mg, 1 mmol, 5 eq.) was added. The reaction mixture was sparged with nitrogen for 5 minutes, then a balloon of nitrogen was added to keep the vial under a positive pressure of nitrogen. The reaction was electrolyzed at 10 mA for 4 F/mol, an alternative polarity frequency of 0.5 Hz was applied. At the end of the reaction, the mixture was concentrated and directly purified via column chromatography (ethyl acetate/hexane gradient, 0–10%). Compound **24** was obtained as a colorless solid (53 mg, 82% yield). The NMR data matches literature.[15]

^{1}H NMR (499.8 MHz, $CDCl_3$) δ 7.72 (d, *J* = 8.8 Hz, 2H), 7.56 (dd, *J* = 8.1, 1.3 Hz, 2H), 7.26 – 7.12 (m, 3H), 6.79 (d, *J* = 8.9 Hz, 2H), 3.78 (s, 3H).

^{125}Te NMR (189.5 MHz, $CDCl_3$) δ 557.56 (s).

2,6-Dimethoxy-3-(phenylseleno)pyridine (**25**)

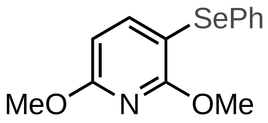

In an IKA Electrasyn vial equipped with two platinum plates (10 x 10 x 0.1 mm, wired with a platinum wire), Potassium 1,4-benzodioxane-6-trifluoroborate (49 mg, 0.2 mmol, 1 eq.) was dissolved in acetone (4 mL). Diphenyldiselenide (312 mg, 1 mmol, 5 eq.) was added. The reaction mixture was sparged with nitrogen for 5 minutes, then a balloon of nitrogen was added to keep the vial under a positive pressure of nitrogen. The reaction was electrolyzed at 10 mA for 4 F/mol, an alternative polarity frequency of 0.5 Hz was applied. At the end of the reaction, the mixture was concentrated and directly purified via column chromatography (ethyl acetate/hexane gradient, 0–20%). Compound **25** was obtained as a colorless oil (38 mg, 64% yield).

^{1}H NMR (499.8 MHz, $CDCl_3$) δ 7.49 (d, *J* = 8.1 Hz, 1H), 7.44 – 7.37 (m, 2H), 7.24 (dd, *J* = 5.0, 2.2 Hz, 3H), 6.25 (d, *J* = 8.1 Hz, 1H), 3.97 (s, 3H), 3.92 (s, 3H).

^{13}C NMR (125.7 MHz, $CDCl_3$) δ 163.46, 161.33, 146.36, 132.39, 130.78, 129.37, 127.19, 102.44, 54.18, 53.81.

^{77}Se NMR (189.5 MHz, $CDCl_3$) δ 330.03 (s).

HRMS (ES-MS^{+}): exact mass (monoisotopic) calcd. For [M+] ($C_{13}H_{13}NO_2Se$): 295.0112; found, 295.0105

2,6-Dimethoxy-3-(phenyltelluro)pyridine (**26**)

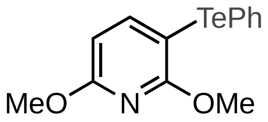

In an IKA Electrasyn vial equipped with two platinum plates (10 x 10 x 0.1 mm, wired with a platinum wire), Potassium 1,4-benzodioxane-6-trifluoroborate (49 mg, 0.2 mmol, 1 eq.) was dissolved in acetone (4 mL). Diphenylditelluride (409 mg, 1 mmol, 5 eq.) was added. The reaction mixture was sparged with nitrogen for 5 minutes, then a balloon of nitrogen was added to keep the vial under a positive pressure of nitrogen. The reaction was electrolyzed at 10 mA for 4 F/mol, an alternative polarity frequency of 0.5 Hz was applied. At the end of the reaction, the mixture was concentrated and directly purified via column chromatography (ethyl acetate/hexane gradient, 0–20%). Compound **26** was obtained as a slightly yellow crystalline solid (48 mg, 70% yield).

^{1}H NMR (499.8 MHz, $CDCl_3$) δ 7.80 – 7.71 (m, 2H), 7.40 (d, *J* = 8.1 Hz, 1H), 7.32 (tt, J = 7.5, 2 Hz, 1H), 7.24 (t, *J* = 7.3 Hz, 2H), 6.19 (d, *J* = 8.1 Hz, 1H), 3.96 (s, 3H), 3.90 (s, 3H).

^{13}C NMR (125.7 MHz, $CDCl_3$) δ 163.97, 161.97, 147.95, 139.35, 129.66, 128.31, 113.16, 103.16, 86.60, 54.20, 53.68.

^{125}Te NMR (189.5 MHz, $CDCl_3$) δ 561.67 (s).

HRMS (ES-MS$^+$): exact mass (monoisotopic) calcd. For [M+] ($C_{13}H_{13}NO_2Te$): 345.0009; found, 345.0008.

7. Copies of ^{1}H, ^{13}C, ^{31}P and other heteronulear NMR spectra

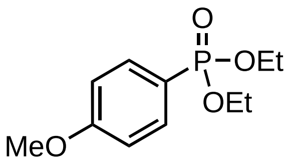

2

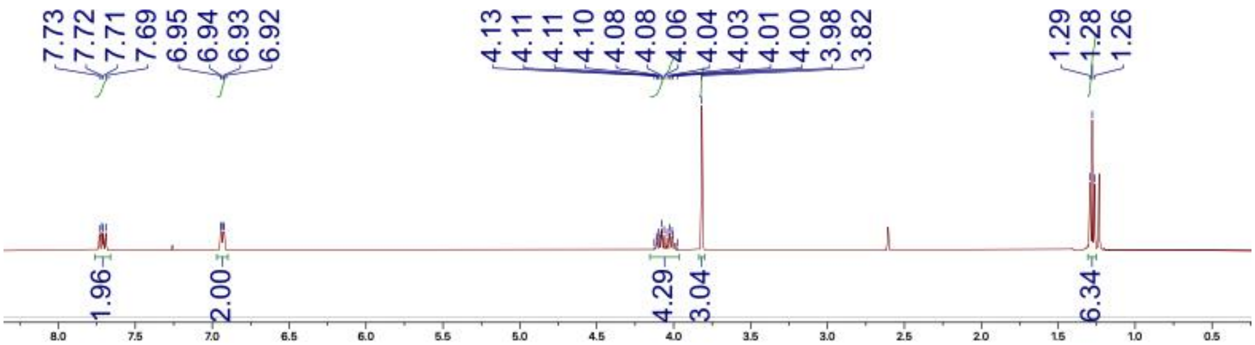

Figure S6 : ^{1}H NMR (499.8 MHz, $CDCl_3$) of compound **2**.

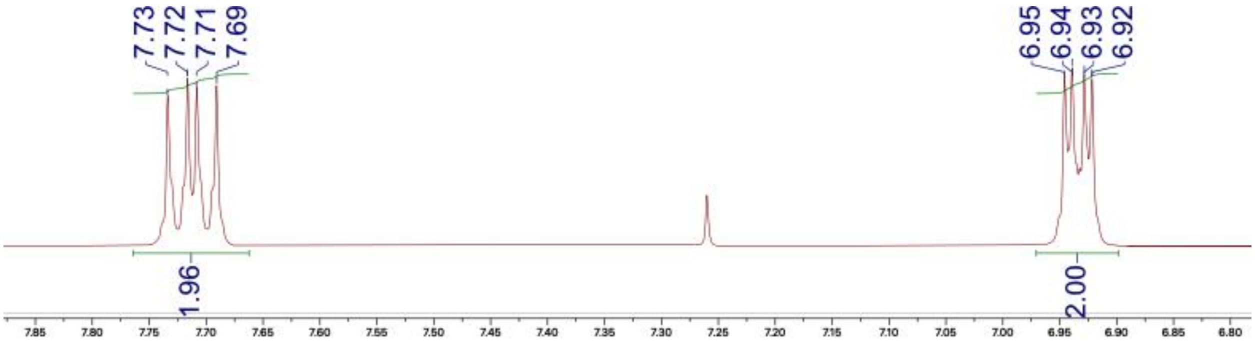

Figure S7 : ^{1}H NMR ($CDCl_3$, 499.8 MHz) of compound **2**, aromatic region.

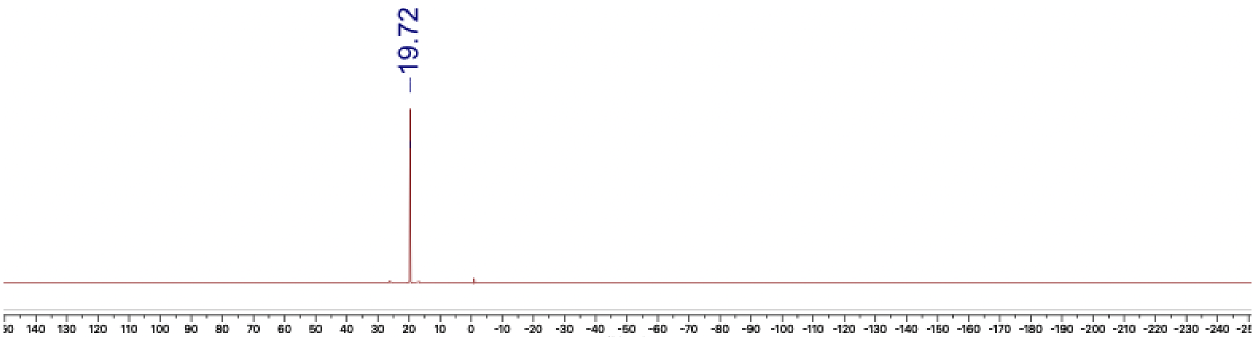

Figure S8 : ^{31}P NMR ($CDCl_3$, 202.3 MHz) of compound **2**.

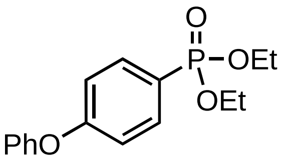

3

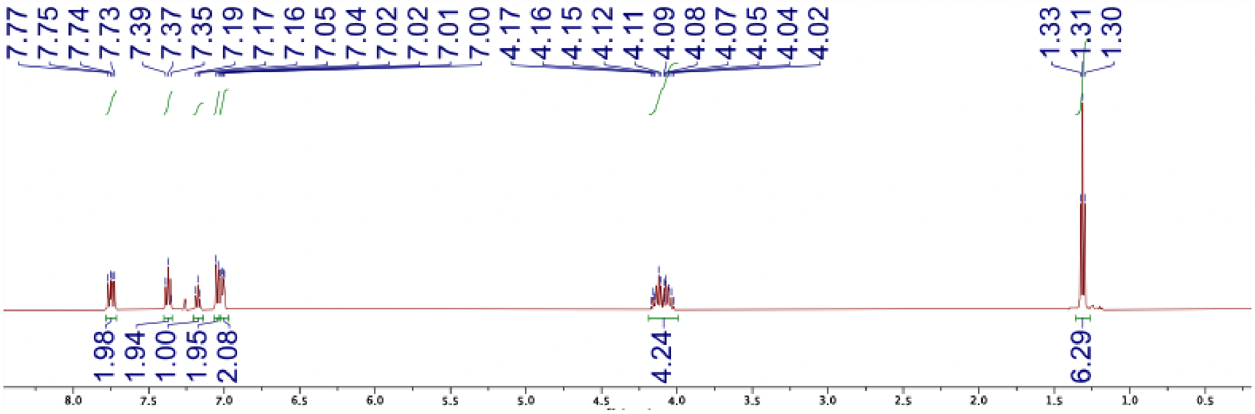

Figure S9 : ^{1}H NMR (499.8 MHz, $CDCl_3$) of compound **3**.

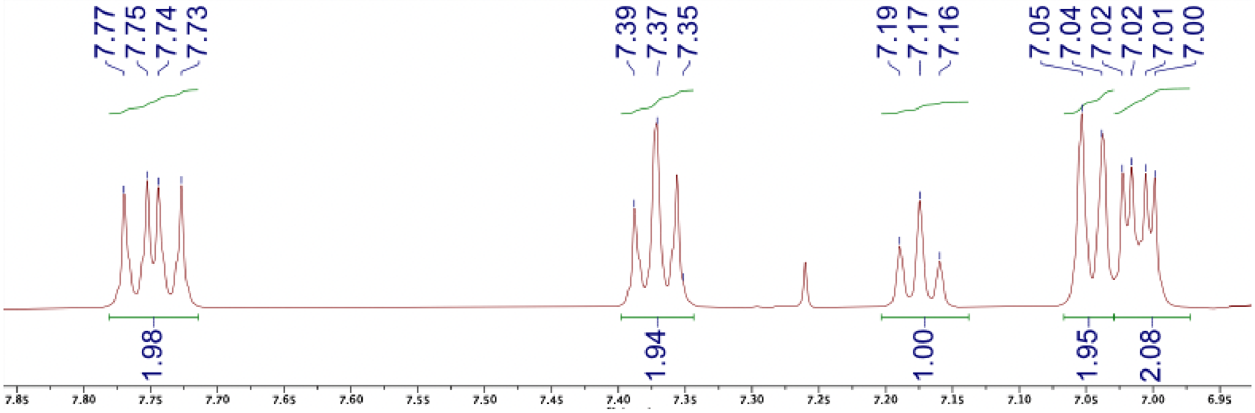

Figure S10 : ^{1}H NMR ($CDCl_3$, 499.8 MHz) of compound **3**, aromatic region.

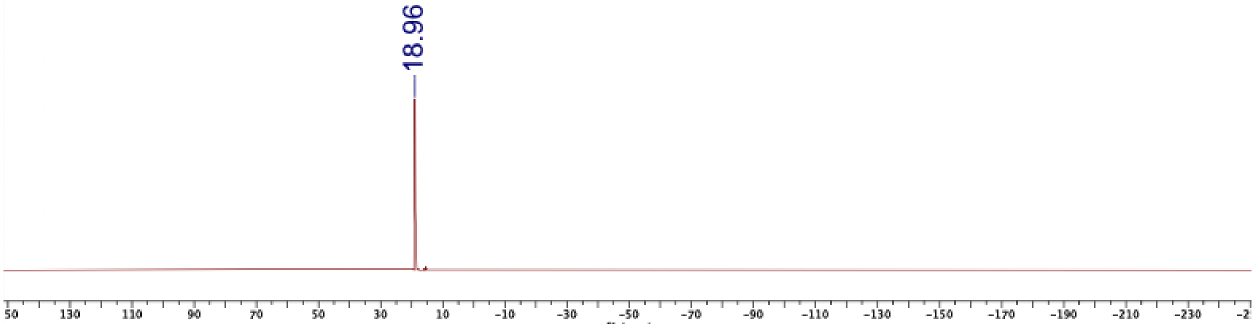

Figure S11 : ^{31}P NMR ($CDCl_3$, 202.3 MHz) of compound **3**.

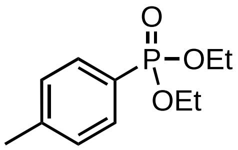

4

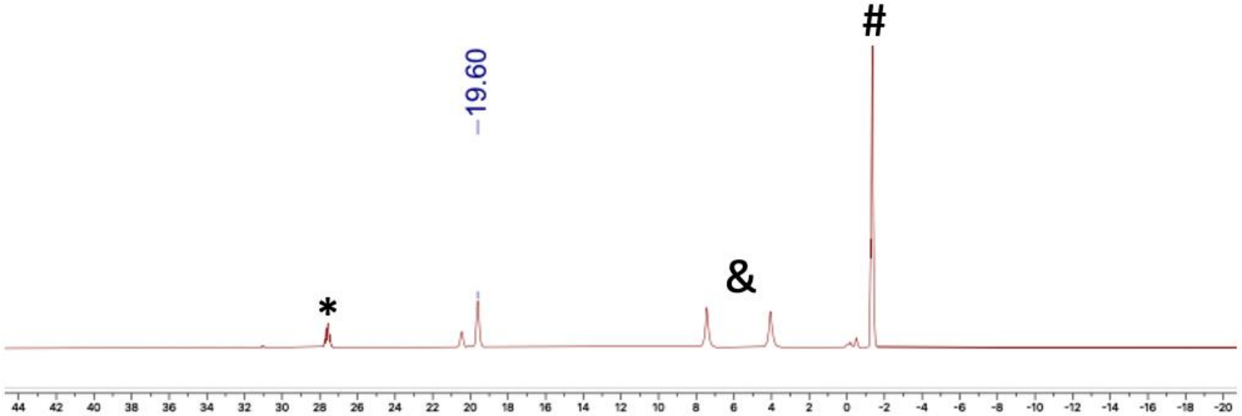

Figure S12 : ^{31}P NMR ($CDCl_3$, 202.3 MHz) of compound **4**. * denotes for PPh_3PO. & denotes for $HP(OEt)_2$. # denotes for $OP(OEt)_3$.

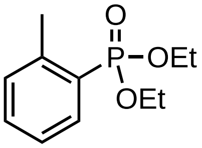

5

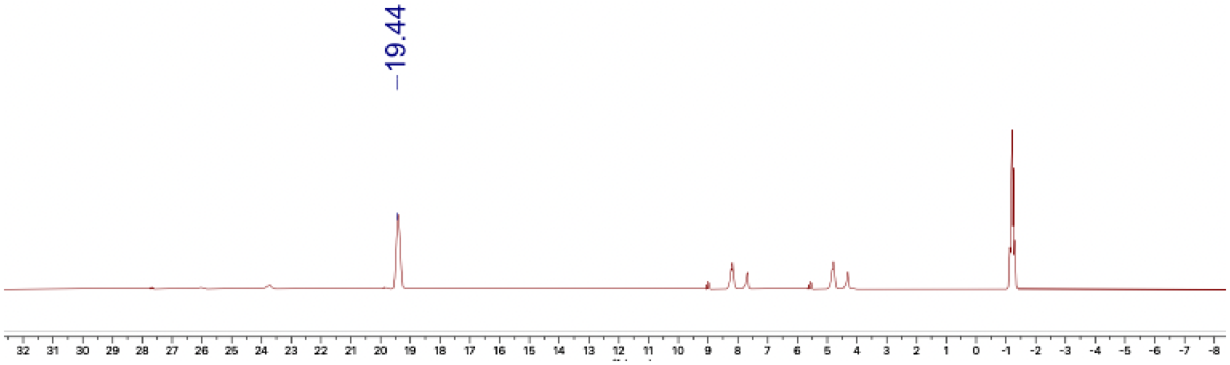

Figure S13 : ^{31}P NMR ($CDCl_3$, 202.3 MHz) of compound **5**.

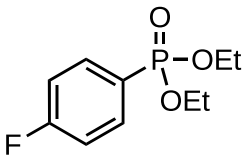

6

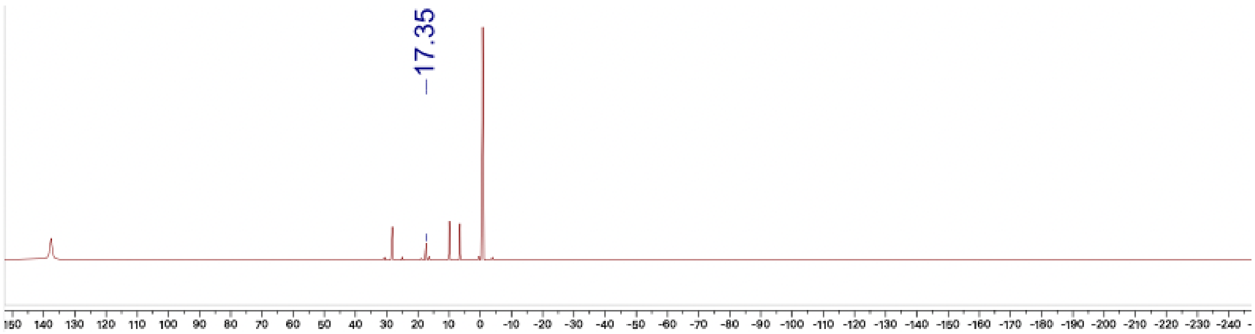

Figure S14 : ^{31}P NMR ($CDCl_3$, 202.3 MHz) of compound **6.**

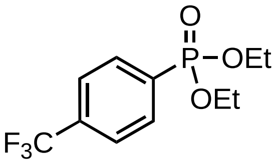

7

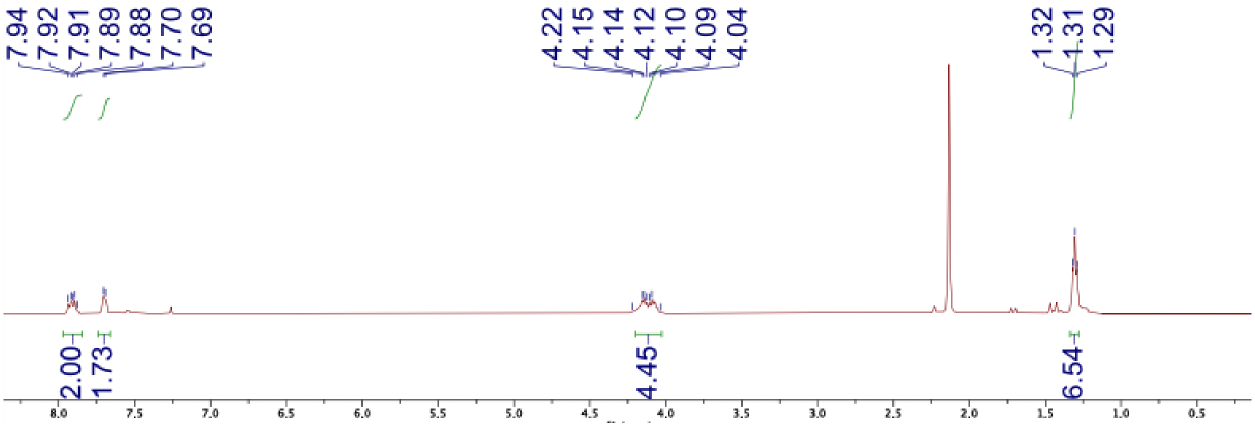

Figure S15 : ^{1}H NMR (499.8 MHz, $CDCl_3$) of compound **7**.

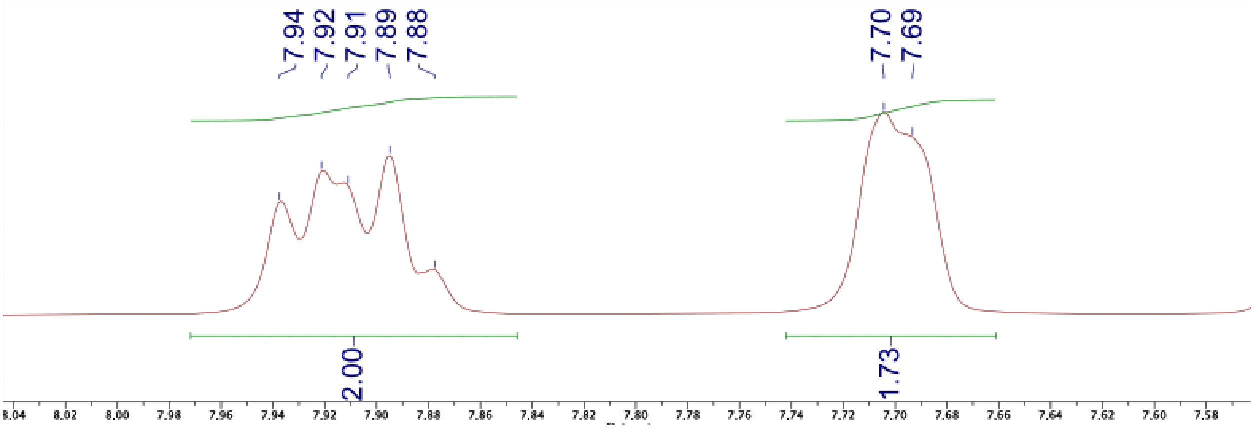

Figure S16 : ^{1}H NMR (499.8 MHz, $CDCl_3$) of compound **7**, aromatic region.

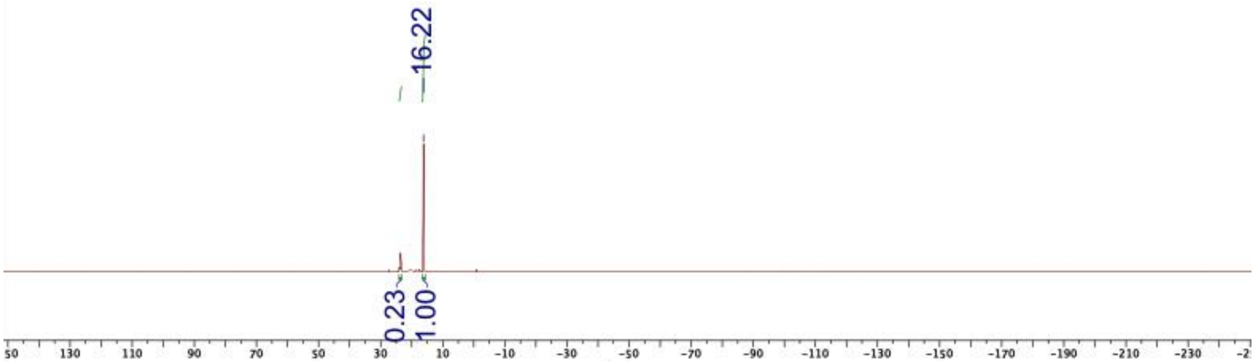

Figure S17 : ^{31}P NMR ($CDCl_3$, 202.3 MHz) of compound **7**.

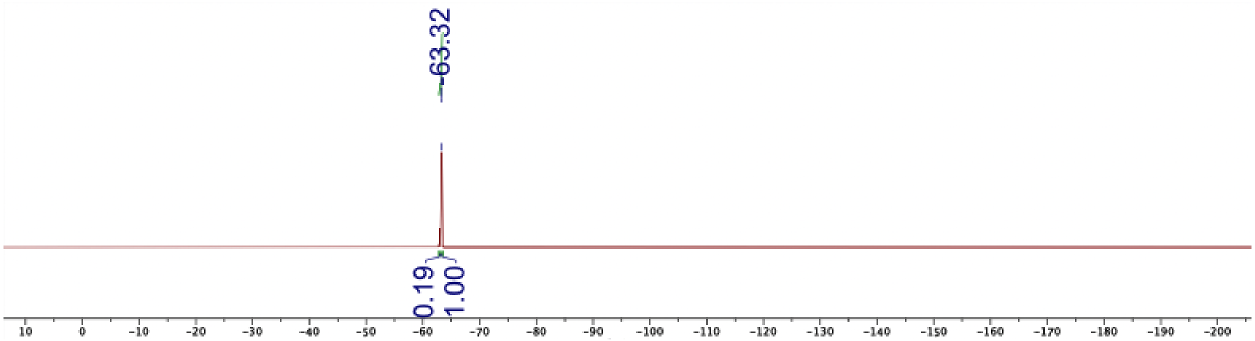

Figure S18 : ^{19}F NMR (470.3 MHz, $CDCl_3$) of compound **7**.

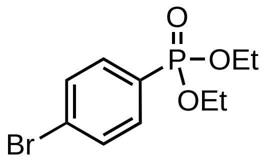

8

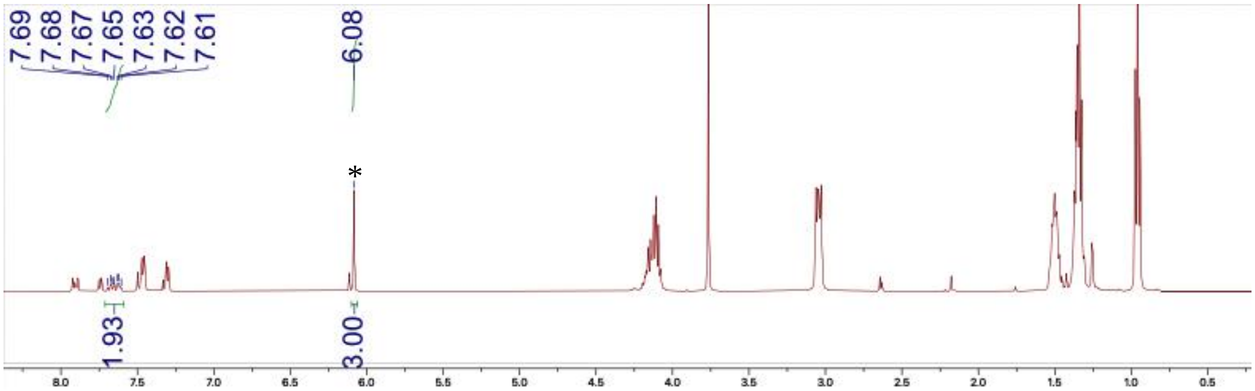

Figure S19 : ^{1}H NMR (499.8 MHz, $CDCl_3$) of compound **8** in crude mixture. * denotes for trimethoxybenene internal standard.

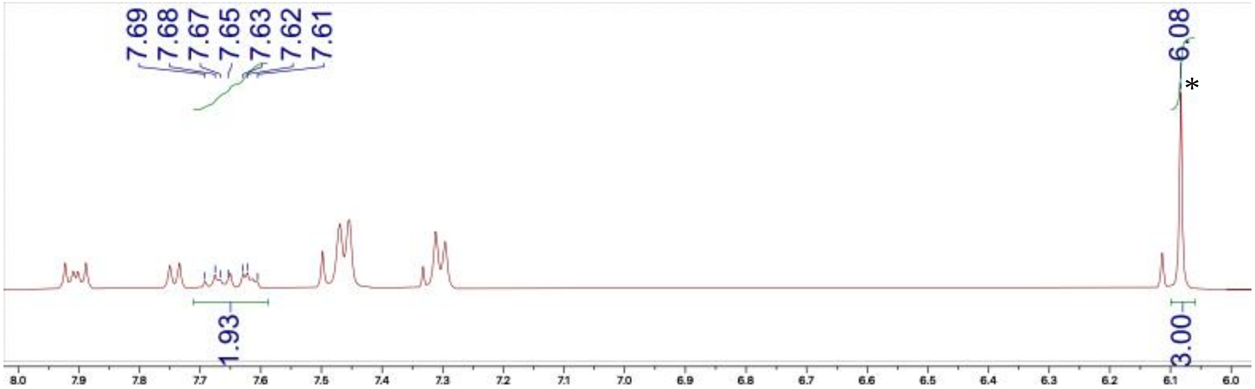

Figure S20: ^{1}H NMR (499.8 MHz, $CDCl_3$) of compound **8** in crude mixture, aromatic region. * denotes for trimethoxybenene internal standard.

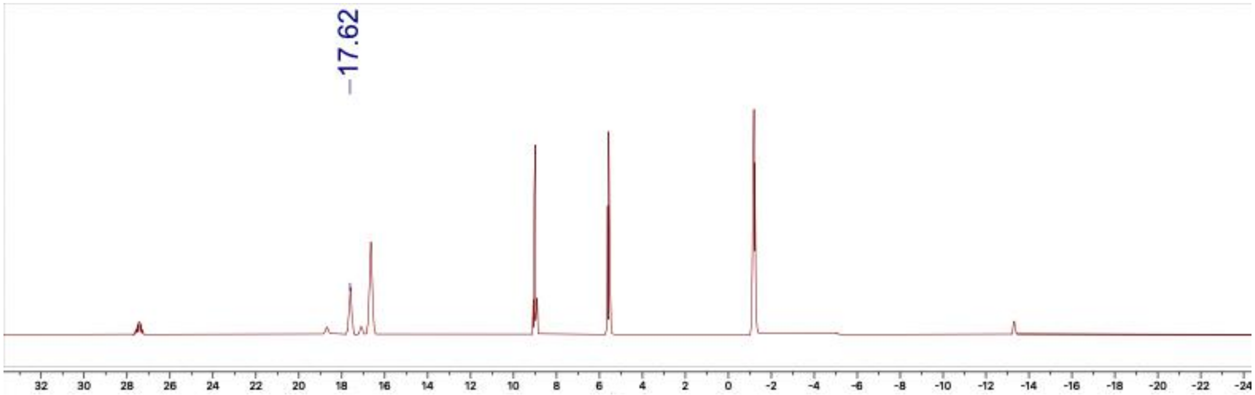

Figure S21 : ^{31}P NMR ($CDCl_3$, 202.3 MHz) of compound **8** in crude mixture.

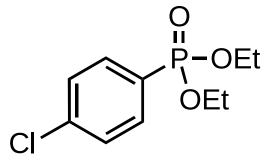

9

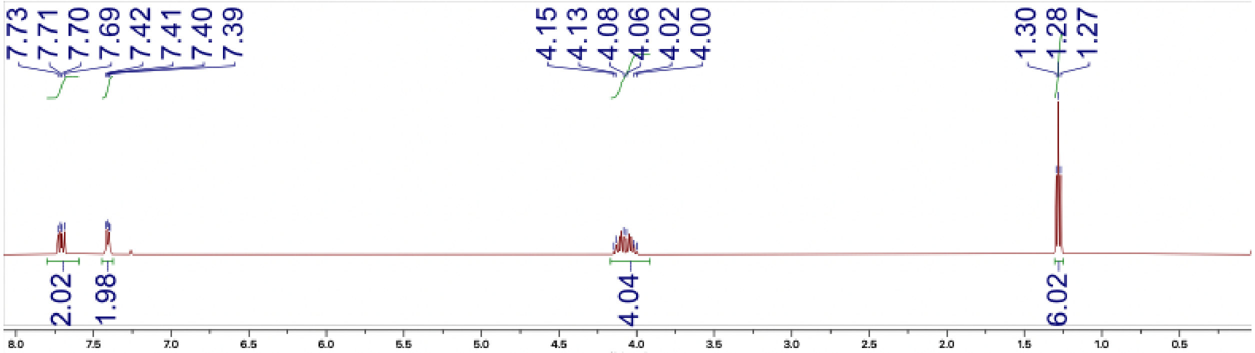

Figure S22 : ^{1}H NMR (499.8 MHz, $CDCl_3$) of compound **9**.

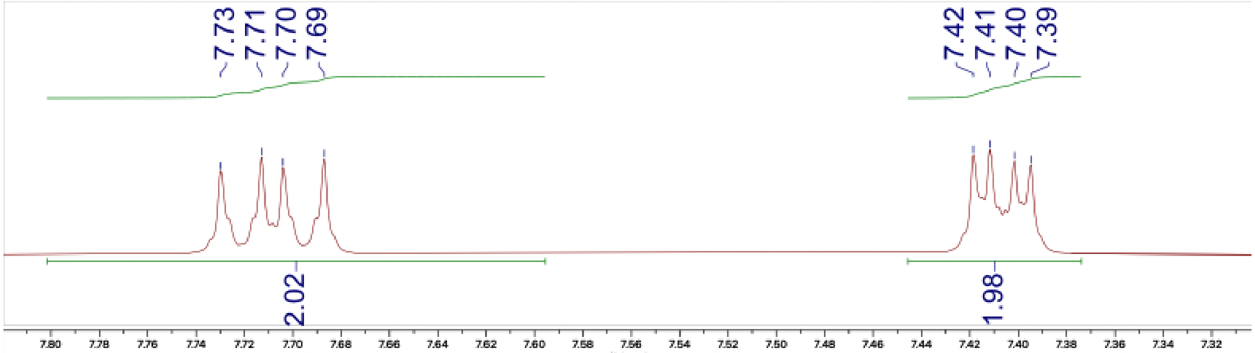

Figure S23 : ^{1}H NMR ($CDCl_3$, 499.8 MHz) of compound **9**, aromatic region.

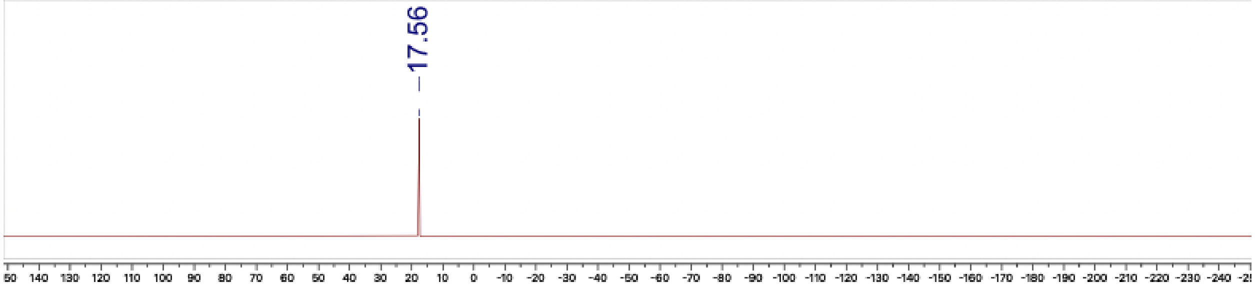

Figure S24 : ^{31}P NMR ($CDCl_3$, 202.3 MHz) of compound **9**.

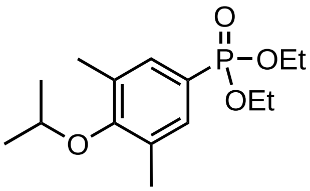

10

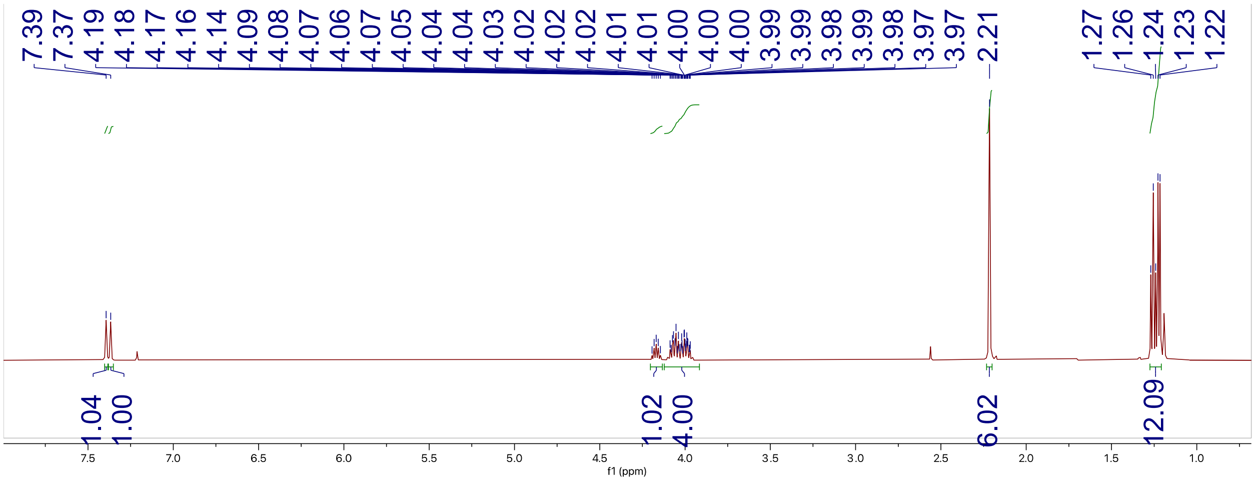

Figure S25 : ^{1}H NMR (499.8 MHz, $CDCl_3$) of compound **10**.

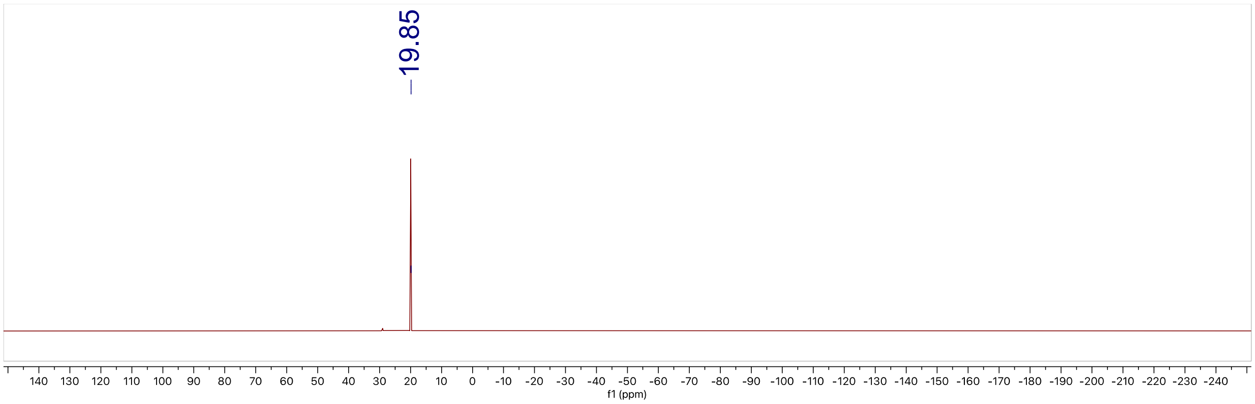

Figure S26 : ^{31}P NMR ($CDCl_3$, 202.3 MHz) of compound **10.**

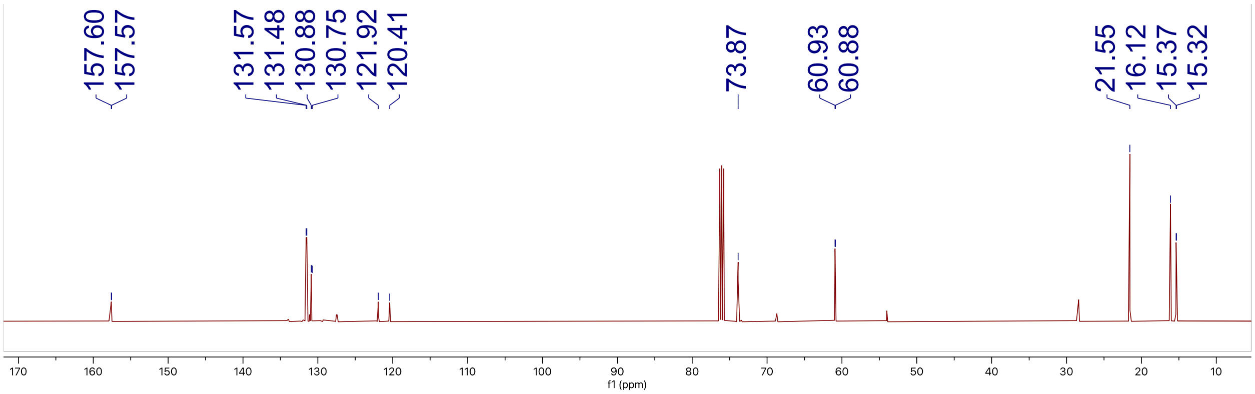

Figure S27 : ^{13}C NMR ($CDCl_3$, 125.7 MHz) of compound **10**.

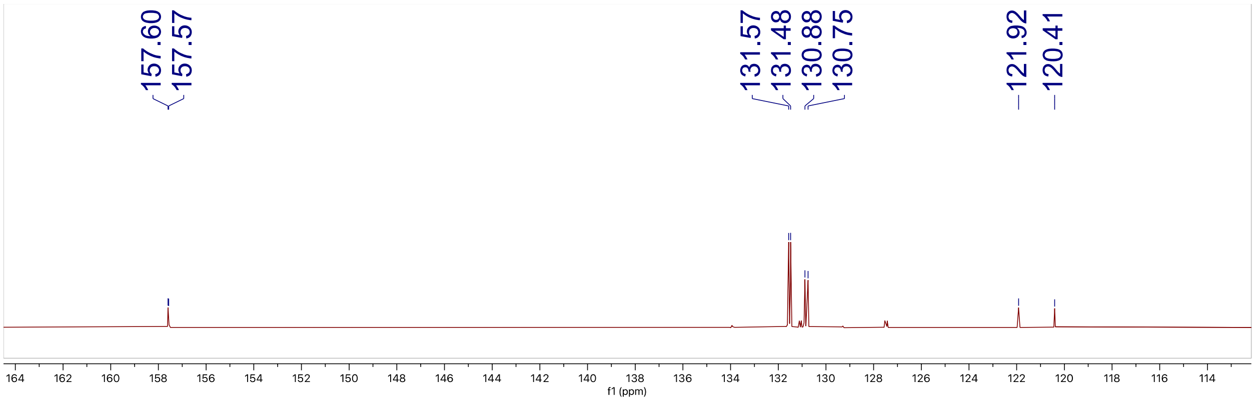

Figure S28: ^{13}C NMR ($CDCl_3$, 125.7 MHz) of compound **10,** aromatic region.

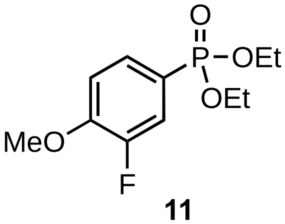

11

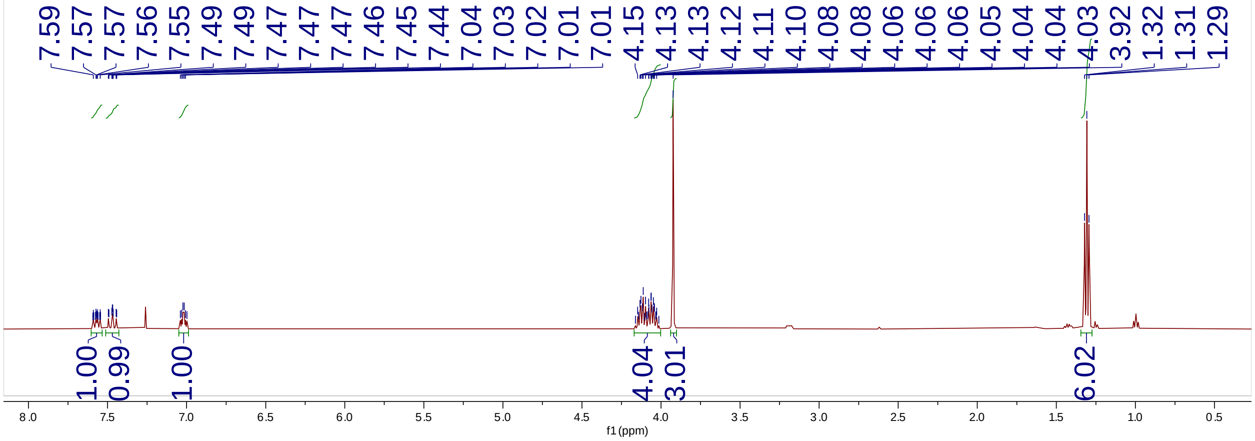

Figure S29: ^{1}H NMR (499.8 MHz, $CDCl_3$) of compound **11**.

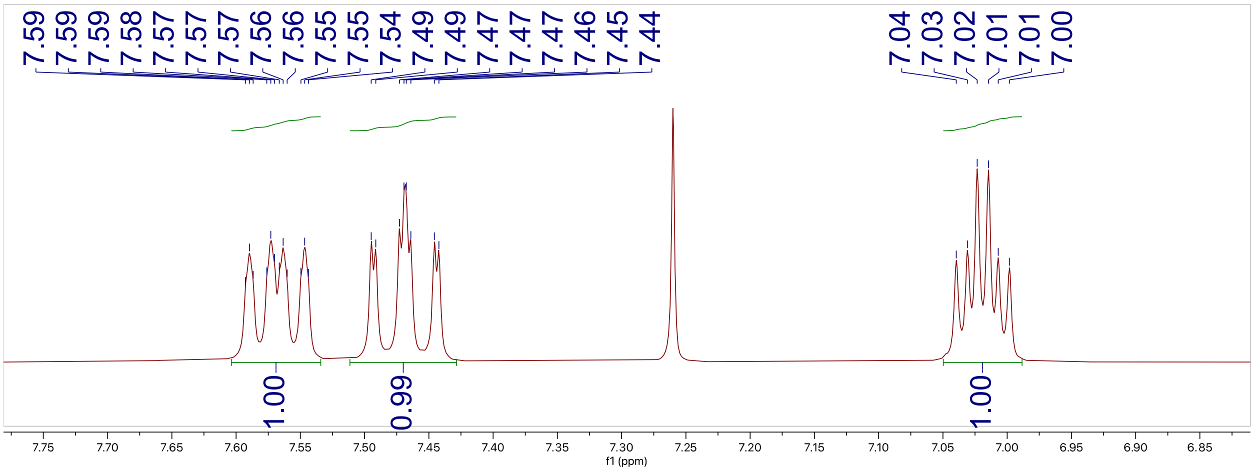

Figure S30: ^{1}H NMR (499.8 MHz, $CDCl_3$) of compound **11,** aromatic region.

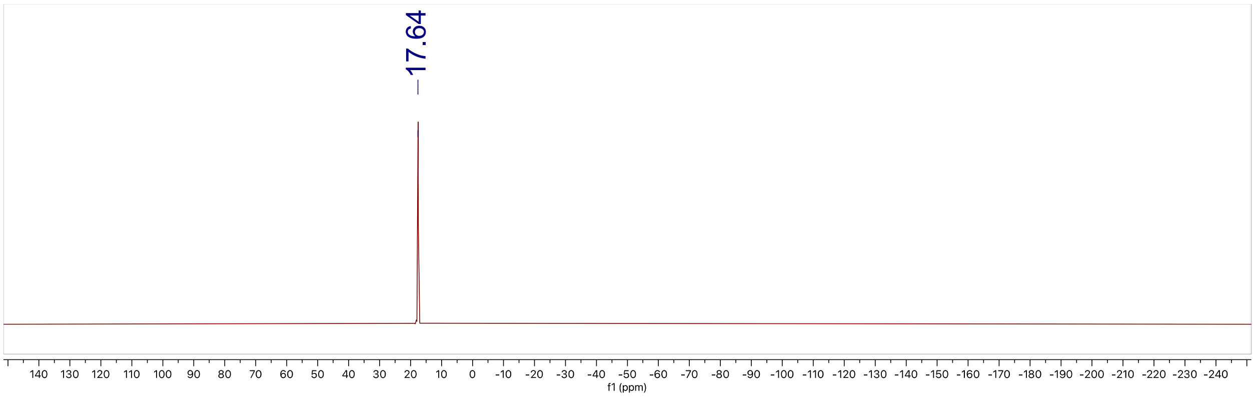

Figure S31 : ^{31}P NMR ($CDCl_3$, 202.3 MHz) of compound **11.**

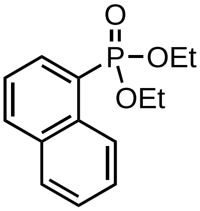

12

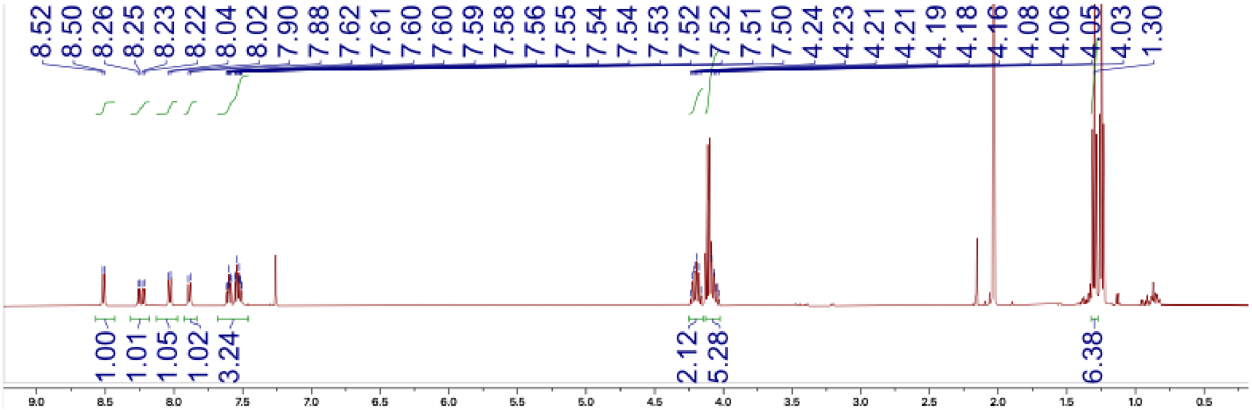

Figure S32: ^{1}H NMR (499.8 MHz, $CDCl_3$) of compound **12**.

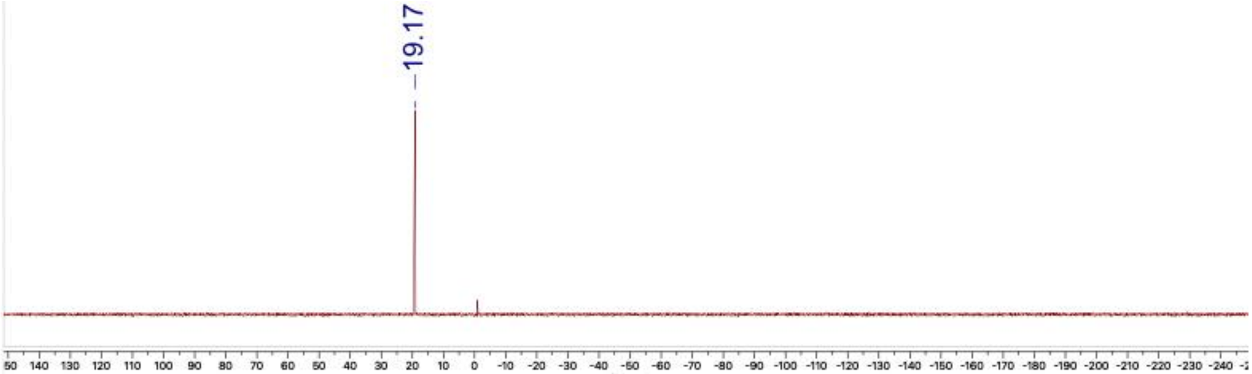

Figure S33: ^{31}P NMR ($CDCl_3$, 202.3 MHz) of compound **12.**

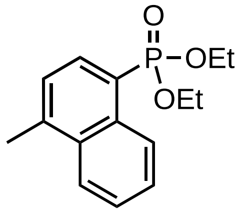

13

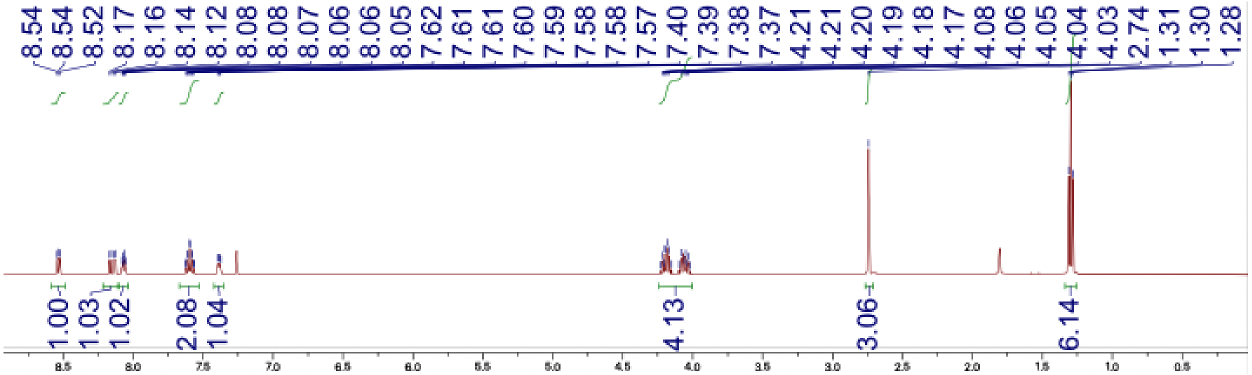

Figure S34: ^{1}H NMR ($CDCl_3$, 499.8 MHz) of compound **13**.

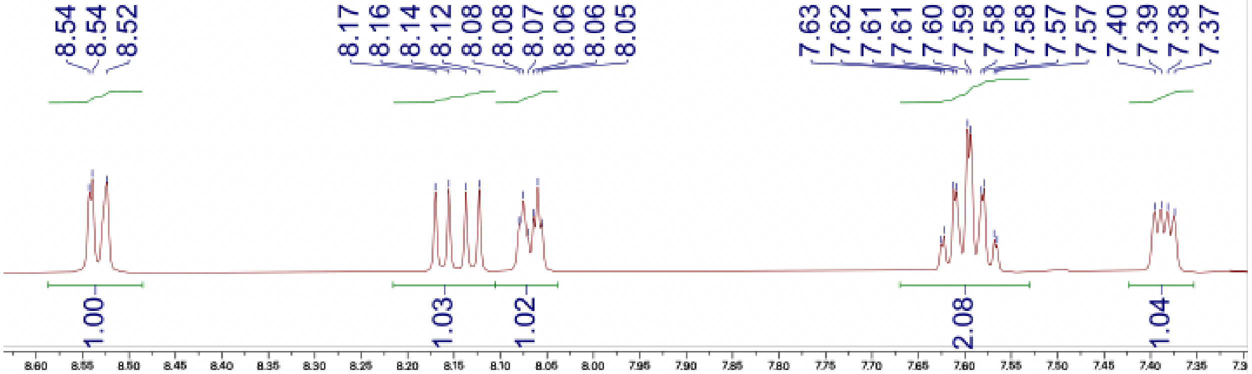

Figure S35: ^{1}H NMR ($CDCl_3$, 499.8 MHz) of compound **13**, aromatic region.

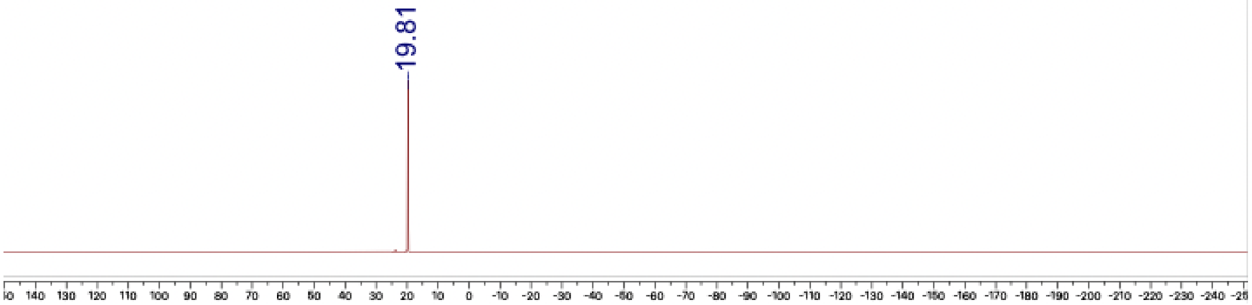

Figure S36: ^{31}P NMR ($CDCl_3$, 202.3 MHz) of compound **13** in crude mixture.

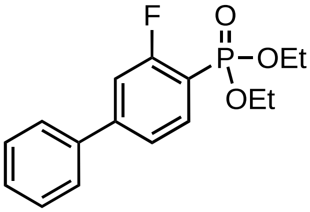

14

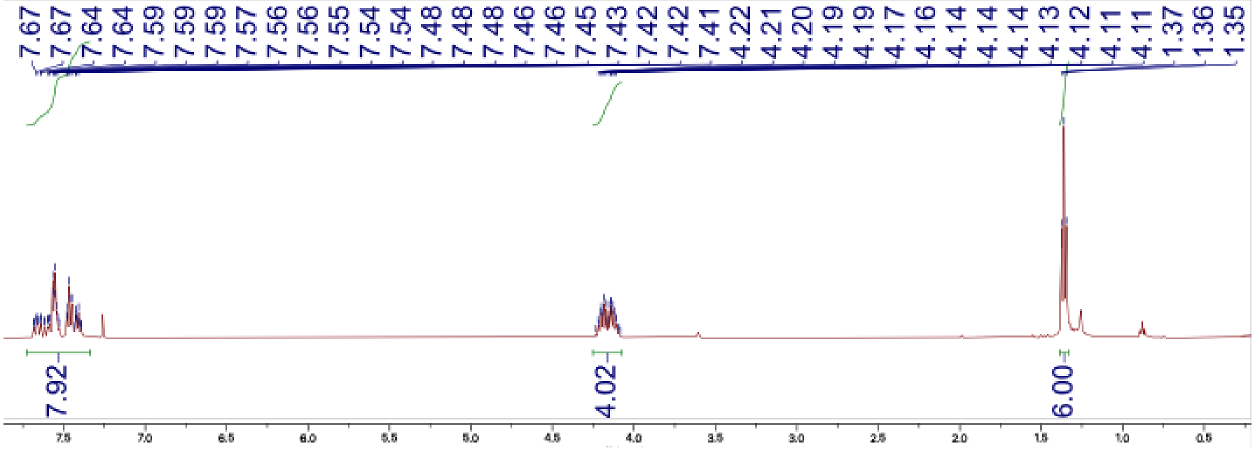

Figure S37: ^{1}H NMR ($CDCl_3$, 499.8 MHz) of compound **14**.

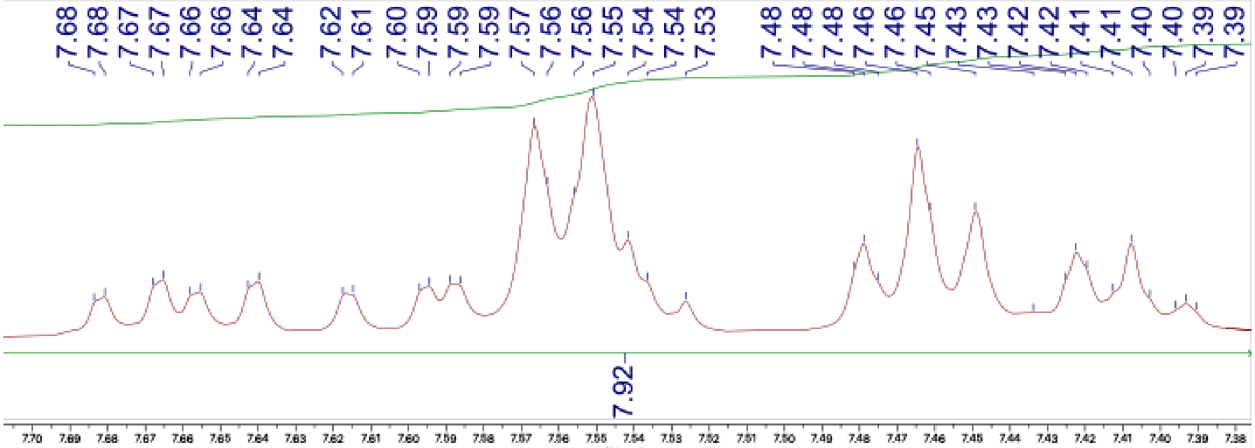

Figure S38: ^{1}H NMR ($CDCl_3$, 499.8 MHz) of compound **14**, aromatic region.

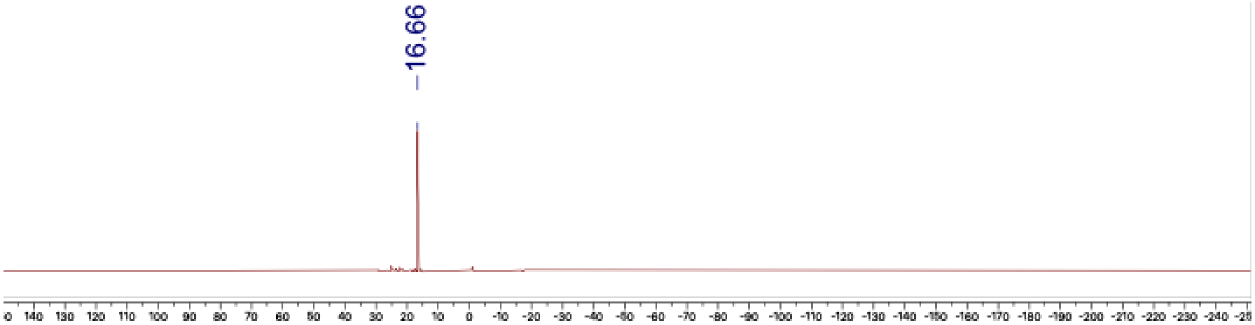

Figure S39: ^{31}P NMR ($CDCl_3$, 202.3 MHz) of compound **14**.

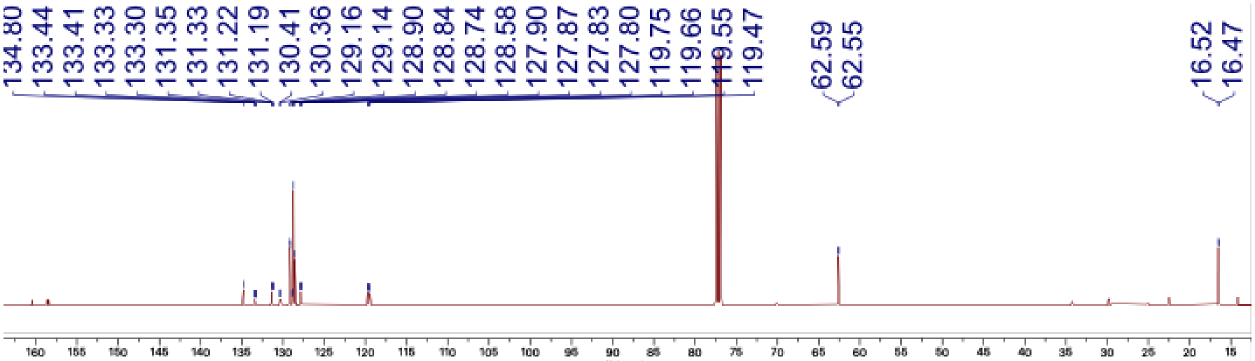

Figure S40: ^{13}C NMR ($CDCl_3$, 125.7 MHz) of compound **14**.

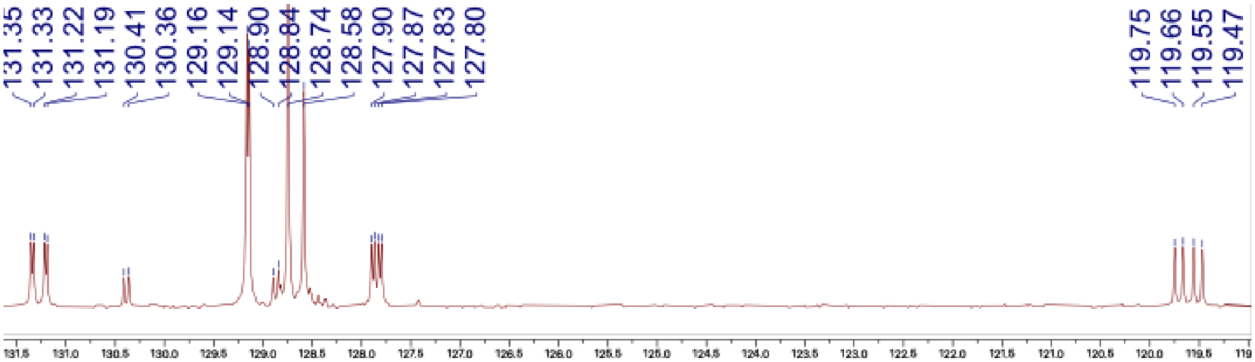

Figure S41: ^{13}C NMR ($CDCl_3$, 125.7 MHz) of compound **14**, aromatic region.

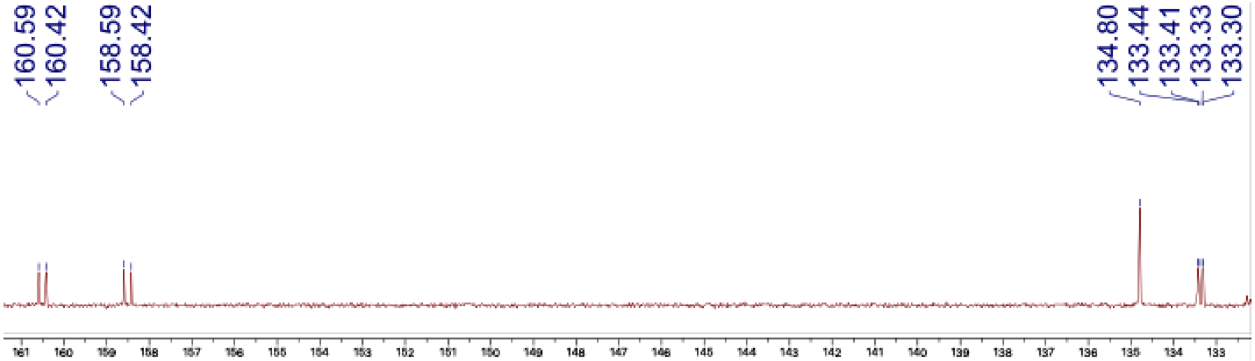

Figure S42: ^{13}C NMR ($CDCl_3$, 125.7 MHz) of compound **14**, aromatic region 2.

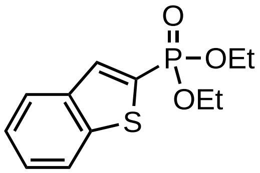

15

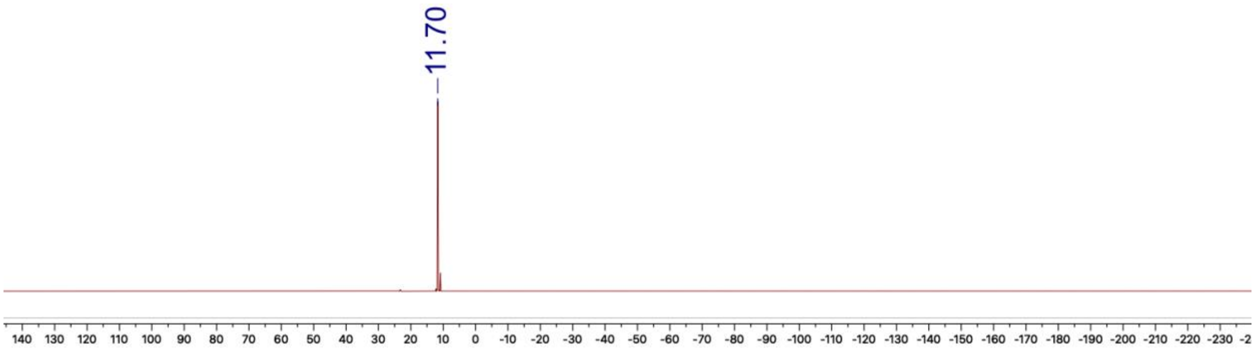

Figure S43: ^{31}P NMR ($CDCl_3$, 202.3 MHz) of compound **15**.

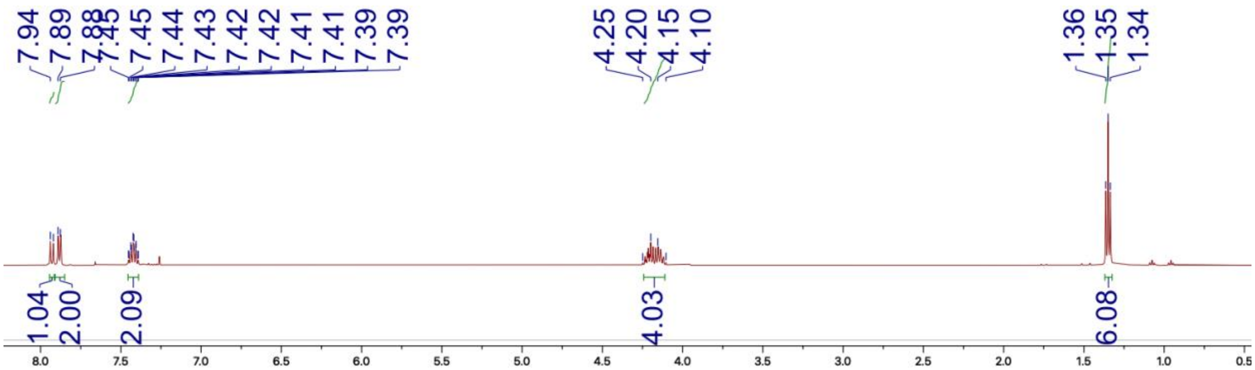

Figure S44: ^{1}H NMR ($CDCl_3$, 499.8 MHz) of compound **15**.

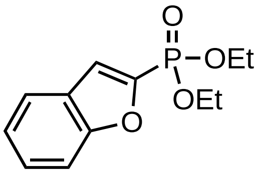

16

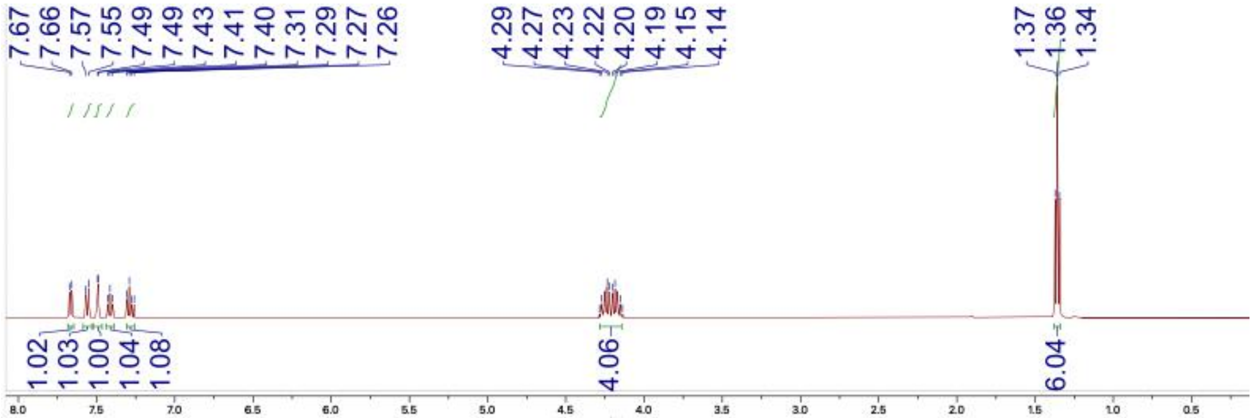

Figure S45: ^{1}H NMR (499.8 MHz, $CDCl_3$) of compound **16**.

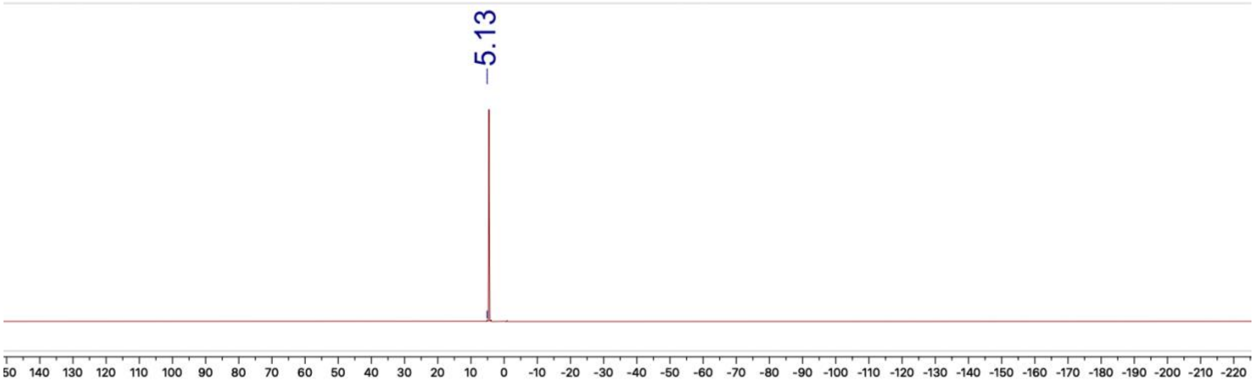

Figure S46: ^{31}P NMR ($CDCl_3$, 202.3 MHz) of compound **16**.

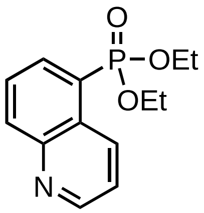

17

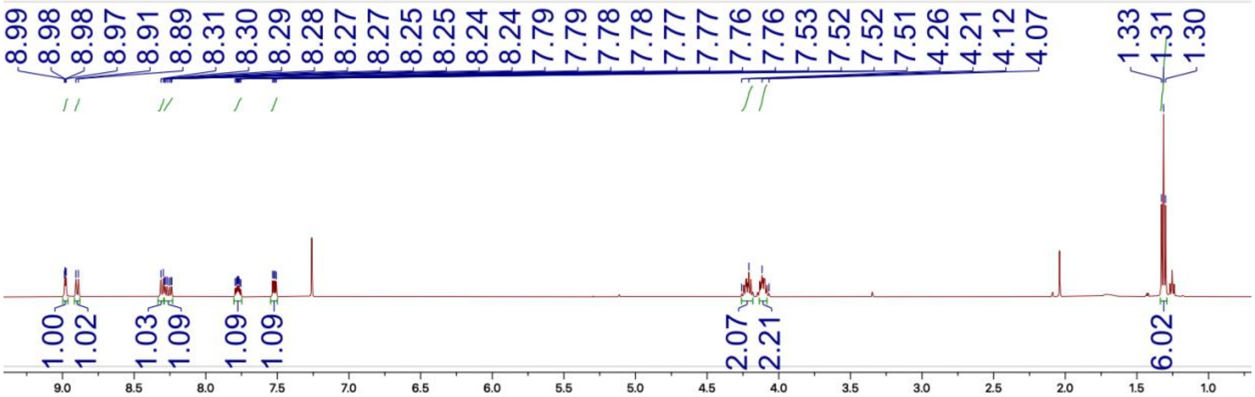

Figure S47: ^{1}H NMR ($CDCl_3$, 499.8 MHz) of compound **17**.

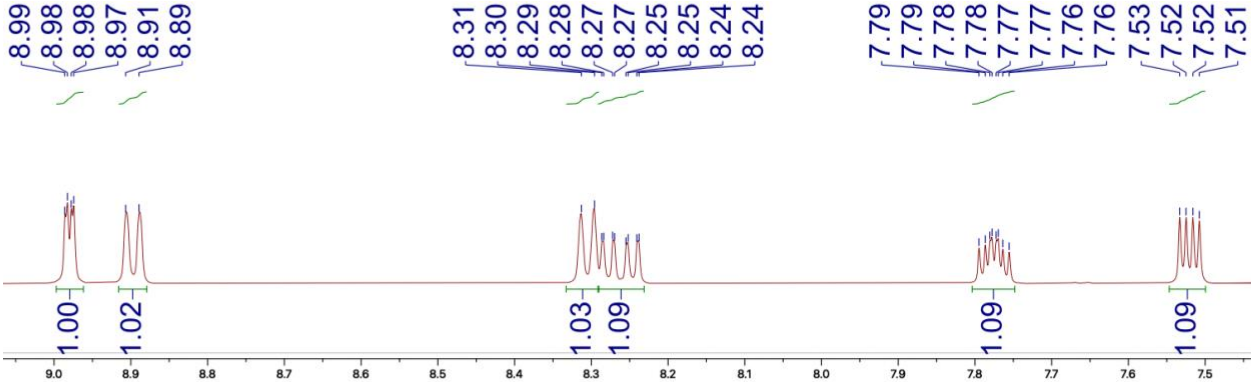

Figure S48: ^{1}H NMR ($CDCl_3$, 499.8 MHz) of compound **17**, aromatic region.

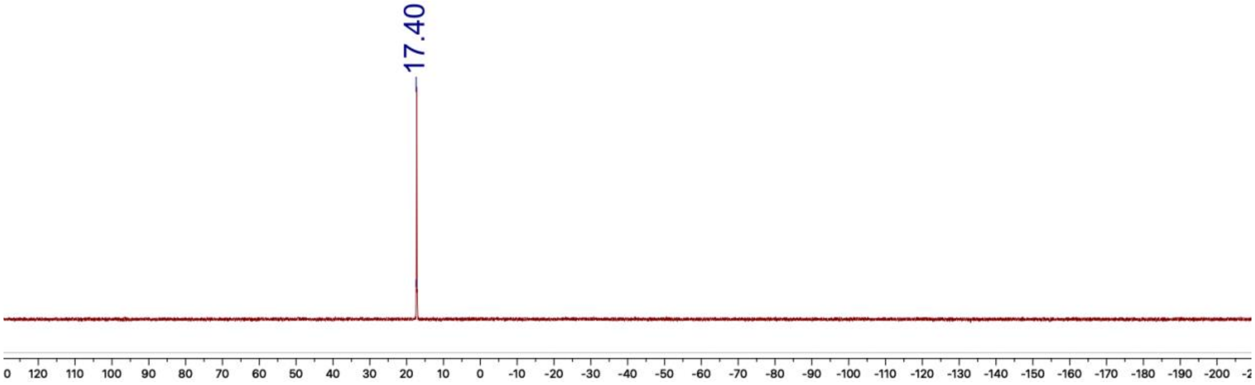

Figure S49: ^{31}P NMR ($CDCl_3$, 202.3 MHz) of compound **17**.

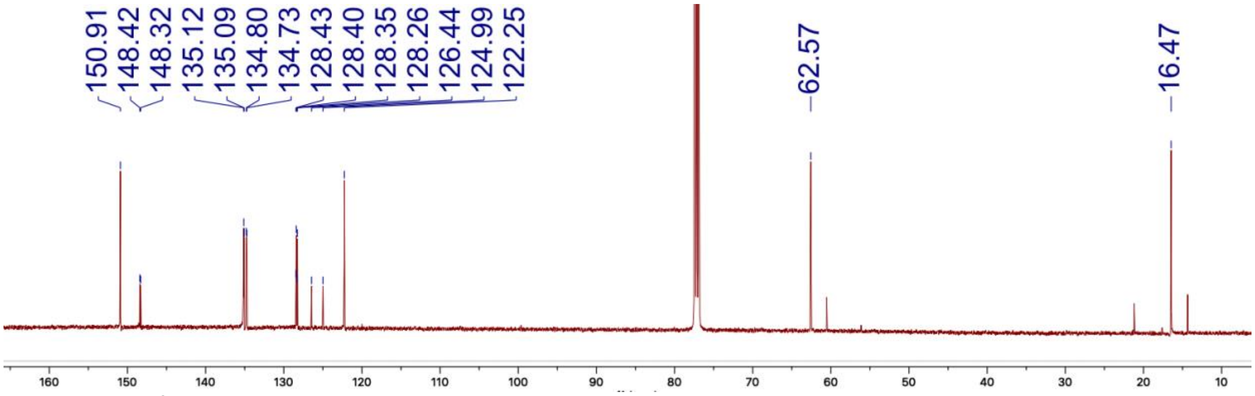

Figure S50: ^{13}C NMR ($CDCl_3$, 125.7 MHz) of compound **17**.

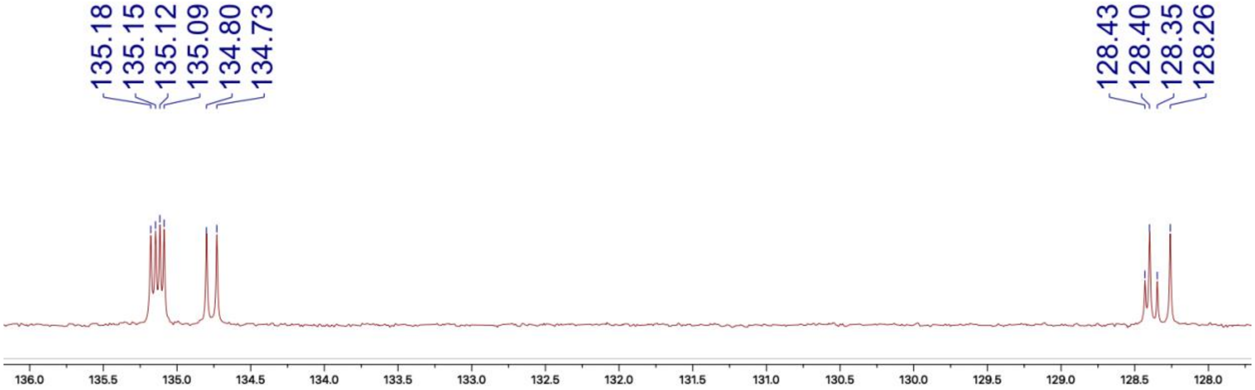

Figure S51: ^{13}C NMR ($CDCl_3$, 125.7 MHz) of compound **17**, aromatic region.

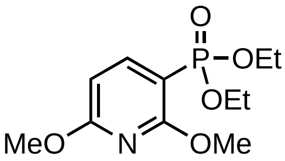

18

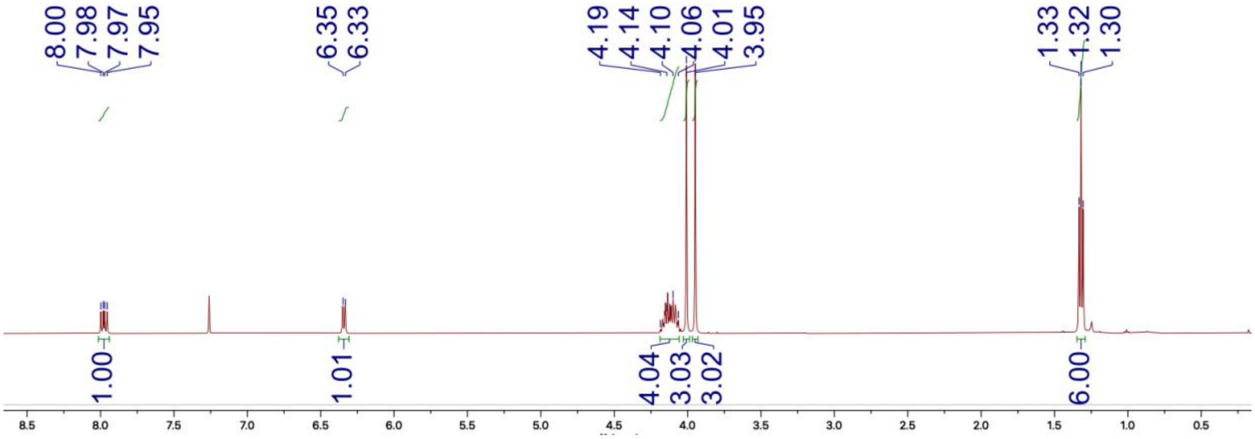

Figure S52: ^{1}H NMR ($CDCl_3$, 499.8 MHz) of compound **18**.

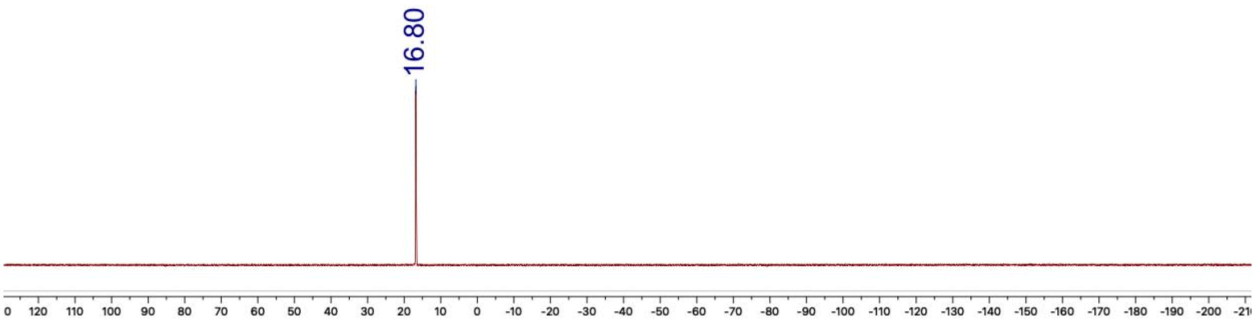

Figure S53: ^{31}P NMR ($CDCl_3$, 202.3 MHz) of compound **18**.

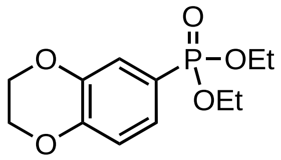

19

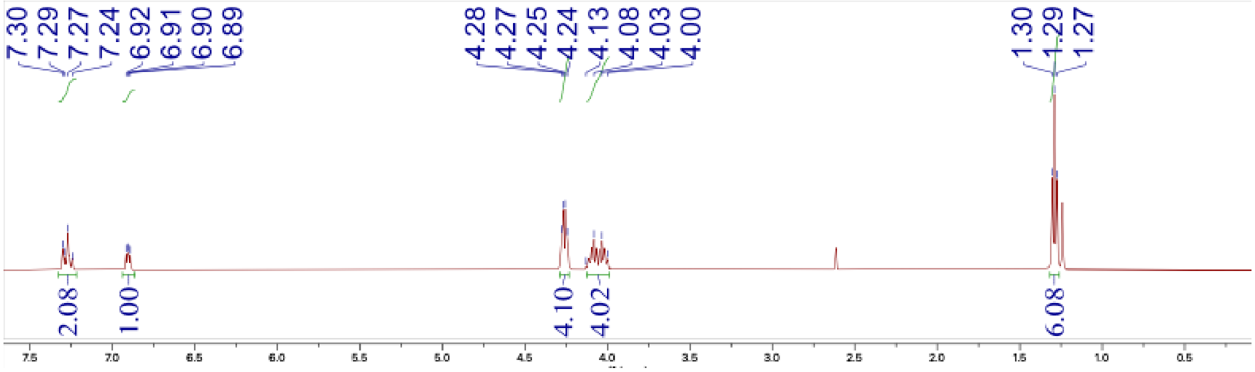

Figure S54: ^{1}H NMR ($CDCl_3$, 499.8 MHz) of compound **19**.

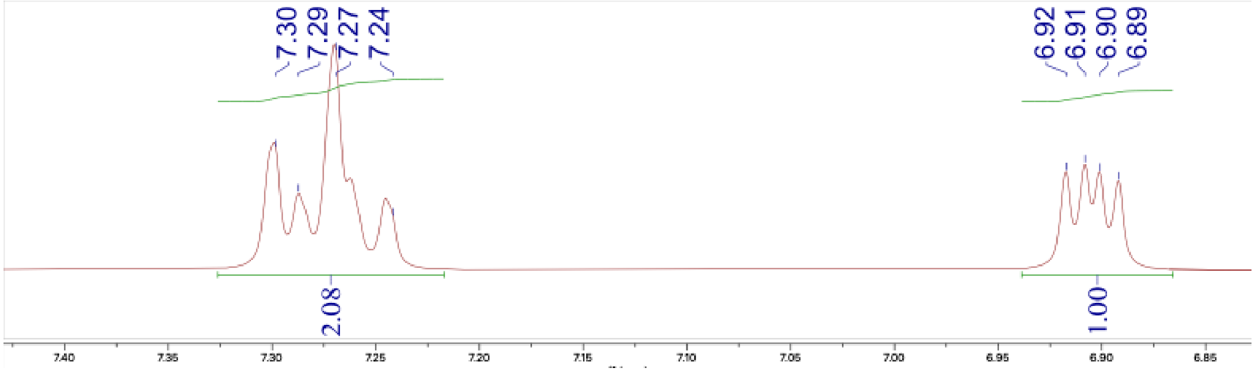

Figure S55: ^{1}H NMR ($CDCl_3$, 499.8 MHz) of compound **19**, aromatic region.

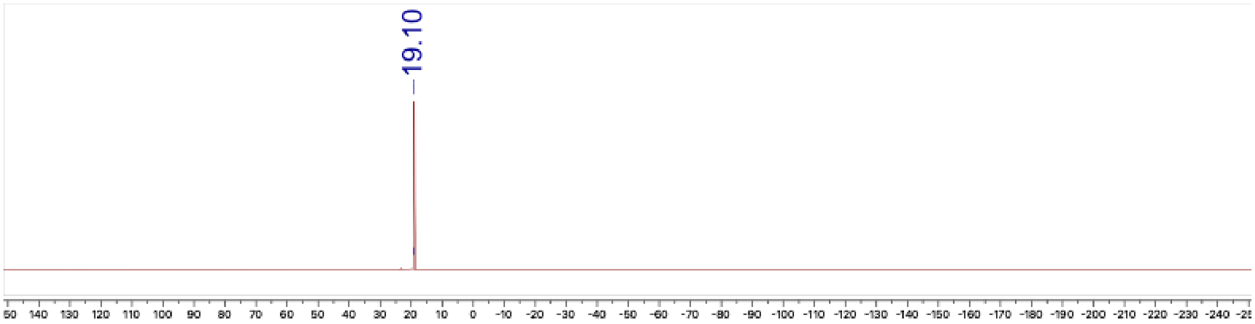

Figure S56: ^{31}P NMR ($CDCl_3$, 202.3 MHz) of compound **19**.

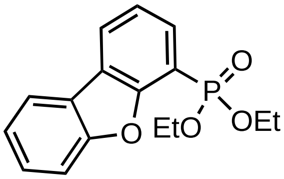

20

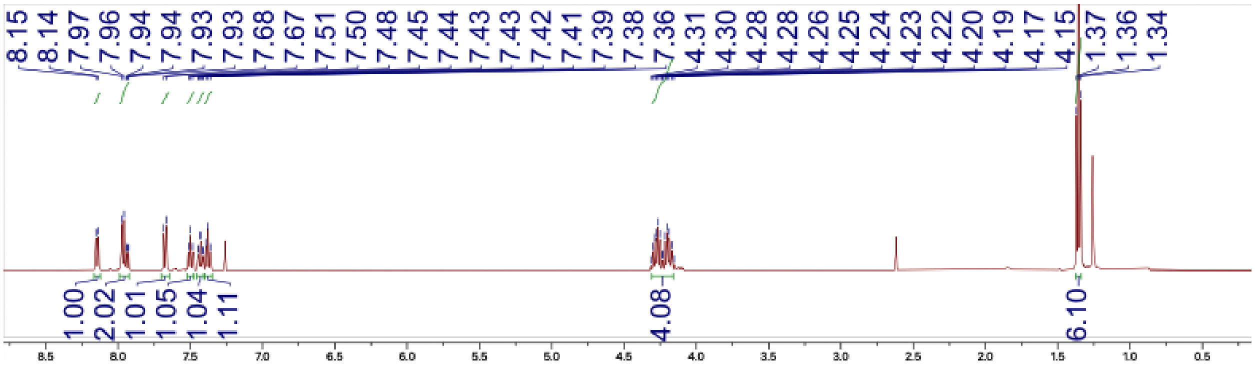

Figure S57: ^{1}H NMR (499.8 MHz, $CDCl_3$) of compound **20**.

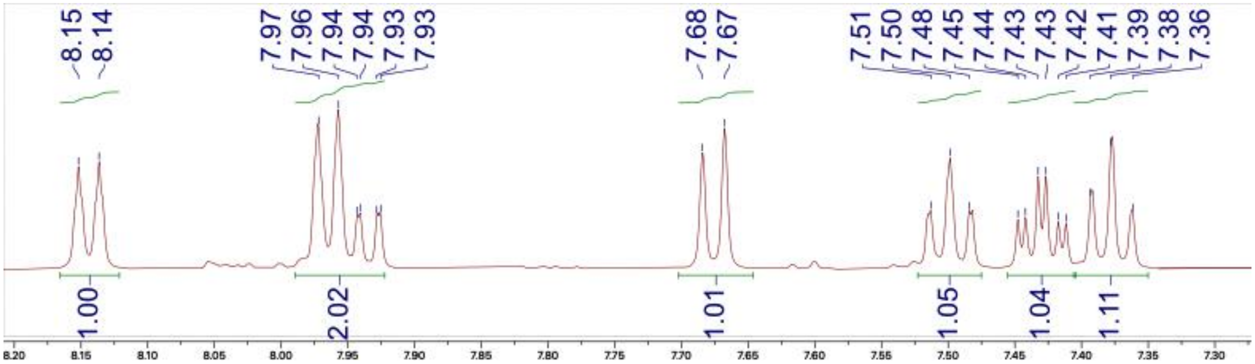

Figure S58: ^{1}H NMR ($CDCl_3$, 499.8 MHz) of compound **20**, aromatic region.

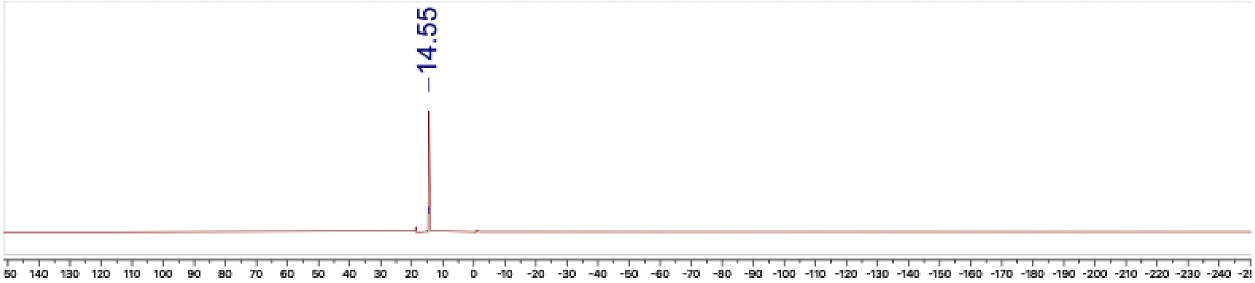

Figure S59: ^{31}P NMR ($CDCl_3$, 202.3 MHz) of compound **20**.

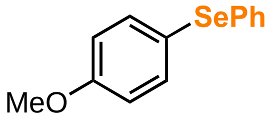

21

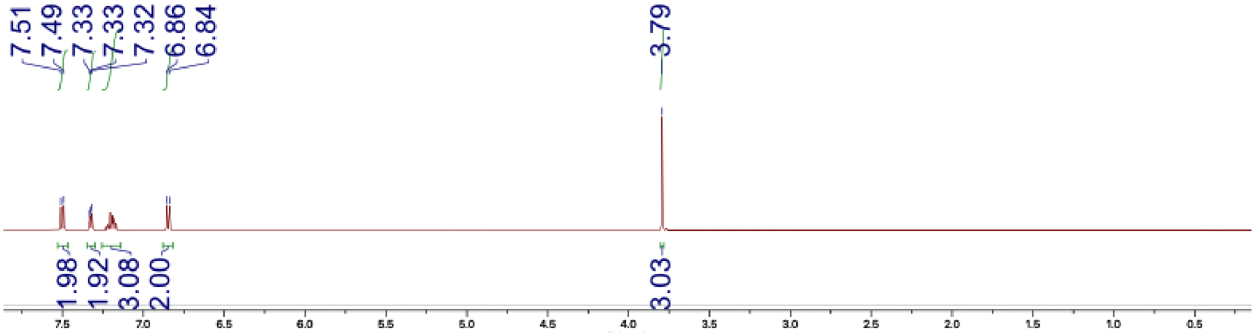

Figure S60: ^{1}H NMR ($CDCl_3$, 499.8 MHz) of compound **21**.

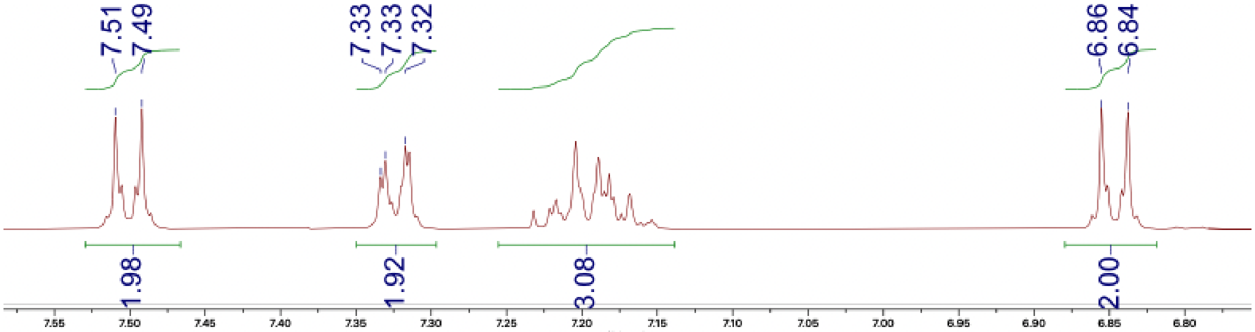

Figure S61: ^{1}H NMR ($CDCl_3$, 499.8 MHz) of compound **21**, aromatic region.

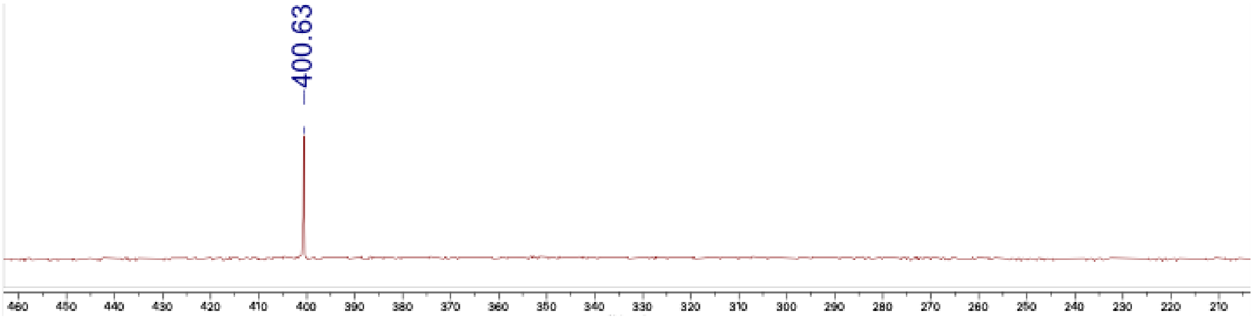

Figure S62: ^{77}Se NMR ($CDCl_3$, 114.5 MHz) of compound **21**.

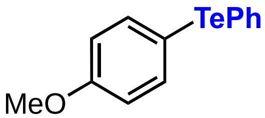

22

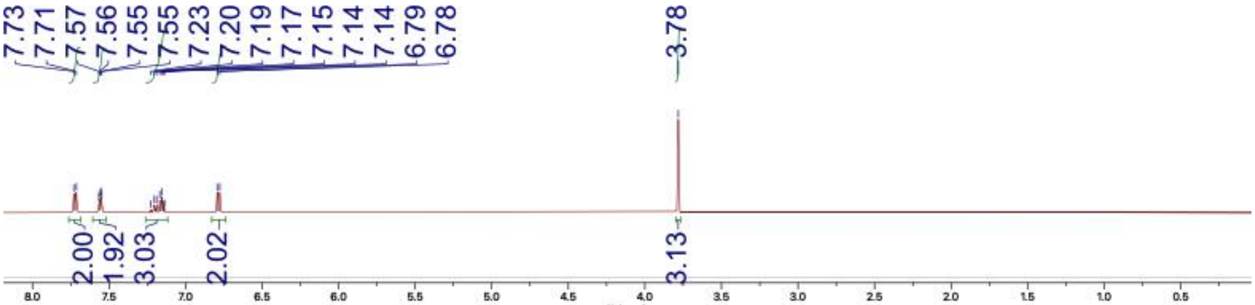

Figure S63: ^{1}H NMR ($CDCl_3$, 499.8 MHz) of compound **22**.

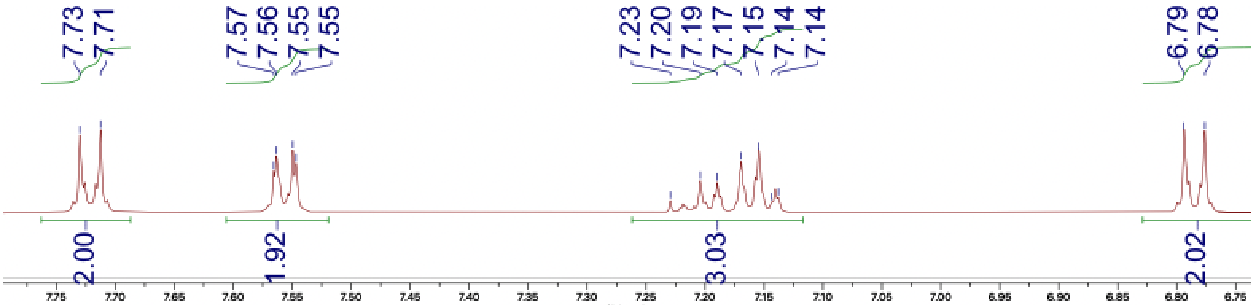

Figure S64: ^{1}H NMR ($CDCl_3$, 499.8 MHz) of compound **22**, aromatic region.

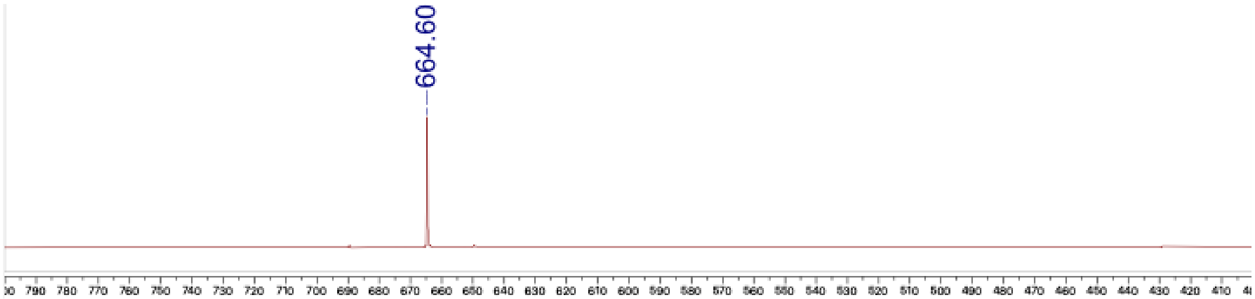

Figure S65: ^{125}Te NMR ($CDCl_3$, 189.5 MHz) of compound **22**.

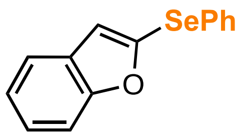

23

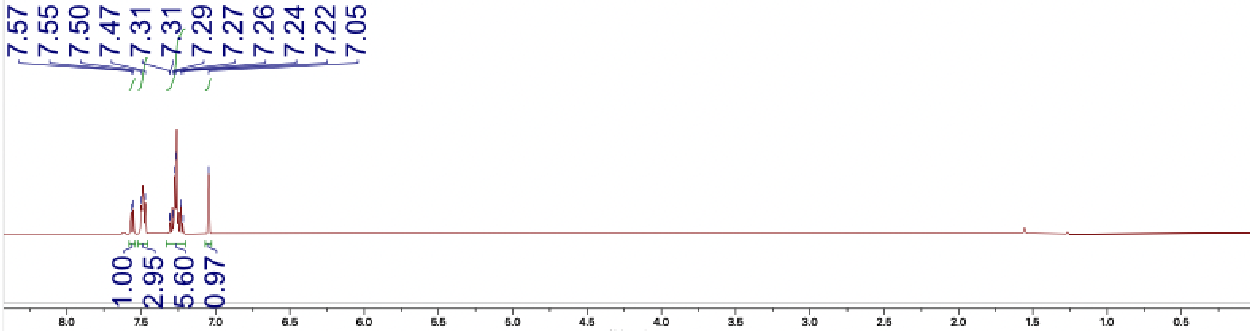

Figure S66: ^{1}H NMR ($CDCl_3$, 499.8 MHz) of compound **23.**

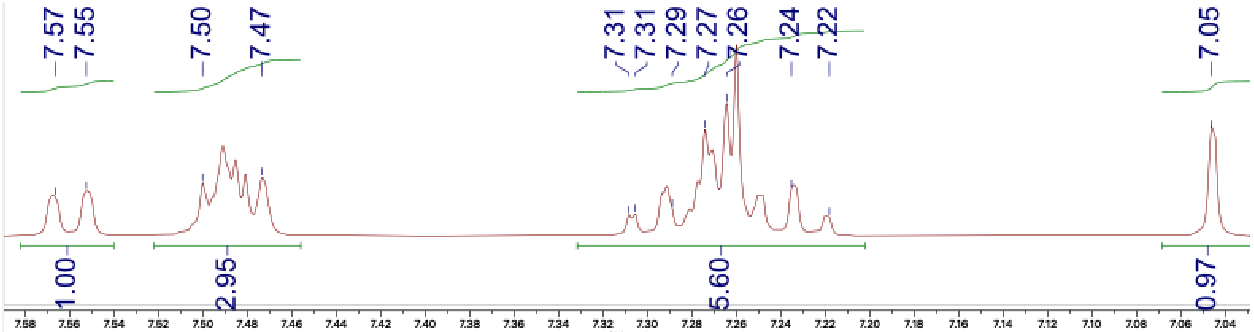

Figure S67: ^{1}H NMR ($CDCl_3$, 499.8 MHz) of compound **23**, aromatic region.

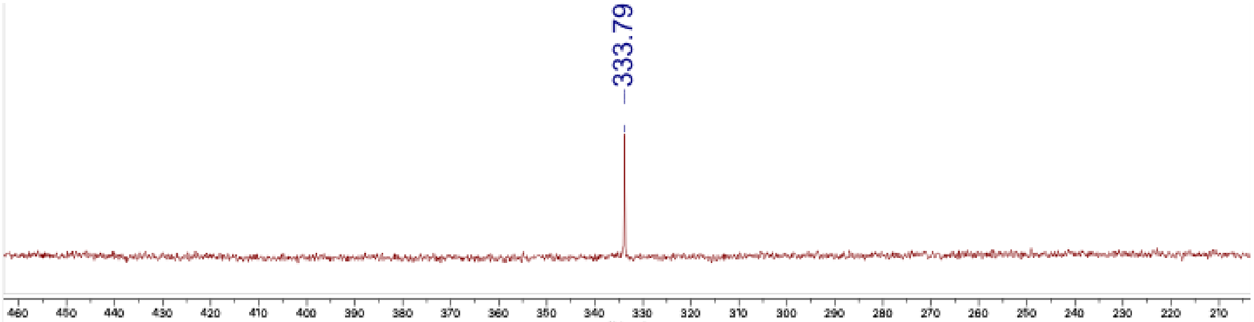

Figure S68: ^{77}Se NMR ($CDCl_3$, 114.5 MHz) of compound **23.**

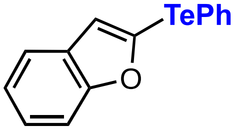

24

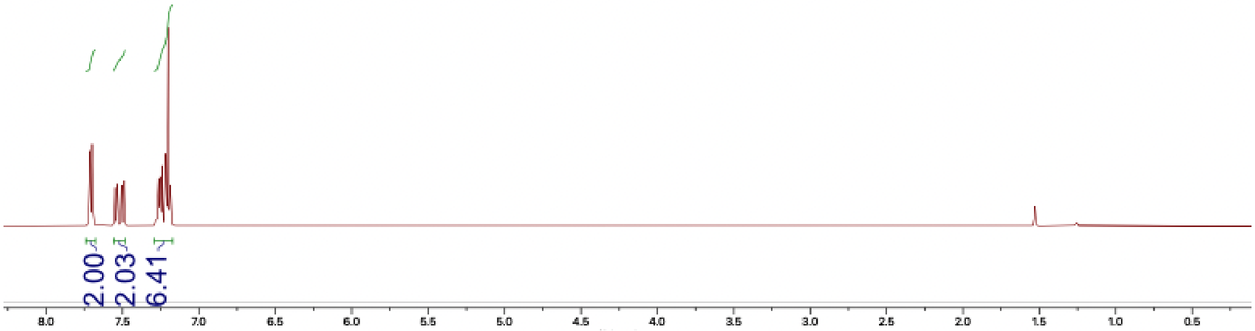

Figure S69: ^{1}H NMR ($CDCl_3$, 499.8 MHz) of compound **24**.

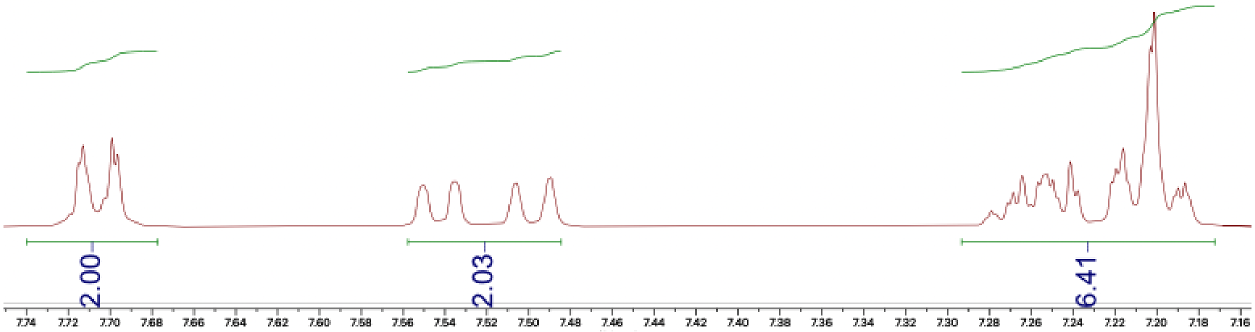

Figure S70: ^{1}H NMR ($CDCl_3$, 499.8 MHz) of compound **24**, aromatic region.

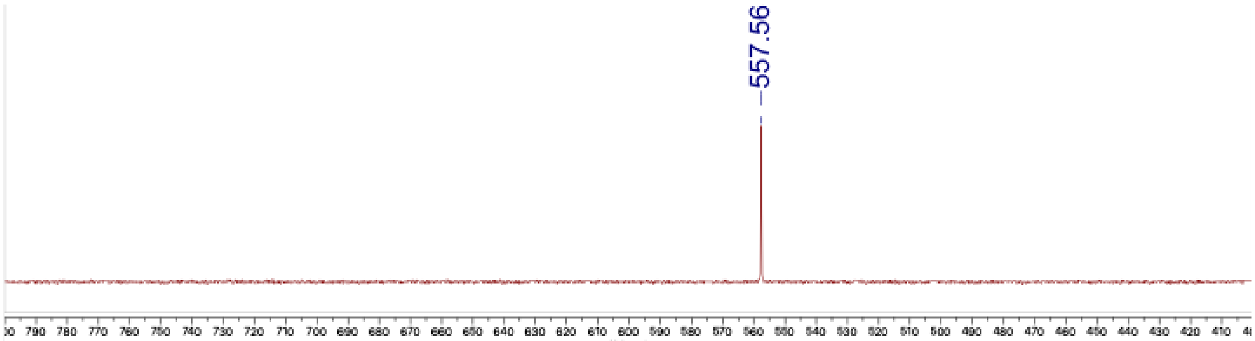

Figure S71: ^{125}Te NMR ($CDCl_3$, 189.5 MHz) of compound **24**.

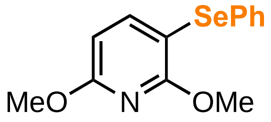

25

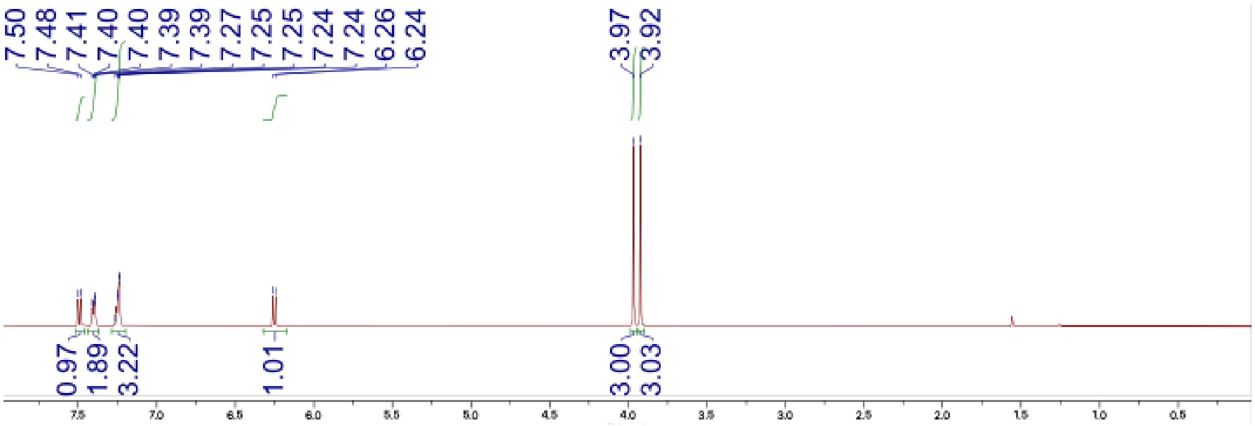

Figure S72: ^{1}H NMR ($CDCl_3$, 499.8 MHz) of compound **25**.

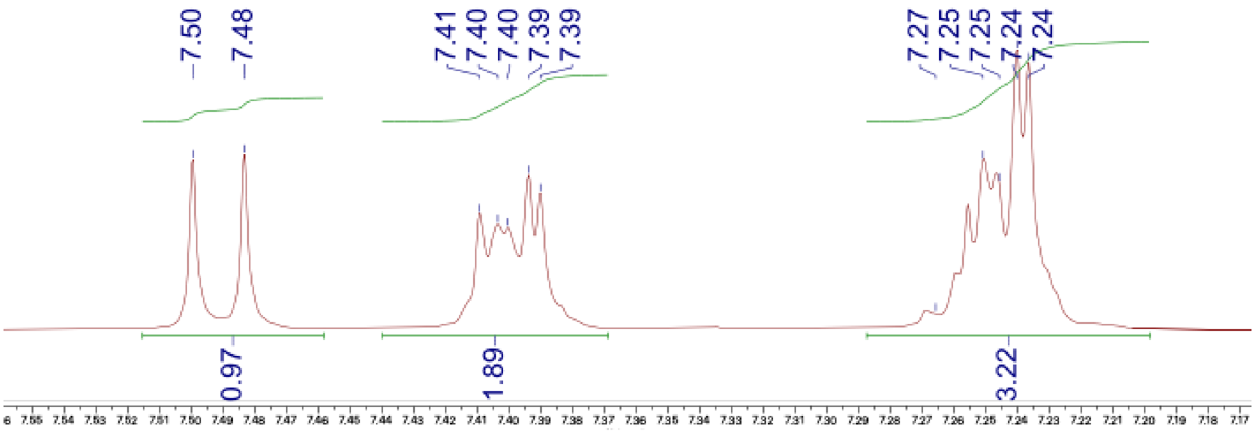

Figure S73: ^{1}H NMR ($CDCl_3$, 499.8 MHz) of compound **25**, aromatic region.

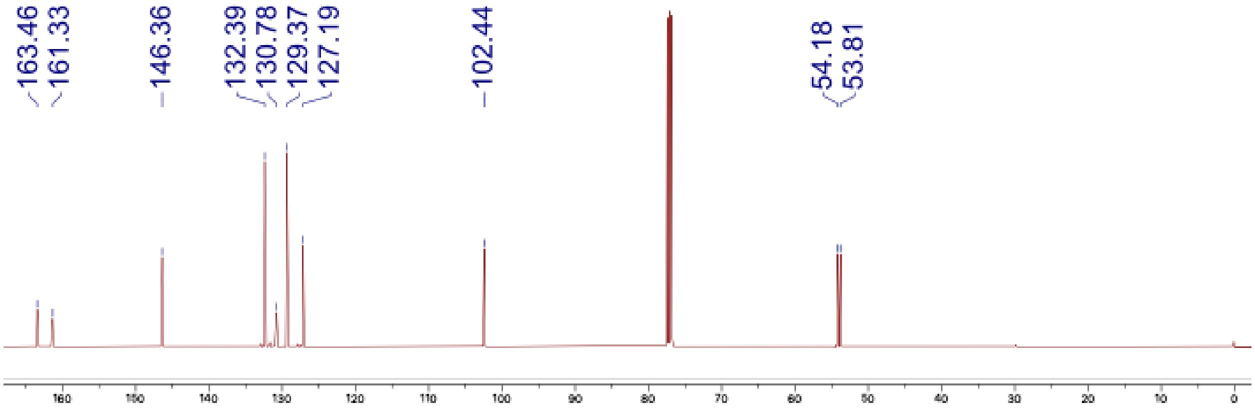

Figure S74: ^{13}C NMR ($CDCl_3$, 125.7 MHz) of compound **25**.

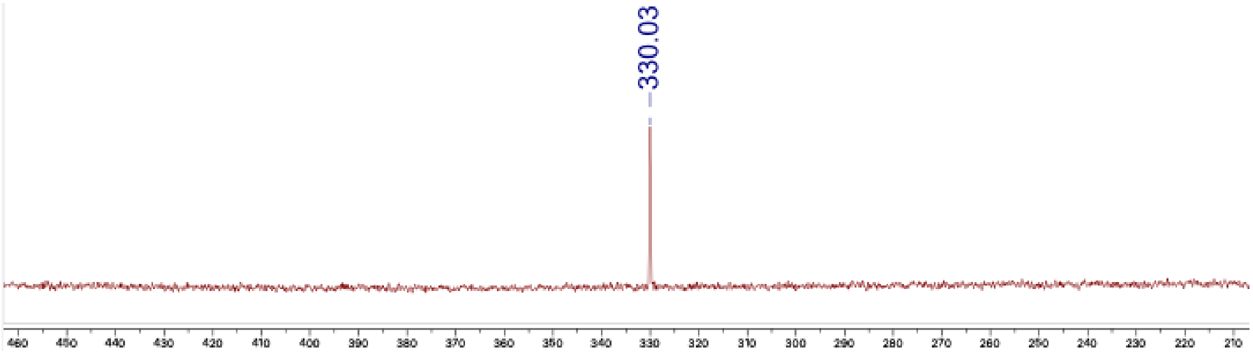

Figure S75: ^{77}Se NMR ($CDCl_3$, 114.5 MHz) of compound **25**.

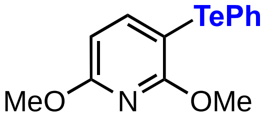

26

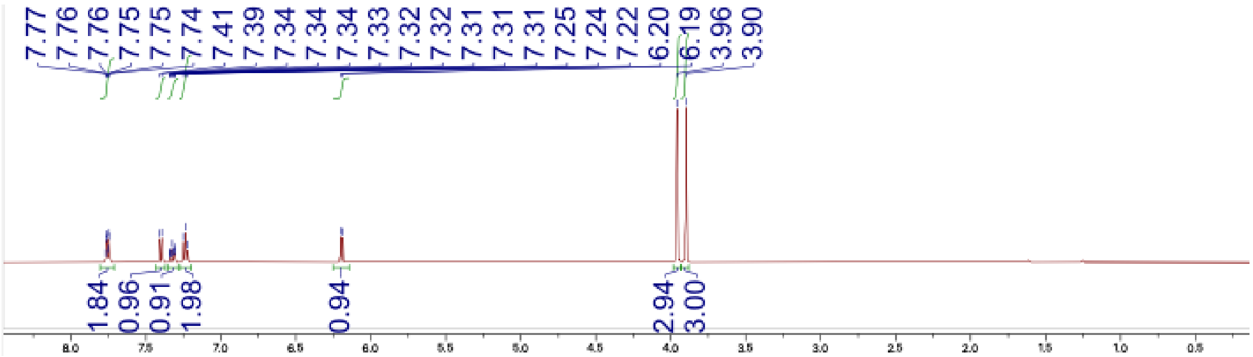

Figure S76: ^{1}H NMR ($CDCl_3$, 499.8 MHz) of compound **26**.

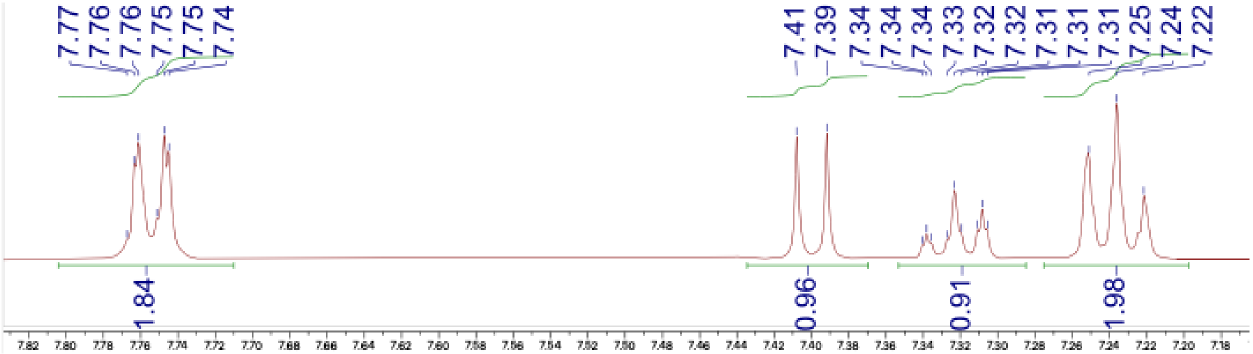

Figure S77: ^{1}H NMR ($CDCl_3$, 499.8 MHz) of compound **26**, aromatic region.

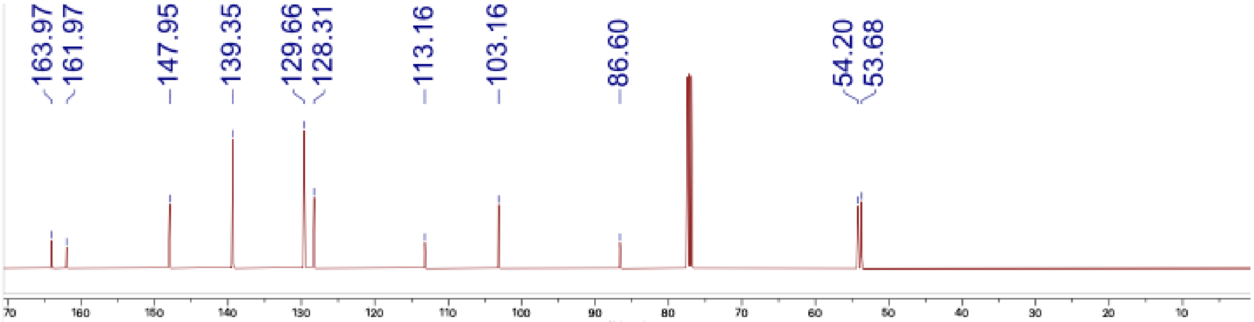

Figure S78: ^{13}C NMR ($CDCl_3$, 125.7 MHz) of compound **26**.

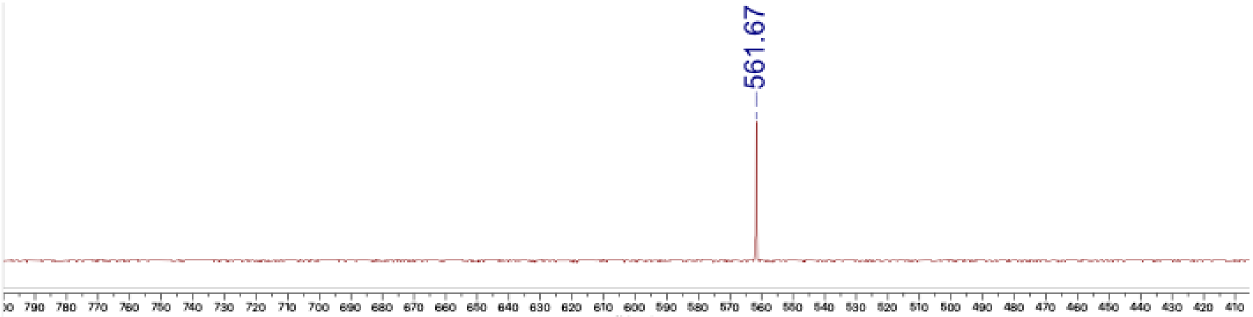

Figure S79: ^{125}Te NMR ($CDCl_3$, 189.5 MHz) of compound **26**.

8. References

[1] W. Xu, J.-P. Zou, W. Zhang, *Tetrahedron Letters* **2010**, *51*, 2639–2643.

[2] Y.-X. Chen, S. Zhang, Y.-J. Xue, L.-P. Mo, Z.-H. Zhang, *Applied Organometallic Chemistry* **2022**, *36*, e6480.

[3] T. Miao, L. Wang, *Advanced Synthesis & Catalysis* **2014**, *356*, 967–971.

[4] R. S. Shaikh, S. J. S. Düsel, B. König, *ACS Catal.* **2016**, *6*, 8410–8414.

[5] A. Gallego-Gamo, D. Reyes-Mesa, A. Guinart-Guillem, R. Pleixats, C. Gimbert-Suriñach, A. Vallribera, A. Granados, *RSC Adv.* **2023**, *13*, 23359–23364.

[6] R. Zhuang, J. Xu, Z. Cai, G. Tang, M. Fang, Y. Zhao, *Org. Lett.* **2011**, *13*, 2110–2113.

[7] M. Kalek, A. Ziadi, J. Stawinski, *Org. Lett.* **2008**, *10*, 4637–4640.

[8] C. Chen, J. Ding, L. Liu, Y. Huang, B. Zhu, *Adv Synth Catal* **2022**, *364*, 200–205.

[9] R. S. Shaikh, I. Ghosh, B. König, *Chemistry – A European Journal* **2017**, *23*, 12120–12124.

[10] C. Liu, M. Szostak, *Angew Chem Int Ed* **2017**, *56*, 12718–12722.

[11] F. Xu, O. M. Duke, D. Rojas, H. M. Eichelberger, R. S. Kim, T. B. Clark, D. A. Watson, *J. Am. Chem. Soc.* **2020**, *142*, 11988–11992.

[12] G. Kibriya, S. Mondal, A. Hajra, *Org. Lett.* **2018**, *20*, 7740–7743.

[13] N. Mukherjee, T. Chatterjee, B. C. Ranu, *J. Org. Chem.* **2013**, *78*, 11110–11114.

[14] J. Liu, W. Chen, L. Wang, *RSC Adv.* **2013**, *3*, 4723–4730.

[15] A. Kumar, S. Kumar, *Tetrahedron* **2014**, *70*, 1763–1772.
